# Supplementary material for: Cohort study of high maternal body mass index and the risk of adverse pregnancy and delivery outcomes in Scotland
Source: BMJ Open. 2020 Feb 20;10(2):e026168. doi: 10.1136/bmjopen-2018-026168 (PMC7045241; doi:10.1136/bmjopen-2018-026168)
Supplement: Supplementary data [file bmjopen-2018-026168supp001.pdf]

Supplementary file 1: Description of data fields used in the study

**Variable Name**	**Database Source**	**Variable Description/Values**	**Notes**
Mother_ID	N/A - study specific	Unique mother identifier	1516-0613/' followed by an anonymous identifier
Baby_ID	N/A - study specific	Unique baby identifier	Mother_ID followed by delivery sequence number followed by a baby sequence number. The baby sequence number for multiple babies from same delivery not necessarily in correct order due to missing CHI numbers.
Delivery_Seq_No	N/A - study specific	Delivery sequence number of mother	
Gest_Diabetes	SMR02	**1:** Yes, Gestational diabetes (diagnosed during this pregnancy)<br>**0:** Yes, Pre-existing diabetes (diagnosed before pregnancy) & No (no diabetes during this pregnancy)<br>**Missing:** Yes, Time of diagnosis unknown & Not Known	http://www.ndc.scot.nhs.uk/Dictionary-A-Z/Definitions/index.asp?Search=D&ID=214&Title=Diabetes
Gest_Hypertension	SMR02/SMR01	**1:** ICD 10 code O13.X<br>**0:** All other codes	Flagged codes cover gestational hypertension
Pre_Eclampsia	SMR02/SMR01	**1:** ICD 10 code O14._<br>**0:** All other codes	Flagged codes cover pre-eclampsia
Placental_Abruption	SMR02/SMR01	**1:** ICD 10 code O45._<br>**0:** All other codes	Flagged codes cover placental abruption
Placental_Praevia	SMR02/SMR01	**1:** ICD 10 code O44._<br>**0:** All other codes	Flagged codes cover placenta praevia

Postpartum_Haemorrhage	SMR02/SMR01	**1:** ICD 10 code O72._<br>**0:** All other codes	Flagged codes cover postpartum haemorrhage
Caesarean_Delivery	SMR02	**1:** Elective (planned) caesarean section & Emergency and unspecified caesarean section<br>**0:** All other codes	http://www.ndc.scot.nhs.uk/Dictionary-A-Z/Definitions/index.asp?Search=M&ID=322&Title=Mode of Delivery - Babies 1 to 3
Labour_Induction	SMR02	**1:** 1-8 - Induction of labour codes<br>**0:** 0, None<br>**Missing:** 9, Not known	http://www.ndc.scot.nhs.uk/Dictionary-A-Z/Definitions/index.asp?Search=I&ID=295&Title=Induction of Labour
SGA	SMR02	**1:** Birthweight ≤10th percentile<br>**0:** Birthweight >10th percentile	Small for gestational age flag
LGA	SMR02	**1:** Birthweight ≥90th percentile<br>**0:** Birthweight <90th percentile	Large for gestational age flag
Preterm_Delivery	SMR02	**1:** Estimated gestation < 37 weeks<br>**0:** Estimated gestation ≥ 37 weeks and ≤ 42 weeks<br>**Missing:** otherwise	http://www.ndc.scot.nhs.uk/Dictionary-A-Z/Definitions/index.asp?Search=E&ID=242&Title=Estimated Gestation
Postterm_Delivery	SMR02	**1:** Estimated gestation > 42 weeks<br>**0:** Estimated gestation ≥ 37 weeks and ≤ 42 weeks<br>**Missing:** otherwise	http://www.ndc.scot.nhs.uk/Dictionary-A-Z/Definitions/index.asp?Search=E&ID=242&Title=Estimated Gestation
Apgar_Score	SMR02	**1:** Apgar score at 5 mins < 7<br>**0:** Apgar score at 5 mins ≥ 7	http://www.ndc.scot.nhs.uk/Dictionary-A-Z/Definitions/index.asp?Search=A&ID=88&Title=Apgar Score - Babies 1 to 3
Maternal_Obesity	SMR02	**1:** Obese status<br>**0:** Overweight, Healthy or underweight status	Adults with BMI ≥ 30 classed as obese. BMI of girls aged 2 - 19 years old standardized using UK1990 growth reference values and z-score ≥ 6/3 (98th centile) classed as obese.

Maternal_Overweight_Obesity	SMR02	**1:** Overweight or Obese status<br>**0:** Healthy or underweight status	Adults with BMI ≥ 25 classed as overweight or obese. BMI of girls aged 2 - 19 years old standardized using UK1990 growth reference values and z-score ≥ 4/3 (91st centile) classed as overweight or obese.
Age	SMR02	Age of mother at delivery (in years)	
Parity	SMR02	Total number of previous pregnancies	http://www.ndc.scot.nhs.uk/Dictionary-A-Z/Definitions/index.asp?Search=P&ID=409&Title=Previous Pregnancies
Deprivation	SMR02	Carstairs 2001 quintiles for Scotland	1=least deprived; 5=most deprived
Smoking_Status	SMR02	**1:** Yes<br>**0:** No<br>**Missing:** Not known	http://www.ndc.scot.nhs.uk/Dictionary-A-Z/Definitions/index.asp?Search=S&ID=456&Title=Smoker During Pregnancy
Multiple_births	SMR02	**1:** More than one birth this pregnancy<br>**0:** Single birth	http://www.ndc.scot.nhs.uk/Dictionary-A-Z/Definitions/index.asp?Search=N&ID=349&Title=Number of Births this Pregnancy
Multiple_births_in_NRS	NRS Births	**1:** Multiple babies found for this mother's delivery in NRS Births	Multiple babies recorded in NRS Births but only a single baby recorded in SMR02
Previous_Caesarean_section	SMR02	**1:** More than zero<br>**0:** Zero	http://www.ndc.scot.nhs.uk/Dictionary-A-Z/Definitions/index.asp?Search=P&ID=406&Title=Previous Caesarean Sections
