# Supplementary figures and images for: Cohort study of high maternal body mass index and the risk of adverse pregnancy and delivery outcomes in Scotland
Source: BMJ Open. 2020 Feb 20;10(2):e026168. doi: 10.1136/bmjopen-2018-026168 (PMC7045241; doi:10.1136/bmjopen-2018-026168)

## Supplementary file 2. Study flow diagram

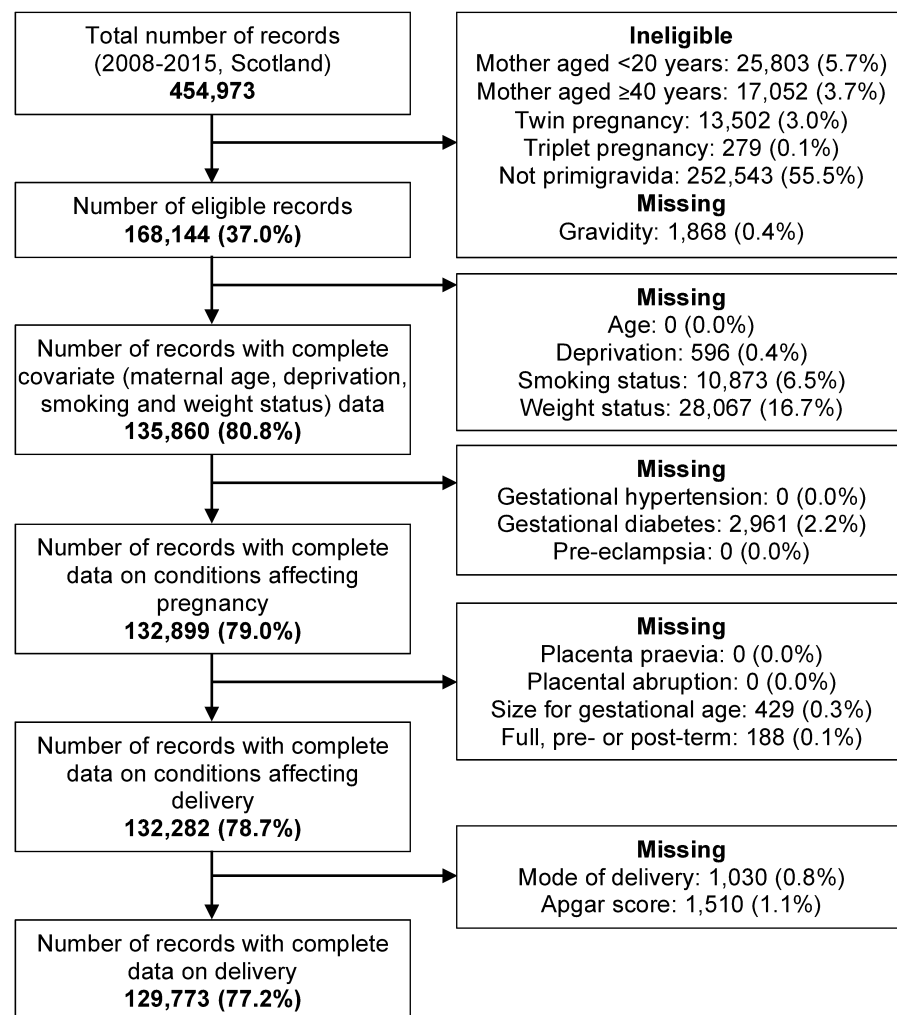

Supplement: Supplementary data [file bmjopen-2018-026168supp002.pdf]
