# Supplementary material for: Cohort study of high maternal body mass index and the risk of adverse pregnancy and delivery outcomes in Scotland
Source: BMJ Open. 2020 Feb 20;10(2):e026168. doi: 10.1136/bmjopen-2018-026168 (PMC7045241; doi:10.1136/bmjopen-2018-026168)
Supplement: Author's manuscript [file bmjopen-2018-026168.draft_revisions.pdf]

BMJ Open is committed to open peer review. As part of this commitment we make the peer review history of every article we publish publicly available.

When an article is published we post the peer reviewers' comments and the authors' responses online. We also post the versions of the paper that were used during peer review. These are the versions that the peer review comments apply to.

The versions of the paper that follow are the versions that were submitted during the peer review process. They are not the versions of record or the final published versions. They should not be cited or distributed as the published version of this manuscript.

BMJ Open is an open access journal and the full, final, typeset and author-corrected version of record of the manuscript is available on our site with no access controls, subscription charges or pay-per-view fees (http://bmjopen.bmj.com).

If you have any questions on BMJ Open's open peer review process please email info.bmjopen@bmj.com

High maternal Body Mass Index and the risk of adverse pregnancy, delivery and neonatal outcomes

Journal:	*BMJ Open*
Manuscript ID	bmjopen-2018-026168
Article Type:	Research
Date Submitted by the Author:	22-Aug-2018

Complete List of Authors:	Doi, Lawrence; University of Edinburgh, Scottish Collaboration for Public Health Research and Policy, School of Health in Social Science<br>Williams, Andrew James; University of Exeter<br>Marryat, Louise; University of Edinburgh, SCPHRP<br>Frank, John; University of Edinburgh, Scottish Collaboration for Public Health Research and Policy

Keywords:	OBSTETRICS, EPIDEMIOLOGY, PUBLIC HEALTH

SCHOLARONE™
Manuscripts

For peer review only

High maternal Body Mass Index and the risk of adverse pregnancy, delivery and neonatal outcomes

Lawrence Doi[1]*, Andrew James Williams[2], Louise Marryat[3], John Frank[3,4]

*Correspondence: larry.doi@ed.ac.uk

[1]Scottish Collaboration for Public Health Research and Policy, School of Health in Social Science, Doorway 6, Old Medical School, Teviot Place, University of Edinburgh, EH8 9AG, UK

[2]European Centre for Environment and Human Health, University of Exeter Medical School, Knowledge Spa, Royal Cornwall Hospital, Truro, Cornwall TR1 3HD, UK.

[3]Farr Institute at Scotland, University of Edinburgh, Nine, Edinburgh BioQuarter, 9 Little France Road, Edinburgh EH16 4UX, UK.

[4]Usher Institute of Population Health Sciences and Informatics, Doorway 1, Old Medical School, Teviot Place, University of Edinburgh, EH8 9AG, UK

For peer review only

Abstract

Objective: To examine the independent impact of high maternal weight status on pregnancy, delivery and neonatal outcomes.

Setting: Scotland

Participants: Data from 345,363 deliveries in Scotland between 2008 and 2015 were used. Women with overweight and obesity were compared with women with normal weight. Independent associations between maternal body mass index and pregnancy and neonatal outcomes were evaluated in a staged fashion, according to the clinical timelines by which pregnancy, delivery and neonatal outcomes occur.

Outcome measures: Maternal or pregnancy complications, delivery complications and neonatal outcomes.

Results: In the multivariable models controlling for potential covariates, we found that compared with women with normal weight, the odds of the following outcomes were significantly increased for women with overweight and obesity [overweight adjusted odds ratio; 95% confidence interval, followed by the same for women with obesity]: gestational hypertension [1.61; 1.52-1.70], [2.64; 2.50-2.80]; gestational diabetes [2.34; 2.25-2.65], [8.69; 8.90-9.34]; pre-eclampsia [1.42; 1.32-1.54], [2.11; 1.95-2.28]; labour induction [1.26; 1.24-1.36], [1.71; 1.67-1.75]; and emergency caesarean section [1.78; 1.72-1.86], [3.15; 3.03-3.28].

Conclusions: Women with overweight and obesity in Scotland are at greater risk of adverse pregnancy, delivery and neonatal outcomes. The risk of these conditions increases steadily with increasing body mass index. Health professionals should be empowered to deliver promising dietary and lifestyle interventions to women at risk

For peer review only - http://bmjopen.bmj.com/site/about/guidelines.xhtml

of overweight and obesity prior to conception, and control excessive weight gain in pregnancy.

Strengths and limitations of this study

- This study used a large, retrospective, national database covering all major maternal and neonatal outcomes in Scotland.
- The staged analysis approach employed ensured an estimate of the independent impact of high maternal-weight status on each outcome.
- All women with BMI of 30 or more were considered as having obesity and it is likely that differentiating morbid obesity or obesity class II and III from women with obesity would have generated more precise estimates.
- Although the analysis was restricted to only single births, the nature of the analysis meant that women who may have had more than one delivery during the study period contributed data on more than one birth, so not all outcomes were strictly independent.

For peer review only

For peer review only - http://bmjopen.bmj.com/site/about/guidelines.xhtml

Introduction

The increasing global prevalence of overweight and obesity makes it probable that a growing number of women with high body mass index (BMI) are becoming pregnant. High maternal BMI during pregnancy has adverse health implications for women (1,2,3) and predisposes the unborn child to adverse outcomes, including neonatal deaths, stillbirth, and admission to a neonatal unit (4,5,6). Wider service and economic consequences can also be seen (7,8,9): there are indirect burdens of illness and associated costs, in that women with high BMI who experience adverse birth outcomes such as neonatal death or stillbirth may require support from family and friends as well as bereavement support services within the community. There are also substantial healthcare cost implications for infants who may require neonatal intensive care unit admission.

Maternal weight status is currently high in Scotland: a recent study reported that 31.5% of mothers were overweight and a further 23.6% were affected by obesity (10). However, no recent study in Scotland has investigated the effect of high maternal BMI on pregnancy and neonatal outcomes. The aim of this study was to examine the independent associations between high maternal BMI during pregnancy and perinatal risks, including adverse neonatal outcomes. Understanding of these associations can highlight areas where prevention strategies could be targeted.

Methods

Study population and data sources

For peer review only - http://bmjopen.bmj.com/site/about/guidelines.xhtml

This retrospective study used data from 362,102 women of which outcome data were available for 345,363 deliveries in Scotland between January 2008 and December 2015. The women and infants were identified within the Scottish Morbidity Record (SMR) 01 and 02 and Scottish Birth Record (SBR). SMR01 is generated for patients receiving inpatient or day care in the General or Acute specialties, whilst SMR02 is generated for patients receiving inpatient or day care in the Obstetric Specialties. The SBR records all of a baby's neonatal care in Scotland. The outcome variables are recorded in these registries according the World Health Organisation's International Classification of Diseases, Tenth Revision (ICD-10). Further description of the content of these registries is available (11). The study was designed as a clinical audit so did not require approval from a Research Ethics Committee. However, approval was obtained from the national Electronic Data Research and Innovation Service to use the anonymised data collected by these registries.

Patient and public involvement

Patients were not involved in the design, analysis and interpretation of this study.

Exposure variable

More than 80% of pregnant women in Scotland present themselves for antenatal care during the first trimester of their pregnancy. Height and weight are measured by the midwife at the first antenatal visit, typically before 12 weeks of pregnancy. Body mass index was calculated using the formula weight (kg)/height (m^2). BMI completeness was 69% in 2008 but this increased gradually to 87% in 2011 when recording of weight and height became mandatory. By 2015 BMI completeness was 98%. Women were categorised into three BMI groups as described in Table 1.

For peer review only - http://bmjopen.bmj.com/site/about/guidelines.xhtml

Table 1. BMI definitions used

Variable	Category	BMI Definition
Maternal weight status	Normal	<25 kg/m^2 (for women over 19);<br>>0.4th centile to <91st centile<br>(for girls aged 15 - 19 years old)
	Overweight	≥25 kg/m^2 to <30 kg/m^2 (for women over 19);<br>≥91st centile to <98th centile<br>(for girls aged 15 - 19 years old)
	Obesity	≥30 kg/m^2 (for women over 19);<br>≥98th centile (for girls aged 15 - 19 years old)

* Age and gender standardised using UK1990 growth reference values

Outcomes

Outcome measures included were maternal or pregnancy complications such as gestational diabetes, gestational hypertension, pre-eclampsia, placenta praevia, placental abruption and postpartum haemorrhage. Delivery complications studied were induction of labour, caesarean delivery (includes elective and emergency caesarean sections), pre-term delivery (defined as less than 37 weeks of gestation) and post-term delivery (more than 42 weeks of gestation).

Neonatal outcomes studied were: low Apgar score (less than "7" at 5 minutes), stillbirth, congenital anomalies, neonatal intensive care unit (NICU) admission, neonatal death, small for gestational age (SGA), and large for gestational age (LGA). SGA were infants with birthweight of ≤10th percentile for gestational age according to UK1990 growth reference curve (12,13), and those with LGA were infants with birth weight ≥90th percentile.

Covariates

For peer review only - http://bmjopen.bmj.com/site/about/guidelines.xhtml

Maternal age at delivery, parity, smoking during pregnancy and Scottish Index of Multiple Deprivation for the datazone of the mother's residence at birth, were considered as potentially confounding variables and were included as covariates in the adjusted analyses. Table 2 describes the covariates used in this study by maternal weight status among singleton pregnancies (a pregnancy with one fetus as opposed to twins or multiples). The data in Table 2 show the numbers of women who had data on age, parity, deprivation and maternal smoking. This means that the total number of women is slightly larger than the number in stage one of our analysis, as there were women missing hypertension, diabetes or pre-eclampsia data.

For peer review only

Table 2. Maternal characteristics among women with normal weight, overweight and obesity+ (singleton pregnancies)

	Normal* N = 184,186		Overweight* N = 100,844		Obesity* N = 77,072	
	N	%	N	%	N	%
**Maternal age (y)**						
15-19	12,887	7.0	3,737	3.7	3,586	4.7
20-24	34,067	18.5	16,621	16.5	13,638	17.7
25-29	50,599	27.5	27,648	27.4	22,055	28.6
30-34	53,604	29.1	30,389	30.1	21,786	28.3
35-39	27,339	14.8	18,108	18.0	12,730	16.5
40-44	5,433	3.0	4,105	4.1	3,131	4.1
44-49	257	0.1	191	0.2	146	0.2
**Parity**						
1	85,617	46.5	41,205	40.9	29,420	38.2
2	64,905	35.2	37,016	36.7	27,935	36.3
3+	33,664	18.3	22,623	22.4	19,717	25.6
**Deprivation**						
Q1 (Least deprived)	36,229	19.7	17,964	17.8	10,675	13.9
Q2	34,098	18.5	18,343	18.2	13,223	17.2
Q3	37,016	20.1	20,286	20.1	15,495	20.1
Q4	38,945	21.1	22,236	22.1	18,449	23.9
Q5 (Most deprived)	37,898	20.6	22,015	21.8	19,230	25.0
**Maternal smoking in pregnancy**						
No	148,334	80.5	83,231	82.5	62,303	80.8
Yes	35,852	19.5	17,613	17.5	14,769	19.2

*Maternal weight status at first antenatal visit
+Figures show women who had complete data on age, parity, derivation and maternal smoking

Data analysis

Using Stata 14 (14), logistic regression was used to calculate odds ratios (ORs). BMI groups with overweight and obesity were compared with the normal BMI group (the reference population). A confidence interval (CI) of 95% was produced for all ORs. The analysis of the outcomes proceeded in a staged fashion according to the clinical timelines by which pregnancy, delivery and neonatal outcomes occur.

Firstly, all conditions occurring during pregnancy such as gestational hypertension, gestational diabetes and pre-eclampsia, which may also be a risk factor for later outcomes, were compared with a 'healthy' group who did not have any of these conditions. The second stage of the analysis considered only the group with none of the stage one conditions. Placental abruption and placenta praevia were considered within this stage because the risk of both conditions is associated with one or more of the stage one conditions. Therefore, by limiting the dataset to those without any of the stage one conditions, a better estimate of the *independent* impact of maternal weight status is established. This approach means the sample size reduces steadily as one moves through the process. The stages used for the analyses are outlined in Figure 1. In the final stage there are two sub-stages because postpartum haemorrhage is a maternal outcome and we wanted to treat this outcome independent of NICU admission, congenital anomaly and neonatal death.

<Figure 1>

Results

For peer review only - http://bmjopen.bmj.com/site/about/guidelines.xhtml

Within our study population 50.9% of pregnant women were categorised as normal weight, 27.8% overweight and 21.3% were affected by obesity. The socio-demographic characteristics of the women in the three BMI categories are presented in Table 2. A comparison between the women in the various BMI categories shows that the women who were affected by overweight and obesity were slightly older and more often multiparous. Maternal smoking was slightly higher among women with normal weight than in women with overweight and obesity. Among the normal BMI group 20.6% were from the most deprived and 19.7% were from the least deprived group. However, the difference in social deprivation was more marked within women with obesity. Among this group, 25.0% were from the most deprived group whilst 13.9% were from the least deprived group.

Table 3 shows odd ratios for pregnancy and delivery complications, as well as neonatal outcomes, among women with overweight and obesity, and with singleton births. The three conditions occurring during pregnancy increased steadily with increasing BMI. For example, compared to the normal BMI group, the risk of gestational diabetes was 2.34 (95% CI: 2.25-2.65) but among the women with obesity the risk was increased almost 9-fold (95% CI: 8.09-9.34). Relative to women with normal weight, the adjusted OR of pre-eclampsia for women with overweight was 1.42 (95% CI: 1.32-1.54) and 2.11 (95% CI: 1.95-2.28) for women with obesity.

For peer review only - http://bmjopen.bmj.com/site/about/guidelines.xhtml

Table 3. Pregnancy and delivery complications and neonatal outcomes among women with normal, overweight and obesity (singletons)

	Sample	Controls N (%)	Overweight N N (%)	Adjusted OR* (95% CI)	Obesity N (%)	Adjusted OR* (95% CI)
**Stage 1: Conditions occurring during pregnancy**						
Gestational hypertension	345,363	177,977 (51.5)	96,384 (27.9)	1.61 (1.52, 1.70)	71,002 (20.6)	2.64 (2.50, 2.80)
Gestational diabetes	343,778	176,268 (51.3)	95,523 (27.8)	2.34 (2.25, 2.65)	71,987 (20.9)	8.69 (8.09, 9.34)
Pre-eclampsia	341,835	176,866 (51.7)	95,295 (27.9)	1.42 (1.32, 1.54)	69,674 (20.4)	2.11 (1.95, 2.28)
**Stage 2: Conditions affecting delivery**						
Placenta praevia	337,325	174,973 (51.9)	93,997 (27.9)	1.18 (1.01, 1.39)	68,355 (20.3)	0.90 (0.74, 1.09)
Placental abruption[a]	337,158	174,930 (51.9)	93,892 (27.9)	0.86 (0.71, 1.04)	68,336 (20.3)	0.91 (0.74, 1.13)
**Stage 3: Delivery**						
Induction of labour	237,216	131,077 (55.3)	63,992 (27.0)	1.26 (1.24, 1.29)	42,147 (17.8)	1.71 (1.67, 1.75)
Caesarean section	216,243	120,687 (55.8)	58,361 (27.0)	1.33 (1.29, 1.36)	37,195 (17.2)	1.84 (1.79, 1.90)
Emergency caesarean section	193,240	108,075 (55.9)	51,924 (26,9)	1.78 (1.72, 1.86)	33,241 (17.2)	3.15 (3.03, 3.28)
**Stage 4: Birth outcome**						
Apgar score	175,492	101,091 (57.6)	46,480 (26,5)	0.97 (0.94, 0.99)	27,921 (15,9)	0.92 (0.89, 0.96)
Stillbirth[b]	145,927	83,787 (57.4)	38,720 (26.5)	1.24 (0.94, 1.64)	23,420 (16.1)	2.09 (1.58, 2.76)
**Stage 5: Term and size**						
Small for gestational age	94,599	56,604 (59.8)	24,218 (25.6)	0.81 (0.77, 0.86)	13,777 (14.6)	0.74 (0.69, 0.79)
Preterm	90,697	53,780 (59.3)	23,447 (25.9)	0.97 (0.90, 1.04)	13,470 (14.9)	1.04 (0.96, 1.13)
Small for gestational age and preterm[c]	86,078	51,040 (59.3)	22,291 (25.9)	0.67 (0.46, 0.98)	12,747 (14.8)	0.53 (0.31, 0.90)
Postterm[d]	85,950	50,947 (59.3)	22,265 (25.9)	0.83 (0.37, 1.87)	12,738 (14.8)	1.27 (0.54, 3.00)
Large for gestational age	129,770	73,822 (56.9)	34,865 (26.9)	1.23 (1.20, 1.26)	21,083 (16.3)	1.44 (1.40, 1.49)
**Stage 6: Postnatal outcomes (infant and maternal)**						
NICU	85,421	50,651 (59.3)	22,117 (25.9)	1.08 (0.99, 1.19)	12,653 (14.8)	1.31 (1.18, 1.45)
Neonatal death[e]	82,682	49,123 (59.4)	21,401 (25.9)	0.68 (0.22, 2.12)	12,158 (14.7)	0.29 (0.04, 2.21)
Congenital anomaly[f]	46,441	27,738 (59.7)	11,932 (25.7)	1.37 (0.95, 1.96)	6,771 (14.6)	1.25 (0.80, 1.94)
Postpartum haemorrhage	85,913	50,925 (59.3)	22,257 (25.9)	0.99 (0.94, 1.03)	12,731 (14.8)	1.04 (0.98, 1.10)

OR = Odd Ratio; CI = Confidence Interval; NICU = Neonatal Intensive Care Unit
*Adjusted for maternal age, parity, deprivation and smoking in pregnancy
aThere were no placental abruptions among mothers aged 45-49 years, so 531 observations were dropped
bThere were no stillbirths to mothers aged 45-49 years, so 72 observations were dropped
cThere were no preterm and small for gestation age babies born to mothers aged 45-49 years, so 36 observations were dropped
dThere were no post-term babies born to mothers aged 45-49 years, so 36 observations were dropped
eThere were no neonatal deaths among births from mothers aged 45-49 years, so 35 observations were dropped
[f]There were no congenital anomalies among births from mothers aged 45-49 years, so 16 observations were dropped

For peer review only - http://bmjopen.bmj.com/site/about/guidelines.xhtml

Regarding conditions affecting delivery, the risk of placenta praevia was slightly increased among women with overweight as compared with normal weight women (OR 1.18, 95% CI: 1.01-1.39). However, among women with obesity, the OR was not significant, likely due to reduced statistical power for this uncommon outcome. The odds of experiencing placental abruption were not significantly different among the different BMI categories.

The chance of induction of labour and caesarean section, either elective or emergency increased with increasing BMI. Women with overweight had odds of 1.33 (95% CI: 1.29-1.36) higher for having an elective caesarean section and higher odds (OR 1.78, 95% CI: 1.72-1.86) for undergoing emergency caesarean section compared with normal weight women. The corresponding ORs for women with obesity were 1.84 (95% CI: 1.79-1.90) and 3.15 (95% CI: 3.03-3.28).

In terms of birth outcomes, the risk of stillbirth was not significantly different for women with overweight relative to normal weight women. However, there was about 2-fold increased risk among women with obesity. Being with overweight (OR 0.97, 95% CI: 0.94-0.99) or obesity (OR 0.92, 95% CI: 0.89-0.96) significantly decreased the risk of low Apgar score, likely because women with obesity and overweight may receive increased monitoring, which means issues are identified and managed earlier to reduce any fetal distress in labour.

In contrast with the normal BMI group, births to women with overweight and obesity were associated with decreased risk of small-for-gestational age and pre-term delivery, ORs 0.67 (95% CI: 0.46-0.98) and 0.53 (95% CI: 0.31-0.90) respectively. However, the prevalence of large-for-gestational age increased among women with

For peer review only - http://bmjopen.bmj.com/site/about/guidelines.xhtml

overweight, OR of 1.23 (95%CI: 1.20-1.26) and obesity, OR 1.44 (95% CI: 1.40-1.49) compared with women with normal weight.

There were no statistically significant differences in ORs across the three BMI groups when postnatal outcomes were considered, with the exception of NICU admission. The odds of a baby being admitted to NICU was 1.31 times (95% CI: 1.18-1.45) higher among women with obesity in contrast to women with normal weight.

Discussion

In this large, retrospective study using a staged approach to analysis, we found that women with overweight or obesity during pregnancy were at increased risk of several adverse pregnancy and delivery complications, and as well as poor neonatal outcomes. Some earlier studies have also found similar findings, although most studies to date have tended to focus on the association between women with obesity (not including overweight) and these outcomes. However, a greater number of women are likely to be with overweight rather than obesity, so it is important to also understand the impact of overweight on pregnancy and neonatal outcomes.

In terms of the association between high maternal BMI and conditions that occur during pregnancy, we found that the risk of all the conditions considered (gestational hypertension, gestational diabetes and pre-eclampsia) increased steadily with increasing BMI, which is in line with similar studies (3,4,5). A study compared women

For peer review only - http://bmjopen.bmj.com/site/about/guidelines.xhtml
For peer review only

with normal weight to women with morbid obesity (BMI greater than 40), and also found that there was an increased risk of pre-eclampsia (OR 4.82; 95% CI: 4.04-5.74) (4). Our study also found that, aside from heightened pre-eclampsia risk for women with obesity, being overweight was also significantly associated with this outcome, albeit to a lesser degree. A meta-analysis of the association between maternal BMI and the risk of pre-eclampsia showed that the risk doubled with each 5-7 kg/m^2 increase in pre-pregnancy BMI (15). It is evident that the risk of pre-eclampsia increases with weight gain; therefore preventative strategies should be focussed on getting women, especially those already overweight, to reduce weight prior to conception. Weight loss in pregnancy requires careful management in order to avoid unintended consequences (16). Nevertheless, women often engage with health professionals during pregnancy; therefore dietary and lifestyle interventions such as physical activity, which have been shown by reviews and meta-analyses (16,17) to reduce gestational weight gain and improve outcomes for both mother and baby, could be provided to them.

Generally, rates of caesarean delivery have increased significantly across many developed countries in recent years (18). Our study found that women with overweight and obesity showed an increased risk of caesarean delivery (both elective and emergency) compared to normal-weight women, but we note that the overall frequency of caesarean delivery across our obstetric population seems quite high, compared to a previous Swedish study (4). Possible reasons could be that caesarean delivery is probably less risky now, due to advances in medical science, which facilitate accurate monitoring of the progress of labour and fetal intra-partum condition (19). Nevertheless, this is quite concerning for Scotland, which has

For peer review only - http://bmjopen.bmj.com/site/about/guidelines.xhtml

invested in programmes aimed at promoting natural birth such as keeping childbirth natural and dynamic (KCND). The KCND is a maternity care programme introduced by the Scottish Government with the aim of maximising opportunities for women to have as natural a birth experience as possible, reduce unnecessary interventions in low-risk pregnancy and childbirth, and to provide women-centred care (20,21,22).

We found that stillbirth was significantly associated with obesity and not overweight. Scott-Pillai et al. (5) observed that women with overweight were not at increased risk of stillbirth, neither were class I (BMI 30.00-34.99) and II (BMI 35.00-39.99) obesity. However, women in obesity class III (BMI ≥40) were at increased risk of stillbirth. Cedergren (4) also found that stillbirth was significantly associated with both obesity and morbid obesity. Our study also found no significant association between high maternal BMI and neonatal death. This was also in contrast to a previous study (4). Since caesarean deliveries were quite frequent among our obstetric study population as compared with this study, it is likely that health professionals in Scotland are probably intervening early with regards to problems in labour in women with overweight and obesity, in order to reduce fetal distress. This early intervention in labour to reduce fetal distress could also possibly explain our findings that overweight or obesity was significantly associated with decreased risk of low Apgar score.

This study found that births to women with overweight and obesity were associated with decreased risk of small-for-gestational age and pre-term delivery, compared with women with normal weight. This an unexpected protective effect and we suspect that excess weight in pregnancy may shift the entire birth weight distribution

For peer review only - http://bmjopen.bmj.com/site/about/guidelines.xhtml

upwards, perhaps through hormonal mechanisms that operate in full-blown cases of macrosomia in infants of diabetic mothers. Further studies may be required to enhance our understanding of the mechanism by which different maternal weight categories affect stillbirth and neonatal death.

Strengths and limitations

This study used a large, retrospective, national database covering all major maternal and neonatal outcomes and the staged analysis approach we employed ensured an estimate of the independent impact of high maternal-weight status on each outcome. The dataset we used combined underweight and normal weight women as normal BMI group. Using this as reference group might have strengthened the association between high maternal BMI and the pregnancy and neonatal outcomes considered. However, only a very small number of women are underweight during pregnancy in Scotland in recent years. Also, some studies differentiate between the different obesity categories, but in this study all women with BMI of 30 or more were considered as having obesity. It is likely that differentiating morbid obesity or obesity class II and III from women with obesity would have generated additional insight. Although we restricted the analysis to only single births, the nature of the analysis meant that women who may have had more than one delivery during the study period contributed data on more than one birth, so not all outcomes were strictly independent.

Conclusion

This study has shown that women with overweight and obesity in Scotland are at greater risk of several pregnancy and delivery complications including gestational

For peer review only - http://bmjopen.bmj.com/site/about/guidelines.xhtml

hypertension, gestational diabetes, pre-eclampsia, labour induction and caesarean delivery. The risk of these conditions increases steadily with increasing BMI. Women with obesity also had 2-fold risk increase for stillbirth. Health professionals should be empowered to deliver promising dietary and lifestyle interventions to women at risk of overweight and obesity prior to conception, and control excessive weight gain in pregnancy.

For peer review only

For peer review only - http://bmjopen.bmj.com/site/about/guidelines.xhtml

References

1. Rooney BL, Schauberger CW. Excess pregnancy weight gain and long-term obesity: one decade later. *Obstet Gynecol* 2002; **100(2):** 245-252.
2. Wilson BJ, Watson MS, Prescott GJ, Sunderland S, Campbell DM, Hannaford P, Smith WCS. Hypertensive diseases of pregnancy and risk of hypertension and stroke in later life: results from cohort study. *BMJ* 2002; **326:** 845–849.
3. Kumari P, Gupta M, Kahlon P, Malviya S. Association between high maternal body mass index and fetomaternal Outcome. *J Obes Metab Res* 2014; **1:** 143-148.
4. Cedergren MI. Maternal morbid obesity and the risk of adverse pregnancy outcome. *Obstetricians and Gynecologists* 2004; **103:** 219-224.
5. Scott-Pillai R, Spence D, Cardwell C, Hunter A, Holmes V. The impact of body mass index on maternal and neonatal outcomes: a retrospective study in a UK obstetric population, 2004–2011. *BJOG* 2013; **120:** 932–939.
6. Rahman MM, Abe SK, Kanda M, Narita S, Rahman MS, Bilano V, Ota E, Gilmour S, Shibuya K. Maternal body mass index and the risk of birth and maternal health outcomes in low- and middle-income countries: a systematic review and meta-analysis. *Obesity Reviews* 2015; **16:** 758–770.
7. Heslehurts N, Lang R, Rankin J, Wilkinson J, Summerbell CD. Obesity in pregnancy: a study of the impact of maternal obesity on NHS maternity services. *BJOG* 2007; **114(3):** 334-342.
8. Furness PJ, McSeveny K, Arden MA, Garland C, Dearden AM, Soltani H. Maternal obesity support services: a qualitative study of the perspectives of women and midwives. *BMC Pregnancy and Childbirth* 2011; **11:** 69.

For peer review only - http://bmjopen.bmj.com/site/about/guidelines.xhtml

9. Lenoir-Wijnkoop I, van der Beek EM, J Garssen, Nuijten MJC, Uauy RD. Health economic modeling to assess short-term costs of maternal overweight, gestational diabetes, and related macrosomia – a pilot evaluation. *Front Pharmacol* 2015; **6:**103. doi: 10.3389/fphar.2015.00103.

10. Doi L, Williams AJ, Frank J. How has child growth around adiposity rebound altered in Scotland since 1990 and what are the risk factors for weight gain using the Growing Up in Scotland birth cohort 1? *BMC Public Health* 2016; **16:** 1081.

11. Information Service Division (2018). Scottish birth record. http://www.ndc.scot.nhs.uk/Data-Dictionary/SMR-Datasets/Scottish-Birth-Record/

12. Cole TJ, Freeman JV, Preece MA. Body mass index reference curves for the UK, 1990. *Arch Dis Child* 1995; **73(1):** 25–29.

13. Freeman JV, Cole TJ, Chinn S, Jones PRM, White EM, Preece MA. Cross-sectional stature and weight reference curves for the UK 1990. *Arch Dis Child* 1995; **73(1):**17–24.

14. StataCorp. Stata statistical software: release 14. College Station: StataCorp LP; 2015.

15. O'Brien TE, Ray JG, Chan WS. Maternal body mass index and the risk of preeclampsia: A systematic overview. *Epidemiology* 2003; **14:** 368-374.

16. Shieh C, Cullen DL, Pike C, Pressler SJ. Intervention strategies for preventing excessive gestational weight gain: systematic review and meta-analysis. *Obesity Reviews* 2018; doi: 10.1111/obr.12691.

17. Thangaratinam S, Rogozinska E, Jolly K, Glinkowski S, Roseboom T, Tomlinson JW, Kunz R, Mol BW, Coomarasamy A, Khan KS. Effects of

For peer review only - http://bmjopen.bmj.com/site/about/guidelines.xhtml

interventions in pregnancy on maternal weight and obstetric outcomes: Meta-analysis of randomised evidence. *BMJ.* 2012; **344:** e2088.

18. Althabe F, Sosa C, Belizán JM, Gibbons L, Jacquerioz F, Bergel E. Cesarean section rates and maternal and neonatal mortality in low-, medium-, and high-income countries: an ecological study. *Birth* 2006; **33(4):** 270-277.
19. Rezaie M, Shahoe R, Shahghebi S. The effect of maternal body mass index on the delivery route in nulliparous women. *Journal of Public Health and Epidemiology* 2013; **5(8):** 346-350.
20. Health Improvement Scotland (2009). Pathways for maternity care. http://www.healthcareimprovementscotland.org/default.aspx?page=12536
21. Abhyankar P, Cheyne H, Maxwell M, McCourt C. A realist evaluation of a normal birth programme. *Evidence Based Midwifery* 2013; https://www.rcm.org.uk/learning-and-career/learning-and-research/ebm-articles/a-realist-evaluation-of-a-normal-birth.
22. Cheyne H, Abhyankar P, McCourt C. Empowering change: realist evaluation of a Scottish Government programme to support normal birth. *Midwifery* 2013; **29:** 1110–1121.

Funding

This work was funded by the SCPHRP core grant from the Medical Research Council (Grant Number MR/K023209/1) and the Chief Scientist Office of Scotland. AJW is supported by the European Centre for Environment and Human Health, University of Exeter. LM is supported by the Farr Institute @ Scotland, which is

For peer review only - http://bmjopen.bmj.com/site/about/guidelines.xhtml

supported by a 10-funder consortium: Arthritis Research UK, the British Heart Foundation, Cancer Research UK, the Economic and Social Research Council, the Engineering and Physical Sciences Research Council, the Medical Research Council, the National Institute of Health Research, the National Institute for Social Care and Health Research (Welsh Assembly Government), the Chief Scientist Office (Scottish Government Health Directorates), (MRC Grant No: MR/K007017/1). The funders played no role in the conceptualisation or realisation of the research and no role in the decision to submit it for publication.

Competing interest

The authors declare that they have no competing interests.

Data sharing

No additional data available.

Acknowledgements

The authors are grateful to the Electronic Data Research and Innovation Service of the Information Services Division, NHS National Services Scotland for providing the data used in this paper.

Authors' contributions

LD, AJW and JF conceived the original idea for the study and obtained the data. AJW led the statistical analysis with support from LD, LM and JF. LD wrote the first

For peer review only - http://bmjopen.bmj.com/site/about/guidelines.xhtml
For peer review only

draft of the paper and all authors revised successive drafts and approved the final manuscript.

For peer review only

For peer review only - http://bmjopen.bmj.com/site/about/guidelines.xhtml

Figure 1. Maternal weight status and neonatal outcomes analysis

For peer review only

For peer review only - http://bmjopen.bmj.com/site/about/guidelines.xhtml

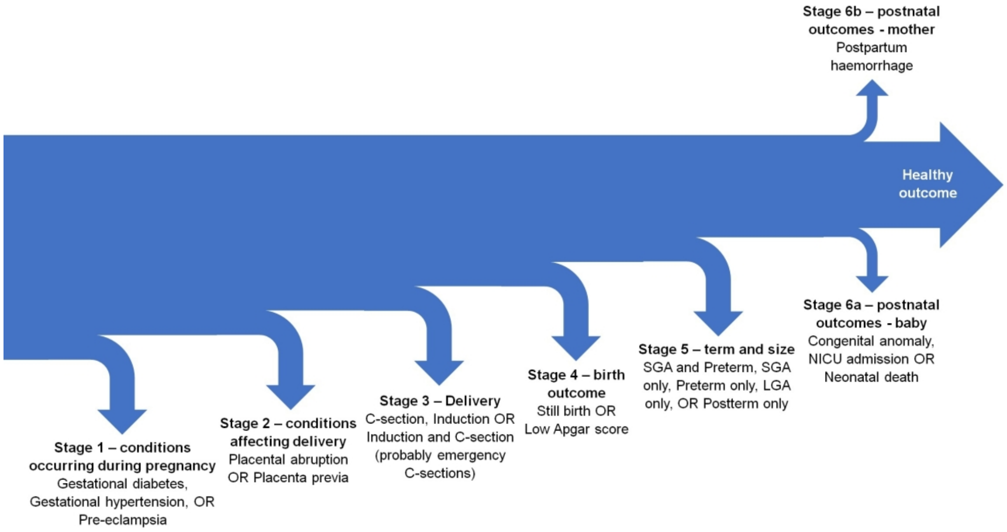

192x103mm (300 x 300 DPI)

BMJ Open

A cohort study of high maternal Body Mass Index and the risk of adverse pregnancy, delivery and neonatal outcomes in Scotland

Journal:	*BMJ Open*
Manuscript ID	bmjopen-2018-026168.R1
Article Type:	Original research
Date Submitted by the Author:	17-Jun-2019
Complete List of Authors:	Doi, Lawrence; University of Edinburgh, Scottish Collaboration for Public Health Research and Policy, School of Health in Social Science<br>Williams, Andrew James; University of Exeter<br>Marryat, Louise; University of Edinburgh, SCPHRP<br>Frank, John; University of Edinburgh, Scottish Collaboration for Public Health Research and Policy
**Primary Subject Heading**:	Obstetrics and gynaecology
Secondary Subject Heading:	Epidemiology, Paediatrics, Public health, Reproductive medicine, Health policy
Keywords:	OBSTETRICS, EPIDEMIOLOGY, PUBLIC HEALTH

SCHOLARONE™
Manuscripts

BMJ

I, the Submitting Author has the right to grant and does grant on behalf of all authors of the Work (as defined in the below author licence), an exclusive licence and/or a non-exclusive licence for contributions from authors who are: i) UK Crown employees; ii) where BMJ has agreed a CC-BY licence shall apply, and/or iii) in accordance with the terms applicable for US Federal Government officers or employees acting as part of their official duties; on a worldwide, perpetual, irrevocable, royalty-free basis to BMJ Publishing Group Ltd ("BMJ") its licensees and where the relevant Journal is co-owned by BMJ to the co-owners of the Journal, to publish the Work in this journal and any other BMJ products and to exploit all rights, as set out in our licence.

The Submitting Author accepts and understands that any supply made under these terms is made by BMJ to the Submitting Author unless you are acting as an employee on behalf of your employer or a postgraduate student of an affiliated institution which is paying any applicable article publishing charge ("APC") for Open Access articles. Where the Submitting Author wishes to make the Work available on an Open Access basis (and intends to pay the relevant APC), the terms of reuse of such Open Access shall be governed by a Creative Commons licence – details of these licences and which Creative Commons licence will apply to this Work are set out in our licence referred to above.

Other than as permitted in any relevant BMJ Author's Self Archiving Policies, I confirm this Work has not been accepted for publication elsewhere, is not being considered for publication elsewhere and does not duplicate material already published. I confirm all authors consent to publication of this Work and authorise the granting of this licence.

For peer review only

A cohort study of high maternal Body Mass Index and the risk of adverse pregnancy, delivery and neonatal outcomes in Scotland

Lawrence Doi[1]*, Andrew James Williams[2], Louise Marryat[3,4], John Frank[3,5]

*Correspondence: larry.doi@ed.ac.uk

[1]Scottish Collaboration for Public Health Research and Policy, School of Health in Social Science, University of Edinburgh, Doorway 6, Old Medical School, Teviot Place, EH8 9AG, UK.

[2]European Centre for Environment and Human Health, College of Medicine and Health, University of Exeter, Knowledge Spa, Royal Cornwall Hospital, Truro, Cornwall TR1 3HD, UK.

[3]Farr Institute at Scotland, University of Edinburgh, Nine, Edinburgh BioQuarter, 9 Little France Road, Edinburgh EH16 4UX, UK.

[4]Centre for Clinical Brain Sciences, University of Edinburgh, Royal Edinburgh Hospital, Kennedy Tower, Morningside Park, Edinburgh EH10 5HF, UK.

[5]Usher Institute of Population Health Sciences and Informatics, University of Edinburgh, Doorway 1, Old Medical School, Teviot Place, EH8 9AG, UK.

For peer review only

Abstract

Objective: To examine the association between high maternal weight status on pregnancy, delivery and neonatal outcomes.

Setting: Scotland

Participants: Data from 135,858 first time singleton deliveries in Scotland between 2008 and 2015 were used. Women with overweight and obesity were compared with women with normal weight. Associations between maternal body mass index and pregnancy and neonatal outcomes were evaluated in a staged fashion, according to the clinical timelines by which pregnancy, delivery and neonatal outcomes occur.

Outcome measures: Gestational diabetes, gestational hypertension, pre-eclampsia, placenta praevia, placental abruption, postpartum haemorrhage, induction of labour, elective and emergency caesarean sections, pre-term delivery, post-term delivery, low Apgar score, stillbirth, congenital anomalies, neonatal intensive care unit admission, neonatal death, small for gestational age and large for gestational age.

Results: In the multivariable models controlling for potential covariates, we found that compared with women with normal weight, the odds of the following outcomes were significantly increased for women with overweight and obesity [overweight adjusted odds ratio; 95% confidence interval, followed by the same for women with obesity]: gestational hypertension [1.62; 1.5-1.75], [2.61; 2.42-2.82] ; gestational diabetes [2.19; 1.91-2.52], [8.71; 7.74-9.81] ; pre-eclampsia [1.47; 1.33-1.63] [2.19; 1.98-2.42]]; labour induction [1.33; 1.27-1.39], [1.76; 1.67-1.86] ; emergency caesarean section [1.90; 1.78-2.02], [3.34; 3.12-3.57] and stillbirth [2.27; 1.20-4.29], [3.21; 1.59-6.48].

For peer review only - http://bmjopen.bmj.com/site/about/guidelines.xhtml

Conclusions: Women with overweight and obesity in Scotland are at greater risk of adverse pregnancy, delivery and neonatal outcomes. The risk of these conditions increases steadily with increasing body mass index. Health professionals should be empowered and trained to deliver promising dietary and lifestyle interventions to women at risk of overweight and obesity prior to conception, and control excessive weight gain in pregnancy.

Strengths and limitations of this study

- This study used a large, retrospectively accessed but cohort-structured, national database covering all major maternal and neonatal outcomes in Scotland over eight recent years.
- The staged analysis approach employed ensured an estimate of the precise impact of high maternal-weight status on each outcome.
- All women with BMI of 30 kg/m^2 or more were considered as having obesity; it is likely that differentiating morbid obesity or obesity class II and III from obesity would have generated more precise risk estimates.
- The completeness of the recording of BMI increased during the study period (2008 to 2015) from 69% to 98%. Using data from the earlier years when the BMI was missing more often might have biased the study sample if it was the case that BMI was not missing at random.

For peer review only - http://bmjopen.bmj.com/site/about/guidelines.xhtml

Introduction

The increasing global prevalence of overweight and obesity makes it more likely that a growing number of women with high body mass index (BMI) are becoming pregnant. High maternal BMI during pregnancy has adverse health implications for women (1,2) and predisposes the unborn child to adverse outcomes, including neonatal deaths, stillbirth, and admission to a neonatal unit (3,4,5). A recent international systematic review involving 38 studies found that even modest increases in maternal BMI were associated with increased risk of fetal death, stillbirth, and neonatal, perinatal, and infant death (6). Obesity in pregnancy can have health implications later in life for both mother and child. Among women this could lead to diabetes, heart disease and hypertension, whilst children are more prone to future obesity and hypertension (7). Wider service and economic consequences can also be seen including indirect burdens of illness and associated costs, in that women with high BMI also experience adverse birth outcomes such as neonatal death or stillbirth (8,9,10). Such individuals may require support from family and friends as well as bereavement support services within the community. There are also substantial healthcare cost implications for infants who may require medical attention. For example, a recent study examining infant health utilisation and costs on the NHS in the UK of infants born to women with overweight or obesity found that total mean additional resource cost for infants born to women who are overweight was £65.13, and £1138.11 for infants born to women who are obese (11).

Maternal weights are currently high in Scotland: a recent study reported that 31.5% of mothers were overweight and a further 23.6% were affected by obesity (12). Another study examining the impact of maternal BMI on clinical complications, inpatient

For peer review only - http://bmjopen.bmj.com/site/about/guidelines.xhtml

For peer review only

admissions, and additional short-term costs to the NHS in Scotland revealed that maternal BMI influences maternal and neonatal morbidity, the number and duration of maternal and neonatal admissions, and health service costs (13). However, no recent study in Scotland has investigated the effect of high maternal BMI on the risk of pregnancy and neonatal outcomes. The aim of this study was to examine the associations between high maternal BMI during pregnancy and perinatal risks, including adverse neonatal outcomes. Understanding of these associations can highlight areas where prevention strategies could be targeted.

Methods

Study population and data sources

This retrospective cohort study used data from 135,858 first time mothers who gave birth to only one child in Scotland between January 2008 and December 2015. The women and infants were identified within three electronic medical record databases: the Scottish Morbidity Record (SMR) 01 and 02 and Scottish Birth Record (SBR). SMR01 is generated for patients receiving inpatient or day care in the General or Acute specialties, whilst SMR02 is generated for patients receiving inpatient or day care in the Obstetric Specialties. The SBR records all of a baby's neonatal care in Scotland. The outcome variables are recorded in these databases according the World Health Organization's International Classification of Diseases, Tenth Revision (ICD-10). Further description of the content of these databases is available (14) and in the web-appendix. The study was designed as a clinical audit so did not require approval from a Research Ethics Committee. However, approval was obtained from

For peer review only - http://bmjopen.bmj.com/site/about/guidelines.xhtml

the Public Benefit and Privacy Panel via the national Electronic Data Research and Innovation Service to use the anonymised data collected by these registries.

Patient and public involvement

Patients were not involved in the design, analysis and interpretation of this study.

Exposure variable

More than 80% of pregnant women in Scotland present themselves for antenatal care during the first trimester of their pregnancy. Height and weight are measured by the midwife at the first antenatal visit, typically before 12 weeks of pregnancy. Body mass index was calculated using the formula weight (kg)/height (m^2). BMI completeness was 69% in 2008 but this increased gradually to 87% in 2011 when recording of weight and height became mandatory. By 2015 BMI completeness was 98%. Women were categorised into three BMI groups as described in Table 1.

Table 1. Adult BMI definitions used

Variable	Category	BMI Definition
Maternal weight status	Normal	<25 kg/m^2
	Overweight	≥25 kg/m^2 to <30 kg/m^2
	Obesity	≥30 kg/m^2

Outcomes

Outcome measures included were maternal or pregnancy complications such as gestational diabetes, gestational hypertension, pre-eclampsia (high blood pressure and protein in urine), placenta praevia (when a baby's placenta partially or totally covers the mother's cervix), placental abruption (when the placenta separates early from the uterus before childbirth) and postpartum haemorrhage (loss of more than

For peer review only - http://bmjopen.bmj.com/site/about/guidelines.xhtml

500 ml or 1,000 ml of blood within the first 24 hours following childbirth). Delivery complications studied were induction of labour, caesarean delivery (includes elective and emergency caesarean sections), pre-term delivery (defined as less than 37 weeks of gestation), post-term delivery (more than 42 weeks of gestation), small for gestational age (SGA), and large for gestational age (LGA). SGA were infants with birthweight of ≤10th percentile for gestational age according to UK1990 growth reference curve (15,16), and those with LGA were infants with birth weight ≥90th percentile.

Neonatal outcomes studied were: low Apgar score (less than “7” at 5 minutes), stillbirth, congenital anomalies, neonatal intensive care unit (NICU) admission, neonatal death.

Covariates

Maternal age at delivery, smoking during pregnancy and Carstairs 2001 quintiles for Scotland for the postcode sector of the mother’s residence at birth, were considered as potentially confounding variables and were included as covariates in the adjusted analyses. Table 2 describes the covariates used in this study by maternal weight status among singleton (a pregnancy with one fetus as opposed to twins or multiples) first-time pregnancies. The data in Table 2 show the numbers of women who had data on age, deprivation and maternal smoking. This means that the total number of women is slightly larger than the number in stage one of our analysis, as there were women missing hypertension, diabetes or pre-eclampsia data.

For peer review only - http://bmjopen.bmj.com/site/about/guidelines.xhtml

Table 2. Maternal characteristics among normal weight, overweight and obese women+ (singleton, first pregnancies)

	Normal* N = 73,130		Overweight* N = 36,992		Obese* N = 25,738	
	**N**	**%**	**N**	**%**	**N**	**%**
**Maternal age (y)**						
20-24	19,874	27.2	9,368	25.3	7,016	27.3
25-29	24,443	33.4	12,174	32.9	8,475	32.9
30-34	20,880	28.6	10,522	28.4	6,922	26.9
35-39	7,933	10.9	4,928	13.3	3,325	12.9
**Carstairs 2001 quintiles for Scotland**						
Q1 (Least deprived)	14,695	20.1	6,785	18.3	3,870	15.0
Q2	13,820	18.9	6,851	18.5	4,639	18.0
Q3	14,581	19.9	7,549	20.4	5,117	19.9
Q4	15,338	21.0	7,994	21.6	6,079	23.6
Q5 (Most deprived)	14,696	20.1	7,813	21.1	6,033	23.4
**Maternal smoking in pregnancy**						
No	62,439	85.4	31,806	86.0	21,604	83.9
Yes	10,691	14.6	5,186	14.0	4,134	16.1

*Maternal weight status at first antenatal visit
+Figures show women who had complete data on age, deprivation and maternal smoking

Data analysis

Using Stata 14 (17), logistic regression was used to calculate odds ratios (ORs). BMI groups with overweight and obesity were compared with the normal BMI group (the reference population). A confidence interval (CI) of 95% was produced for all ORs. The analysis of the outcomes proceeded in a staged fashion according to the clinical timelines by which pregnancy, delivery and neonatal outcomes occur (Figure 1).

Firstly, all conditions occurring during pregnancy such as gestational hypertension, gestational diabetes and pre-eclampsia, which may also be a risk factor for later outcomes, were compared with a ‘healthy’ group who did not have any of these conditions. The second stage of the analysis considered only the group with none of the stage one conditions. Placental abruption, placenta praevia, size for gestational age and whether labour began before, at or after term were considered within this stage because the risk of both conditions is associated with one or more of the stage

For peer review only - http://bmjopen.bmj.com/site/about/guidelines.xhtml

one conditions. Therefore, by limiting the dataset to those without any of the stage one conditions, a better estimate of the impact of maternal weight status is established. This approach means the sample size reduces steadily as one moves through the process. The stages used for the analyses are outlined in Figure 1. In the final stage there are two sub-stages because postpartum haemorrhage is a maternal outcome and we wanted to treat this outcome independent of NICU admission, congenital anomaly and neonatal death.

<Figure 1>

Results

Within our study population 53.8 % of pregnant women were categorised as normal weight, 27.3% overweight and 18.9% were affected by obesity. The socio-demographic characteristics of the women in the three BMI categories are presented in Table 2. Maternal smoking prevalence was slightly higher among women with obesity than in women with normal weight or overweight. Among the women who were overweight, 21.1% were from the most deprived and 18.3% were from the least deprived group. However, the difference in social deprivation was more marked within women with obesity. Among this group, 23.4% were from the most deprived group whilst 15.0% were from the least deprived group.

Table 3 shows odd ratios for pregnancy and delivery complications, as well as neonatal outcomes, among women who were overweight or obese, and with singleton first-time births. The risk of the three conditions occurring during pregnancy increased steadily with increasing BMI. For example, compared to the normal BMI group, the risk of gestational diabetes was 2.19 (95% CI: 1.91-2.52) but among

For peer review only - http://bmjopen.bmj.com/site/about/guidelines.xhtml

women who were obese the risk was increased to 8.71 (95% CI: 8.71 – 9.81).

Relative to women who were of normal weight, the adjusted OR of pre-eclampsia for women who were overweight was 1.47 (95% CI: 1.33-1.63) and 2.19 (95% CI: 1.98-2.42) for women who were obese.

For peer review only

For peer review only - http://bmjopen.bmj.com/site/about/guidelines.xhtml

Table 3. Pregnancy and delivery complications and neonatal outcomes among normal, overweight and obese singleton women

	Total	Normal		Overweight			Obese		
	sample	N (%)	Cases (%)	N (%)	Cases (%)	Adjusted OR* (95% CI)	N (%)	Cases (%)	Adjusted OR* (95% CI)
**Stage 1: Conditions occurring during pregnancy**									
Gestational hypertension	128,977	70,276 (54.5)	1,554 (2.2)	35,152 (27.3)	1,241 (3.5)	1.62 (1.50, 1.75)	23,549 (18.3)	1,280 (5.4)	2.61 (2.42, 2.82)
Gestational diabetes	126.779	69,099 (54.5)	377 (0.6)	34,329 (27.1)	418 (1.2)	2.19 (1.91, 2.52)	23,351 (18.4)	1,082 (4.6)	8.71 (7.74, 9.81)
Pre-eclampsia	127,155	69,644 (54.8)	922 (1.3)	34.586 (27.2)	675 (2.0)	1.47 (1.33, 1.63)	22,925 (18.0)	656 (2.9)	2.19 (1.98, 2.42)
**Stage 2: Conditions affecting delivery**									
Placenta praevia	74,076	42,021 (56.7)	102 (0.2)	19,817 (26.8)	64 (0.3)	1.28 (0.93, 1.75)	12,238 (16.5)	29 (0.2)	0.96 (0.64, 1.46)
Placental abruption	74,081	42,040 (56.8)	121 (0.3)	19,800 (26.7)	47 (0.2)	0.80 (0.57, 1.13)	12,241 (16.5)	32 (0.3)	0.87 (0.59, 1.29)
Small for gestational age (SGA)	83,503	47,881 (57.3)	5,962 (12.5)	22,017 (26.4)	2,264 (10.3)	0.80 (0.76, 0.84)	13,605 (16.3)	1,396 (10.3)	0.77 (0.72, 0.82)
Pre-term	79,013	44,831 (56.7)	2,912 (6.5)	21,057 (26.7)	1,304 (6.2)	0.94 (0.88, 1.00)	13,125 (16.6)	916 (7.0)	1.06 (0.98, 1.14)
SGA and pre-term	74,510	42,260 (56.7)	341 (0.8)	19,915 (26.7)	162 (0.8)	0.99 (0.82, 1.20)	12,335 (16.6)	126 (1.0)	1.19 (0.96, 1.46)
Post-term	73,947	41,950 (56.7)	31 (0.1)	19,775 (26.7)	22 (0.1)	1.53 (0.88, 2.64)	12,222 (16.5)	13 (0.1)	1.52 (0.79, 2.92)
Large for gestational age	107,189	58,373 (54.5)	16,454 (28.2)	29,480 (27.5)	9,727 (33.0)	1.26 (1.22, 1.30)	19,336 (18.0)	7,127 (36.9)	1.52 (1.47, 1.58)
**Stage 3: Delivery**									
Induction of labour	55,265	33,032 (59.8)	7,633 (23.1)	14,222 (25.7)	4,074 (28.7)	1.33 (1.27, 1.39)	8,011 (14.5)	2,791 (34.8)	1.76 (1.67, 1.86)
Caesarean section	51,963	31,235 (60.1)	5,836 (18.7)	13,348 (25.7)	3,200 (24.0)	1.34 (1.28, 1.41)	7,380 (14.2)	2,160 (29.3)	1.80 (1.70, 1.91)
Emergency caesarean section	47,620	28,178 (59.2)	2,779 (9.9)	12,303 (25.8)	2,155 (17.5)	1.90 (1.78, 2.02)	7,139 (15.0)	1,919 (26.9)	3.34 (3.12, 3.57)
**Stage 4: Birth outcome**									
Apgar score	40,716	25,379 (62.3)	4,856 (19.1)	10,130 (24.9)	1,982 (19.6)	1.02 (0.96, 1.08)	5,207 (12.8)	983 (18.9)	0.95 (0.88, 1.03)
Stillbirth	32,946	20,543 (62.4)	20 (0.1)	8,166 (24.8)	18 (0.2)	2.27 (1.20, 4.29)	4,237 (12.9)	13 (0.3)	3.21 (1.59, 6.48)
**Stage 5: Postnatal outcomes (infant and maternal)**									
NICU admission	32,722	20,423 (62.4)	719 (3.5)	8,101 (24.8)	322 (4.0)	1.13 (0.98, 1.29)	4,198 (12.8)	205 (4.9)	1.39 (1.18, 1.63)
Neonatal death	-	19,707 (62.6)	3 (<0.1)	7,779 (24.7)	0 (0.0)	-	3,993 (12.7)	0 (0.0)	-
Congenital anomaly	16,951	10,609 (62.6)	29 (0.3)	4,177 (24.6)	16 (0.4)	1.38 (0.75, 2.54)	2,165 (12.8)	9 (0.4)	1.42 (0.67, 3.01)
Postpartum haemorrhage	32,895	20,523 (62.4)	3,947 (19.2)	8,148 (24.8)	1,565 (19.2)	0.99 (0.93, 1.06)	4,224 (12.8)	879 (20.8)	1.12 (1.03, 1.21)

OR = Odd Ratio; CI = Confidence Interval; NICU = Neonatal Intensive Care Unit

*Adjusted for maternal age, deprivation and smoking in pregnancy

For peer review only - http://bmjopen.bmj.com/site/about/guidelines.xhtml

Regarding conditions affecting delivery, the odds ratio of placenta praevia were not statistically significant for both women who were overweight (OR 1.28, 95%CI: 0.93-1.75) and obese (OR 0.96, 95%CI: 0.64-1.46) compared with women with normal weight. The odds of experiencing placental abruption were also not significantly different among the different BMI categories.

In contrast with the normal BMI group, births to women who were overweight and obese were associated with decreased risk of small-for-gestational age, ORs 0.80 (95% CI: 0.76-0.84) and 0.77 (95% CI: 0.72-0.82) respectively. However, the prevalence of large-for-gestational age increased among women with overweight, OR of 1.26 (95%CI: 1.22-1.30) and women with obesity, OR 1.52 (95% CI: 1.47-1.58) compared with women of normal weight.

The chance of induction of labour and caesarean section, either elective or emergency increased with increasing BMI. Women who were overweight had odds of 1.34 (95% CI: 1.28-1.41) higher for having an elective Caesarean section and higher odds (OR 1.90, 95% CI: 1.78-2.02) for undergoing emergency Caesarean section, compared with women of normal weight. The corresponding ORs for women with obesity were 1.80 (95% CI: 1.70-1.91) and 3.34 (95% CI: 3.12-3.57).

In terms of birth outcomes, the risk of stillbirth was not significantly higher for both women who were overweight (OR 2.27, 95%CI: 1.20-4.29) and obese (OR 3.21, 95%CI: 1.59-6.48) relative to normal weight women. Being overweight (OR 1.02 , 95% CI: 0.96-1.08) or obese (OR 0.95, 95% CI: 0.88-1.03) did not significantly affect the risk of low Apgar score, likely because women with obesity and

For peer review only - http://bmjopen.bmj.com/site/about/guidelines.xhtml
For peer review only

overweight may receive increased monitoring, which means issues can be identified and managed earlier, to reduce any fetal distress in labour.

There were no statistically significant differences in ORs among women who were overweight when postnatal outcomes were considered. However, among women who were obese, the odds of a baby being admitted to NICU was 1.39 (95% CI: 1.18-1.63) in contrast to women with normal weight. The odds of experiencing postpartum haemorrhage were also statistically significant for women who were obese (OR 1.12, 95%CI: 1.03-1.21), compared with women of normal weight.

Discussion

In this large, retrospective cohort study using a staged approach to analysis, we found that overweight or obesity during pregnancy increased the risk of several adverse pregnancy and delivery complications, and as well as poor neonatal outcomes. Some earlier studies have also found similar findings. Aside from obesity, we also examined overweight because in most populations, a greater number of women are overweight rather than obese, so it is important to also understand the impact of overweight on pregnancy and neonatal outcomes.

In terms of the association between high maternal BMI and conditions that occur during pregnancy, we found that the risk of all the conditions considered (gestational hypertension, gestational diabetes and pre-eclampsia) increased steadily with increasing BMI, which is in line with similar studies (2,3,4). A study compared women ofnormal weight to women who were morbidly obese (BMI greater than 40), and also

For peer review only

found that there was an increased risk of pre-eclampsia (OR 4.82; 95% CI: 4.04-5.74) (3). Our study also found that, aside from heightened pre-eclampsia risk for women with obesity, being overweight was also significantly associated with this outcome, albeit to a lesser degree. A meta-analysis of the association between maternal BMI and the risk of pre-eclampsia showed that the risk doubled with each 5-7 kg/m^2 increase in pre-pregnancy BMI (18). It is evident that the risk of pre-eclampsia increases with the degree of weight gain; therefore preventative strategies should be focussed on getting women, especially those already overweight, to reduce weight prior to conception. Weight loss in pregnancy requires careful management in order to avoid unintended consequences (19). Nevertheless, women often engage with health professionals during pregnancy; therefore dietary and lifestyle interventions such as physical activity, which have been shown by reviews and meta-analyses (19,20) to reduce gestational weight gain and improve outcomes for both mother and baby, could be provided to them.

Generally, rates of Caesarean delivery have increased significantly across many developed countries in recent years (21). Our study found that women with overweight and obesity showed an increased risk of Caesarean delivery (both elective and emergency) compared to normal-weight women, but we note that the overall frequency of Caesarean delivery across our obstetric population seems quite high, compared to a previous Swedish study (3). Possible reasons could be that Caesarean delivery is probably less risky now, due to advances in medical science, which facilitate accurate monitoring of the progress of labour and fetal intra-partum condition (22). Nevertheless, this is quite concerning for Scotland, which has invested in programmes aimed at promoting natural birth such as Keeping Childbirth

For peer review only - http://bmjopen.bmj.com/site/about/guidelines.xhtml

Natural and Dynamic (KCND). The KCND is a maternity care programme introduced by the Scottish Government with the aim of maximising opportunities for women to have as natural a birth experience as possible, reduce unnecessary interventions in low-risk pregnancy and childbirth, and to provide women-centred care (23,24,25).

We found that stillbirth was significantly associated with both overweight and obesity. This is congruent with the Aune et al. (6) systematic review and meta-analysis. However, Scott-Pillai et al. (4) observed that women who were overweight were not at increased risk of stillbirth, neither were those with class I (BMI 30.00-34.99) and II (BMI 35.00-39.99) obesity. Women in obesity class III (BMI ≥40) were found to be at increased risk of stillbirth. Cedergren (3) also found that stillbirth was significantly associated with both obesity and morbid obesity. Our study found that within the cohort there were no fetal deaths to first time mothers who were overweight or obese and who gave birth to only one child. This was in contrast to a previous study (3). Since Caesarean deliveries were more frequent among our obstetric study population, it is possible that health professionals in Scotland are probably intervening early with regards to problems in labour among women with overweight or obesity, in order to reduce fetal distress, and its worst outcome. This early intervention in labour to reduce fetal distress could also possibly explain our findings that overweight or obesity was not significantly associated with decreased risk of low Apgar score.

Adiposity increases risk of large-for-gestational age and macrosomia; in this study, we found that births to women with overweight and obesity were associated with an increased risk of large-for-gestational age infants compared with women of normal

For peer review only - http://bmjopen.bmj.com/site/about/guidelines.xhtml

weight. Excess weight in pregnancy may shift the entire birth weight distribution upwards, perhaps through hormonal mechanisms that operate at lower levels than in full-blown cases of macrosomia in infants of diabetic mothers.

Strengths and limitations

This study used a large, retrospectively accessed but cohort-structured, national database covering all major maternal and neonatal outcomes. The staged analysis approach we employed ensured a more precise estimate of the impact of high maternal-weight status on each outcome. We restricted the analysis to only single births and first pregnancies to ensure that the births in the sample are relatively independent. The dataset we used combined underweight and normal weight women as normal BMI group. Using this as reference group might have strengthened the association between high maternal BMI and the pregnancy and neonatal outcomes considered. However, only a very small number of women are underweight during pregnancy in Scotland in recent years. Also, some studies differentiate between different obesity categories, but in this study all women with BMI of 30 or more were considered as having obesity. It is likely that differentiating morbid obesity, or obesity class II and III from women with obesity, would have generated additional insight. The completeness of the recording of BMI increased during the study period (2008 to 2015) from 69% to 98%. Using data from the earlier years, when the BMI was missing more often, might have biased the study sample if BMI was not missing at random. The study included control for a limited set of confounders. All these limitations are common in studies like this one, utilising routinely collected administrative records, but such studies can be undertaken inexpensively and quickly compered to studies collecting such data first hand.

For peer review only - http://bmjopen.bmj.com/site/about/guidelines.xhtml

Conclusion

This study has shown that women who were overweight and obese in Scotland are at greater risk of several pregnancy and delivery complications including gestational hypertension, gestational diabetes, pre-eclampsia, labour induction and Caesarean delivery. The risk of these conditions increases steadily with increasing BMI. Women with obesity also had three-fold risk increase for stillbirth. Health professionals should be empowered and trained to deliver promising dietary and lifestyle interventions to women at risk of overweight and obesity prior to conception, and control excessive weight gain in pregnancy.

References

1. Rooney BL, Schauberger CW. Excess pregnancy weight gain and long-term obesity: one decade later. *Obstet Gynecol* 2002; **100(2):** 245-252.
2. Kumari P, Gupta M, Kahlon P, Malviya S. Association between high maternal body mass index and fetomaternal Outcome. *J Obes Metab Res* 2014; **1:** 143-148.
3. Cedergren MI. Maternal morbid obesity and the risk of adverse pregnancy outcome. *Obstetricians and Gynecologists* 2004; **103:** 219-224.
4. Scott-Pillai R, Spence D, Cardwell C, Hunter A, Holmes V. The impact of body mass index on maternal and neonatal outcomes: a retrospective study in a UK obstetric population, 2004–2011. *BJOG* 2013; **120:** 932–939.
5. Rahman MM, Abe SK, Kanda M, Narita S, Rahman MS, Bilano V, Ota E, Gilmour S, Shibuya K. Maternal body mass index and the risk of birth and maternal health outcomes in low- and middle-income countries: a systematic review and meta-analysis. *Obesity Reviews* 2015; **16:** 758–770.

For peer review only - http://bmjopen.bmj.com/site/about/guidelines.xhtml

6. Aune D, Saugstad OD, Henriksen T, Tonstad S. Maternal body mass index and the risk of fetal death, stillbirth, and infant death: a systematic review and meta-analysis. *JAMA* 2014; **311**(15):1536-46.
7. Leddy MA, Power ML, Schulkin J. The impact of maternal obesity on maternal and fetal health. *Rev Obstet Gynecol*. 2008;**1**(4):170–178.
8. Heslehurt N, Lang R, Rankin J, Wilkinson J, Summerbell CD. Obesity in pregnancy: a study of the impact of maternal obesity on NHS maternity services. *BJOG* 2007; **114(3):** 334-342.
9. Furness PJ, McSeveny K, Arden MA, Garland C, Dearden AM, Soltani H. Maternal obesity support services: a qualitative study of the perspectives of women and midwives. *BMC Pregnancy and Childbirth* 2011; **11:** 69.
10. Lenoir-Wijnkoop I, van der Beek EM, J Garssen, Nuijten MJC, Uauy RD. Health economic modeling to assess short-term costs of maternal overweight, gestational diabetes, and related macrosomia – a pilot evaluation. *Front Pharmacol* 2015; **6:**103. doi: 10.3389/fphar.2015.00103.
11. Morgan KL, Rahman MA, Hill RA, et al. Obesity in pregnancy: infant health service utilisation and costs on the NHS. *BMJ Open* 2015;5:e008357. doi:10.1136/bmjopen-2015- 008357.
12. Doi L, Williams AJ, Frank J. How has child growth around adiposity rebound altered in Scotland since 1990 and what are the risk factors for weight gain using the Growing Up in Scotland birth cohort 1? *BMC Public Health* 2016; **16:** 1081.
13. Denison FC, Norwood P, Bhattacharya S, Duffy A, Mahmood T, Morris C, Raja EA, Norman JE, Lee AJ, Scotland G. Association between maternal

For peer review only - http://bmjopen.bmj.com/site/about/guidelines.xhtml

body mass index during pregnancy, short-term morbidity, and increased health service costs: a population-based study. *BJOG* 2014; **121**:72–82.

14. Information Service Division (2018). Scottish birth record. http://www.ndc.scot.nhs.uk/Data-Dictionary/SMR-Datasets/Scottish-Birth-Record/
15. Cole TJ, Freeman JV, Preece MA. Body mass index reference curves for the UK, 1990. *Arch Dis Child* 1995; **73(1):** 25–29.
16. Freeman JV, Cole TJ, Chinn S, Jones PRM, White EM, Preece MA. Cross-sectional stature and weight reference curves for the UK 1990. *Arch Dis Child* 1995; **73(1):**17–24.
17. StataCorp. Stata statistical software: release 14. College Station: StataCorp LP; 2015.
18. O'Brien TE, Ray JG, Chan WS. Maternal body mass index and the risk of preeclampsia: A systematic overview. *Epidemiology* 2003; **14:** 368-374.
19. Shieh C, Cullen DL, Pike C, Pressler SJ. Intervention strategies for preventing excessive gestational weight gain: systematic review and meta-analysis. *Obesity Reviews* 2018; doi: 10.1111/obr.12691.
20. Thangaratinam S, Rogozinska E, Jolly K, Glinkowski S, Roseboom T, Tomlinson JW, Kunz R, Mol BW, Coomarasamy A, Khan KS. Effects of interventions in pregnancy on maternal weight and obstetric outcomes: Meta-analysis of randomised evidence. *BMJ.* 2012; **344:** e2088.
21. Althabe F, Sosa C, Belizán JM, Gibbons L, Jacquerioz F, Bergel E. Cesarean section rates and maternal and neonatal mortality in low-, medium-, and high-income countries: an ecological study. *Birth* 2006; **33(4):** 270-277.

For peer review only - http://bmjopen.bmj.com/site/about/guidelines.xhtml

22. Rezaie M, Shahoe R, Shahghebi S. The effect of maternal body mass index on the delivery route in nulliparous women. *Journal of Public Health and Epidemiology* 2013; **5(8):** 346-350.

23. Health Improvement Scotland (2009). Pathways for maternity care. http://www.healthcareimprovementscotland.org/default.aspx?page=12536

24. Abhyankar P, Cheyne H, Maxwell M, McCourt C. A realist evaluation of a normal birth programme. *Evidence Based Midwifery* 2013; https://www.rcm.org.uk/learning-and-career/learning-and-research/ebm-articles/a-realist-evaluation-of-a-normal-birth.

25. Cheyne H, Abhyankar P, McCourt C. Empowering change: realist evaluation of a Scottish Government programme to support normal birth. *Midwifery* 2013; **29:** 1110–1121.

For peer review only

Funding

This work was funded by the SCPHRP core grant from the Medical Research Council (Grant Number MR/K023209/1) and the Chief Scientist Office of Scotland. AJW is supported by the European Centre for Environment and Human Health, University of Exeter. LM is supported by the Farr Institute @ Scotland, which is supported by a 10-funder consortium: Arthritis Research UK, the British Heart Foundation, Cancer Research UK, the Economic and Social Research Council, the Engineering and Physical Sciences Research Council, the Medical Research Council, the National Institute of Health Research, the National Institute for Social

For peer review only - http://bmjopen.bmj.com/site/about/guidelines.xhtml

Care and Health Research (Welsh Assembly Government), the Chief Scientist Office (Scottish Government Health Directorates), (MRC Grant No: MR/K007017/1). The funders played no role in the conceptualisation or realisation of the research and no role in the decision to submit it for publication.

Competing interest

The authors declare that they have no competing interests.

Data sharing

No additional data available.

Acknowledgements

The authors are grateful to the Electronic Data Research and Innovation Service of the Information Services Division, NHS National Services Scotland for providing the data used in this paper.

Authors' contributions

LD, AJW and JF conceived the original idea for the study and obtained the data. AJW led the statistical analysis with support from LD, LM and JF. LD wrote the first draft of the paper and all authors revised successive drafts and approved the final manuscript.

For peer review only

Figure 1. Maternal weight status and neonatal outcomes analysis

For peer review only

For peer review only - http://bmjopen.bmj.com/site/about/guidelines.xhtml

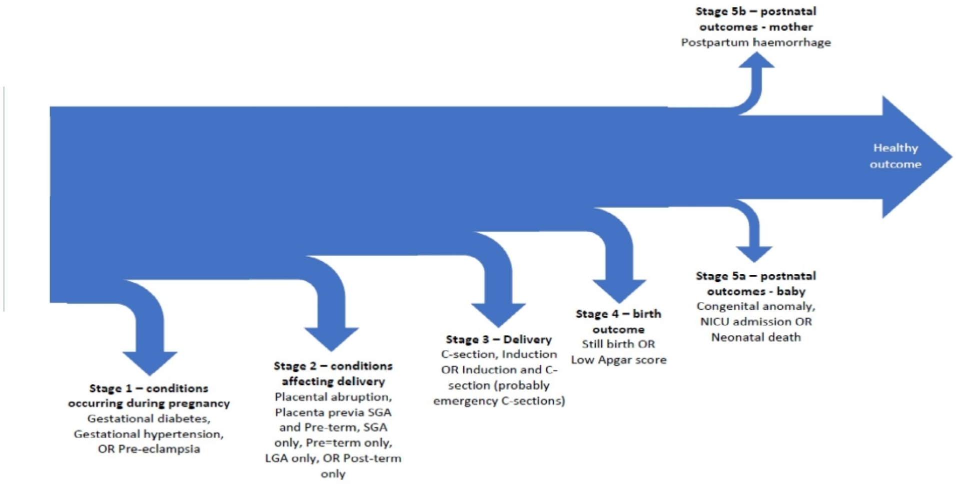

Maternal weight status and neonatal outcomes analysis

192x103mm (300 x 300 DPI)

Web-appendix: Description of data fields used in the study

Variable Name	Database Source	Variable Description/Values	Notes
Mother_ID	N/A - study specific	Unique mother identifier	1516-0613/' followed by an anonymous identifier
Baby_ID	N/A - study specific	Unique baby identifier	Mother_ID followed by delivery sequence number followed by a baby sequence number. The baby sequence number for multiple babies from same delivery not necessarily in correct order due to missing CHI numbers.
Delivery_Seq_No	N/A - study specific	Delivery sequence number of mother	
Gest_Diabetes	SMR02	**1:** Yes, Gestational diabetes (diagnosed during this pregnancy)<br>**0:** Yes, Pre-existing diabetes (diagnosed before pregnancy) & No (no diabetes during this pregnancy)<br>**Missing:** Yes, Time of diagnosis unknown & Not Known	http://www.ndc.scot.nhs.uk/Dictionary-A-Z/Definitions/index.asp?Search=D&ID=214&Title=Diabetes
Gest_Hypertension	SMR02/SMR01	**1:** ICD 10 code O13.X<br>**0:** All other codes	Flagged codes cover gestational hypertension
Pre_Eclampsia	SMR02/SMR01	**1:** ICD 10 code O14._<br>**0:** All other codes	Flagged codes cover pre-eclampsia
Placental_Abruption	SMR02/SMR01	**1:** ICD 10 code O45._<br>**0:** All other codes	Flagged codes cover placental abruption
Placental_Praevia	SMR02/SMR01	**1:** ICD 10 code O44._<br>**0:** All other codes	Flagged codes cover placenta praevia

For peer review only

Postpartum_Haemorrhage	SMR02/SMR01	**1:** ICD 10 code O72._<br>**0:** All other codes	Flagged codes cover postpartum haemorrhage
Caesarean_Delivery	SMR02	**1:** Elective (planned) caesarean section & Emergency and unspecified caesarean section<br>**0:** All other codes	http://www.ndc.scot.nhs.uk/Dictionary-A-Z/Definitions/index.asp?Search=M&ID=322&Title=Mode of Delivery - Babies 1 to 3
Labour_Induction	SMR02	**1:** 1-8 - Induction of labour codes<br>**0:** 0, None<br>**Missing:** 9, Not known	http://www.ndc.scot.nhs.uk/Dictionary-A-Z/Definitions/index.asp?Search=I&ID=295&Title=Induction of Labour
SGA	SMR02	**1:** Birthweight ≤10th percentile<br>**0:** Birthweight >10th percentile	Small for gestational age flag
LGA	SMR02	**1:** Birthweight ≥90th percentile<br>**0:** Birthweight <90th percentile	Large for gestational age flag
Preterm_Delivery	SMR02	**1:** Estimated gestation < 37 weeks<br>**0:** Estimated gestation ≥ 37 weeks and ≤ 42 weeks<br>**Missing:** otherwise	http://www.ndc.scot.nhs.uk/Dictionary-A-Z/Definitions/index.asp?Search=E&ID=242&Title=Estimated Gestation
Postterm_Delivery	SMR02	**1:** Estimated gestation > 42 weeks<br>**0:** Estimated gestation ≥ 37 weeks and ≤ 42 weeks<br>**Missing:** otherwise	http://www.ndc.scot.nhs.uk/Dictionary-A-Z/Definitions/index.asp?Search=E&ID=242&Title=Estimated Gestation
Apgar_Score	SMR02	**1:** Apgar score at 5 mins < 7<br>**0:** Apgar score at 5 mins ≥ 7	http://www.ndc.scot.nhs.uk/Dictionary-A-Z/Definitions/index.asp?Search=A&ID=88&Title=Apgar Score - Babies 1 to 3
Stillbirth	SMR02	**1:** Stillbirth<br>**0:** Livebirth<br>**Missing:** otherwise	http://www.ndc.scot.nhs.uk/Dictionary-A-Z/Definitions/index.asp?Search=O&ID=372&Title=Outcome of Pregnancy - Babies 1 to 3

For peer review only

Congenital_anomaly	SBR	**1:** Acute life threatening; Non-life threatening & Yes<br>**0:** None<br>**Missing:** Suspected & Not known	Note there is a lack of completeness until 2010, could be poor quality as not quality checked. Bias in recording levels - e.g. more highly recorded in Glasgow HB than Lothian HB.
NICU_admission	SMR02	**1:** Admitted - for up to 48 hours & Admitted - for more than 48 hours<br>**0:** Not admitted<br>**Missing:** Not known	http://www.ndc.scot.nhs.uk/Dictionary-A-Z/Definitions/index.asp?Search=N&ID=326&Title=Neonatal Indicator - Babies 1 to 3
Neonatal_death	SMR02	**1:** Livebirth dying within the first 6 days (early neonatal death) & Livebirth dying on or after the 7th completed day but before the 28th day (late neonatal death)<br>**0:** Livebirth<br>**Missing:** Otherwise	http://www.ndc.scot.nhs.uk/Dictionary-A-Z/Definitions/index.asp?Search=O&ID=372&Title=Outcome of Pregnancy - Babies 1 to 3
Maternal_Obesity	SMR02	**1:** Obese status<br>**0:** Overweight, Healthy or underweight status	Adults with BMI ≥ 30 classed as obese. BMI of girls aged 2 - 19 years old standardized using UK1990 growth reference values and z-score ≥ 6/3 (98th centile) classed as obese.
Maternal_Overweight_Obesity	SMR02	**1:** Overweight or Obese status<br>**0:** Healthy or underweight status	Adults with BMI ≥ 25 classed as overweight or obese. BMI of girls aged 2 - 19 years old standardized using UK1990 growth reference values and z-score ≥ 4/3 (91st centile) classed as overweight or obese.
Age	SMR02	Age of mother at delivery (in years)	
Parity	SMR02	Total number of previous pregnancies	http://www.ndc.scot.nhs.uk/Dictionary-A-Z/Definitions/index.asp?Search=P&ID=409&Title=Previous Pregnancies
Deprivation	SMR02	Carstairs 2001 quintiles for Scotland	1=least deprived; 5=most deprived

Smoking_Status	SMR02	**1:** Yes<br>**0:** No<br>**Missing:** Not known	http://www.ndc.scot.nhs.uk/Dictionary-A-Z/Definitions/index.asp?Search=S&ID=456&Title=Smoker During Pregnancy
Multiple_births	SMR02	**1:** More than one birth this pregnancy<br>**0:** Single birth	http://www.ndc.scot.nhs.uk/Dictionary-A-Z/Definitions/index.asp?Search=N&ID=349&Title=Number of Births this Pregnancy
Multiple_births_in_NRS	NRS Births	**1:** Multiple babies found for this mother's delivery in NRS Births	Multiple babies recorded in NRS Births but only a single baby recorded in SMR02
Previous_Caesarean_section	SMR02	**1:** More than zero<br>**0:** Zero	http://www.ndc.scot.nhs.uk/Dictionary-A-Z/Definitions/index.asp?Search=P&ID=406&Title=Previous Caesarean Sections

For peer review only

STROBE (Strengthening The Reporting of OBservational Studies in Epidemiology) Checklist

A checklist of items that should be included in reports of observational studies. You must report the page number in your manuscript where you consider each of the items listed in this checklist. If you have not included this information, either revise your manuscript accordingly before submitting or note N/A.

Note: An Explanation and Elaboration article discusses each checklist item and gives methodological background and published examples of transparent reporting. The STROBE checklist is best used in conjunction with this article (freely available on the Web sites of PLoS Medicine at http://www.plosmedicine.org/, Annals of Internal Medicine at http://www.annals.org/, and Epidemiology at http://www.epidem.com/). Information on the STROBE Initiative is available at www.strobe-statement.org.

Section and Item	Item No.	Recommendation	Reported on Page No.
**Title and Abstract**	1	(*a*) Indicate the study's design with a commonly used term in the title or the abstract	1
		(*b*) Provide in the abstract an informative and balanced summary of what was done and what was found	2
**Introduction**			
Background/Rationale	2	Explain the scientific background and rationale for the investigation being reported	4,5
Objectives	3	State specific objectives, including any prespecified hypotheses	5
**Methods**			
Study Design	4	Present key elements of study design early in the paper	5
Setting	5	Describe the setting, locations, and relevant dates, including periods of recruitment, exposure, follow-up, and data collection	5,6,7
Participants	6	(*a*) *Cohort study*—Give the eligibility criteria, and the sources and methods of selection of participants. Describe methods of follow-up	5,6,7
		Case-control study—Give the eligibility criteria, and the sources and methods of case ascertainment and control selection. Give the rationale for the choice of cases and controls	N/A
		Cross-sectional study—Give the eligibility criteria, and the sources and methods of selection of participants	N/A
		(*b*) *Cohort study*—For matched studies, give matching criteria and number of exposed and unexposed	N/A
		Case-control study—For matched studies, give matching criteria and the number of controls per case	N/A
Variables	7	Clearly define all outcomes, exposures, predictors, potential confounders, and effect modifiers. Give diagnostic criteria, if applicable	6,7

For peer review only

Section and Item	Item No.	Recommendation	Reported on Page No.
Data Sources/ Measurement	8*	For each variable of interest, give sources of data and details of methods of assessment (measurement). Describe comparability of assessment methods if there is more than one group	5,6,7
Bias	9	Describe any efforts to address potential sources of bias	8,9
Study Size	10	Explain how the study size was arrived at	5
Quantitative Variables	11	Explain how quantitative variables were handled in the analyses. If applicable, describe which groupings were chosen and why	8,9
Statistical Methods	12	(*a*) Describe all statistical methods, including those used to control for confounding	8,9
		(*b*) Describe any methods used to examine subgroups and interactions	N/A
		(*c*) Explain how missing data were addressed	7,16
		(*d*) *Cohort study*—If applicable, explain how loss to follow-up was addressed	N/A
		Case-control study—If applicable, explain how matching of cases and controls was addressed	N/A
		Cross-sectional study—If applicable, describe analytical methods taking account of sampling strategy	N/A
		(*e*) Describe any sensitivity analyses	N/A
**Results**			
Participants	13*	(a) Report numbers of individuals at each stage of study—eg numbers potentially eligible, examined for eligibility, confirmed eligible, included in the study, completing follow-up, and analysed	11
		(b) Give reasons for non-participation at each stage	N/A
		(c) Consider use of a flow diagram	Figure 1
Descriptive Data	14*	(a) Give characteristics of study participants (eg demographic, clinical, social) and information on exposures and potential confounders	5,6,7,8
		(b) Indicate number of participants with missing data for each variable of interest	11
		(c) *Cohort study*—Summarise follow-up time (eg, average and total amount)	5,6
Outcome Data	15*	*Cohort study*—Report numbers of outcome events or summary measures over time	6,7
		Case-control study—Report numbers in each exposure category, or summary measures of exposure	N/A
		Cross-sectional study—Report numbers of outcome events or summary measures	N/A

For peer review only

Section and Item	Item No.	Recommendation	Reported on Page No.
Main Results	16	(*a*) Give unadjusted estimates and, if applicable, confounder-adjusted estimates and their precision (eg, 95% confidence interval). Make clear which confounders were adjusted for and why they were included	9,10,11,12,13
		(*b*) Report category boundaries when continuous variables were categorized	8
		(*c*) If relevant, consider translating estimates of relative risk into absolute risk for a meaningful time period	N/A
Other Analyses	17	Report other analyses done—eg analyses of subgroups and interactions, and sensitivity analyses	N/A
**Discussion**			
Key Results	18	Summarise key results with reference to study objectives	13
Limitations	19	Discuss limitations of the study, taking into account sources of potential bias or imprecision. Discuss both direction and magnitude of any potential bias	16
Interpretation	20	Give a cautious overall interpretation of results considering objectives, limitations, multiplicity of analyses, results from similar studies, and other relevant evidence	13,14,15
Generalisability	21	Discuss the generalisability (external validity) of the study results	13
**Other Information**			
Funding	22	Give the source of funding and the role of the funders for the present study and, if applicable, for the original study on which the present article is based	20

*Give information separately for cases and controls in case-control studies and, if applicable, for exposed and unexposed groups in cohort and cross-sectional studies.

Once you have completed this checklist, please save a copy and upload it as part of your submission. DO NOT include this checklist as part of the main manuscript document. It must be uploaded as a separate file.

For peer review only

BMJ Open

A cohort study of high maternal Body Mass Index and the risk of adverse pregnancy and delivery outcomes in Scotland

Journal:	*BMJ Open*
Manuscript ID	bmjopen-2018-026168.R2
Article Type:	Original research
Date Submitted by the Author:	26-Aug-2019
Complete List of Authors:	Doi, Lawrence; University of Edinburgh, Scottish Collaboration for Public Health Research and Policy, School of Health in Social Science<br>Williams, Andrew James; University of Exeter<br>Marryat, Louise; University of Edinburgh, SCPHRP<br>Frank, John; University of Edinburgh, Scottish Collaboration for Public Health Research and Policy
**Primary Subject Heading**:	Obstetrics and gynaecology
Secondary Subject Heading:	Epidemiology, Paediatrics, Public health, Reproductive medicine, Health policy
Keywords:	OBSTETRICS, EPIDEMIOLOGY, PUBLIC HEALTH

SCHOLARONE™
Manuscripts

I, the Submitting Author has the right to grant and does grant on behalf of all authors of the Work (as defined in the below author licence), an exclusive licence and/or a non-exclusive licence for contributions from authors who are: i) UK Crown employees; ii) where BMJ has agreed a CC-BY licence shall apply, and/or iii) in accordance with the terms applicable for US Federal Government officers or employees acting as part of their official duties; on a worldwide, perpetual, irrevocable, royalty-free basis to BMJ Publishing Group Ltd ("BMJ") its licensees and where the relevant Journal is co-owned by BMJ to the co-owners of the Journal, to publish the Work in this journal and any other BMJ products and to exploit all rights, as set out in our licence.

The Submitting Author accepts and understands that any supply made under these terms is made by BMJ to the Submitting Author unless you are acting as an employee on behalf of your employer or a postgraduate student of an affiliated institution which is paying any applicable article publishing charge ("APC") for Open Access articles. Where the Submitting Author wishes to make the Work available on an Open Access basis (and intends to pay the relevant APC), the terms of reuse of such Open Access shall be governed by a Creative Commons licence – details of these licences and which Creative Commons licence will apply to this Work are set out in our licence referred to above.

Other than as permitted in any relevant BMJ Author's Self Archiving Policies, I confirm this Work has not been accepted for publication elsewhere, is not being considered for publication elsewhere and does not duplicate material already published. I confirm all authors consent to publication of this Work and authorise the granting of this licence.

For peer review only

A cohort study of high maternal Body Mass Index and the risk of adverse pregnancy and delivery outcomes in Scotland

Lawrence Doi[1]*, Andrew James Williams[2], Louise Marryat[3,4], John Frank[3,5]

*Correspondence: larry.doi@ed.ac.uk

[1]Scottish Collaboration for Public Health Research and Policy, School of Health in Social Science, University of Edinburgh, Doorway 6, Old Medical School, Teviot Place, EH8 9AG, UK.

[2]European Centre for Environment and Human Health, College of Medicine and Health, University of Exeter, Knowledge Spa, Royal Cornwall Hospital, Truro, Cornwall TR1 3HD, UK.

[3]Farr Institute at Scotland, University of Edinburgh, Nine, Edinburgh BioQuarter, 9 Little France Road, Edinburgh EH16 4UX, UK.

[4]Centre for Clinical Brain Sciences, University of Edinburgh, Royal Edinburgh Hospital, Kennedy Tower, Morningside Park, Edinburgh EH10 5HF, UK.

[5]Usher Institute of Population Health Sciences and Informatics, University of Edinburgh, Doorway 1, Old Medical School, Teviot Place, EH8 9AG, UK.

For peer review only

Abstract

Objective: To examine the association between high maternal weight status and complications during pregnancy and delivery.

Setting: Scotland

Participants: Data from 135,860 first time singleton deliveries in Scotland between 2008 and 2015 were used. Women with overweight and obesity were compared with women with normal weight. Associations between maternal body mass index and complications during pregnancy and delivery were evaluated.

Outcome measures: Gestational diabetes, gestational hypertension, pre-eclampsia, placenta praevia, placental abruption, induction of labour, elective and emergency caesarean sections, pre-term delivery, post-term delivery, low Apgar score, small for gestational age and large for gestational age.

Results: In the multivariable models controlling for potential covariates, we found that compared with women with normal weight, the odds of the following outcomes were significantly increased for women with overweight and obesity [overweight adjusted odds ratio; 95% confidence interval, followed by the same for women with obesity]: gestational hypertension [1.61; 1.49-1.74], [2.48; 2.30-2.68]; gestational diabetes [2.14; 1.86-2.46], [8.25; 7.33-9.30]; pre-eclampsia [1.46; 1.32-1.63] [2.07; 1.87-2.29]]; labour induction [1.28; 1.23-1.33], [1.69; 1.62-1.76] and emergency caesarean section [1.82; 1.74-1.91], [3.14; 3.00-3.29].

Conclusions: Women with overweight and obesity in Scotland are at greater risk of adverse pregnancy and delivery outcomes. The risk of these conditions increases steadily with increasing body mass index. Health professionals should be

For peer review only - http://bmjopen.bmj.com/site/about/guidelines.xhtml

empowered and trained to deliver promising dietary and lifestyle interventions to women at risk of overweight and obesity prior to conception, and control excessive weight gain in pregnancy.

Strengths and limitations of this study

- This study used a large, retrospectively accessed but cohort-structured, national database covering all major maternal and neonatal outcomes in Scotland over eight recent years.
- Analysis used whole study population with adequate adjustment for confounders to estimate impact of high maternal-weight status on each outcome.
- All women with BMI of 30 kg/m^2 or more were considered as having obesity; it is likely that differentiating morbid obesity or obesity class II and III from obesity would have generated more precise risk estimates.
- The completeness of the recording of BMI increased during the study period (2008 to 2015) from 69% to 98%. Using data from the earlier years when the BMI was missing more often might have biased the study sample if it was the case that BMI was not missing at random.

For peer review only - http://bmjopen.bmj.com/site/about/guidelines.xhtml

Introduction

The increasing global prevalence of overweight and obesity makes it more likely that a growing number of women with high body mass index (BMI) are becoming pregnant. High maternal BMI during pregnancy has adverse health implications for women (1,2) and predisposes the unborn child to adverse outcomes, including neonatal deaths, stillbirth, and admission to a neonatal unit (3,4,5). A recent international systematic review involving 38 studies found that even modest increases in maternal BMI were associated with increased risk of fetal death, stillbirth, and neonatal, perinatal, and infant death (6). Obesity in pregnancy can have health implications later in life for both mother and child. Among women this could lead to diabetes, heart disease and hypertension, whilst children are more prone to future obesity and hypertension (7). Wider service and economic consequences can also be seen including indirect burdens of illness and associated costs, in that women with high BMI also experience adverse birth outcomes such as neonatal death or stillbirth (8,9,10). Such individuals may require support from family and friends as well as bereavement support services within the community. There are also substantial healthcare cost implications for infants who may require medical attention. For example, a recent study examining infant health utilisation and costs on the NHS in the UK of infants born to women with overweight or obesity found that total mean additional resource cost for infants born to women who are overweight was £65.13, and £1138.11 for infants born to women who are obese (11).

For peer review only - http://bmjopen.bmj.com/site/about/guidelines.xhtml

Maternal weights are currently high in Scotland: a recent study reported that 31.5% of mothers were overweight and a further 23.6% were affected by obesity (12). Another study examining the impact of maternal BMI on clinical complications, inpatient admissions, and additional short-term costs to the NHS in Scotland revealed that maternal BMI influences maternal and neonatal morbidity, the number and duration of maternal and neonatal admissions, and health service costs (13). However, no recent study in Scotland has investigated the effect of high maternal BMI on the risk of pregnancy and delivery outcomes. The aim of this study was to examine the associations between high maternal BMI and complications during pregnancy and delivery in Scotland. Understanding of these associations can highlight areas where prevention strategies could be targeted.

Methods

Study population and data sources

This retrospective cohort study used data from 135,860 first time mothers who gave birth to only one child in Scotland between January 2008 and December 2015. The women and infants were identified within three electronic medical record databases: the Scottish Morbidity Record (SMR) 01 and 02 and Scottish Birth Record (SBR). SMR01 is generated for patients receiving inpatient or day care in the General or Acute specialties, whilst SMR02 is generated for patients receiving inpatient or day care in the Obstetric Specialties. The SBR records all of a baby's neonatal care in Scotland. The outcome variables are recorded in these databases according the World Health Organization's International Classification of Diseases, Tenth Revision (ICD-10). Further description of the content of these databases is available (14) and

For peer review only - http://bmjopen.bmj.com/site/about/guidelines.xhtml

in the web-appendix. The study was designed as a clinical audit so did not require approval from a Research Ethics Committee. However, approval was obtained from the Public Benefit and Privacy Panel via the national Electronic Data Research and Innovation Service to use the anonymised data collected by these registries.

Patient and public involvement

Patients were not involved in the design, analysis and interpretation of this study.

Exposure variable

More than 80% of pregnant women in Scotland present themselves for antenatal care during the first trimester of their pregnancy. Height and weight are measured by the midwife at the first antenatal visit, typically before 12 weeks of pregnancy. Body mass index was calculated using the formula weight (kg)/height (m^2). BMI completeness was 69% in 2008 but this increased gradually to 87% in 2011 when recording of weight and height became mandatory. By 2015 BMI completeness was 98%. Women were categorised into three BMI groups as described in Table 1.

Table 1. Adult BMI definitions used

**Variable**	**Category**	**BMI Definition**
Maternal weight status	Normal	<25 kg/m^2
	Overweight	≥25 kg/m^2 to <30 kg/m^2
	Obesity	≥30 kg/m^2

Outcomes

Outcome measures included were maternal or pregnancy complications such as gestational diabetes, gestational hypertension and pre-eclampsia (high blood pressure and protein in urine). Conditions affecting delivery or delivery complications

For peer review only - http://bmjopen.bmj.com/site/about/guidelines.xhtml

For peer review only

studied were placenta praevia (when a baby's placenta partially or totally covers the mother's cervix), placental abruption (when the placenta separates early from the uterus before childbirth), induction of labour, caesarean delivery (includes elective and emergency caesarean sections), pre-term delivery (defined as less than 37 weeks of gestation), post-term delivery (more than 42 weeks of gestation), low Apgar score (less than “7” at 5 minutes), small for gestational age (SGA), and large for gestational age (LGA). SGA were infants with birthweight of ≤10th percentile for gestational age according to UK1990 growth reference curve (15,16), and those with LGA were infants with birth weight ≥90th percentile.

Covariates

Maternal age at delivery, smoking during pregnancy and Carstairs 2001 quintiles for Scotland for the postcode sector of the mother’s residence at birth, were considered as potentially confounding variables and were included as covariates in the adjusted analyses. Table 2 describes the covariates used in this study by maternal weight status among singleton (a pregnancy with one fetus as opposed to twins or multiples) first-time pregnancies. The data in Table 2 show the numbers of women who had data on age, deprivation and maternal smoking. This means that the total number of women shown is slightly larger than the number of women ananlysed as there were women missing some outcome data.

Table 2. Maternal characteristics among normal weight, overweight and obese women[+] (singleton, first pregnancies)

For peer review only - http://bmjopen.bmj.com/site/about/guidelines.xhtml

	Normal* N = 73,130		Overweight* N = 36,992		Obese* N = 25,738	
	N	%	N	%	N	%
**Maternal age (y)**						
20-24	19,874	27.2	9,368	25.3	7,016	27.3
25-29	24,443	33.4	12,174	32.9	8,475	32.9
30-34	20,880	28.6	10,522	28.4	6,922	26.9
35-39	7,933	10.9	4,928	13.3	3,325	12.9
**Carstairs 2001 quintiles for Scotland**						
Q1 (Least deprived)	14,695	20.1	6,785	18.3	3,870	15.0
Q2	13,820	18.9	6,851	18.5	4,639	18.0
Q3	14,581	19.9	7,549	20.4	5,117	19.9
Q4	15,338	21.0	7,994	21.6	6,079	23.6
Q5 (Most deprived)	14,696	20.1	7,813	21.1	6,033	23.4
**Maternal smoking in pregnancy**						
No	62,439	85.4	31,806	86.0	21,604	83.9
Yes	10,691	14.6	5,186	14.0	4,134	16.1

*Maternal weight status at first antenatal visit
+Figures show women who had complete data on age, deprivation and maternal smoking

Data analysis

Using Stata 14 (17), logistic regression was used to calculate odds ratios (ORs). BMI groups with overweight and obesity were compared with the normal BMI group (the reference population). A confidence interval (CI) of 95% was produced for all ORs. The analysis of the outcomes proceeded in a systematic approach.

For the first group of outcomes (gestational diabetes, hypertension and pre-eclampsia) the models were adjusted for confounders, and the two other conditions that were not the dependent variable as these conditions can co-occur. This approach was taken throughout the analysis, adjusting for conditions, which precede or occur contemporarily with the outcome being examined as the dependent variable. Unless, the outcomes could not co-occur such as pre-term and post-term, SGA and LGA, or method of delivery (induction, elective or emergency c-section).

For peer review only - http://bmjopen.bmj.com/site/about/guidelines.xhtml

Results

Within our study population 53.8 % of pregnant women were categorised as normal weight, 27.3% overweight and 18.9% were affected by obesity. The socio-demographic characteristics of the women in the three BMI categories are presented in Table 2. Maternal smoking prevalence was slightly higher among women with obesity than in women with normal weight or overweight. Among the women who were overweight, 21.1% were from the most deprived and 18.3% were from the least deprived group. However, the difference in social deprivation was more marked within women with obesity. Among this group, 23.4% were from the most deprived group whilst 15.0% were from the least deprived group.

Table 3 shows odd ratios for pregnancy and delivery complications, among women who were overweight or obese, and with singleton first-time births. The risk of the three conditions occurring during pregnancy increased steadily with increasing BMI. For example, compared to the normal BMI group, the risk of gestational diabetes was 2.14 (95% CI: 1.86-2.46) but among women who were obese the risk increased to 8.25 (95% CI: 7.33 – 9.30). Relative to women who were of normal weight, the adjusted OR of pre-eclampsia for women who were overweight was 1.46 (95% CI: 1.32-1.62) and 2.07 (95% CI: 1.87-2.29) for women who were obese.

For peer review only - http://bmjopen.bmj.com/site/about/guidelines.xhtml

Table 3. Pregnancy and delivery complications among normal, overweight and obese singleton women

	Total	Normal		Overweight			Obese		
	**sample**	**N (%)**	**Cases (%)**	**N (%)**	**Cases (%)**	**Adjusted OR* (95% CI)**	**N (%)**	**Cases (%)**	**Adjusted OR* (95% CI)**
**Conditions occurring during pregnancy**									
Gestational hypertension	132,899	71,538 (53.8)	1,550 (2.2)	36,188 (27.2)	1,239 (3.4)	1.61 (1.49, 1.74)	25,173 (18.9)	1,275 (5.1)	2.48 (2.30, 2.68)
Gestational diabetes	132,899	71,538 (53.8)	377 (0.5)	36,188 (27.2)	418 (1.2)	2.14 (1.86, 2.46)	25,173 (18.9)	1,082 (4.3)	8.25 (7.33, 9.30)
Pre-eclampsia	132,899	71,538 (53.8)	906 (1.3)	36,188 (27.2)	664 (1.8)	1.46 (1.32, 1.62)	25,173 (18.9)	640 (2.5)	2.07 (1.87, 2.29)
**Conditions affecting delivery**									
Placenta praevia[1]	132,212	71,172 (53.8)	102 (0.1)	36,001 (27.2)	66 (0.2)	1.23 (0.90, 1.68)	25,039 (18.9)	30 (0.1)	0.81 (0.54, 1.22)
Placental abruption[1]	132,212	71,172 (53.8)	133 (0.2)	36,001 (27.2)	55 (0.2)	0.81 (0.59, 1.11)	25,039 (18.9)	38 (0.2)	0.76 (0.53, 1.10)
Small for gestational age[1]	132,212	71,172 (53.8)	6,664 (9.4)	36,001 (27.2)	2,645 (7.4)	0.76 (0.72, 0.80)	25,039 (18.9)	1,726 (6.9)	0.69 (0.65, 0.73)
Large for gestational age[1]	132,212	71,172 (53.8)	17,852 (25.1)	36,001 (27.2)	10,879 (30.2)	1.30 (1.26, 1.33)	25,039 (18.9)	8,575 (34.3)	1.57 (1.52, 1.62)
Pre-term	132,212	71,172 (53.8)	4,295 (6.0)	36,001 (27.2)	2,231 (6.2)	1.02 (0.96, 1.07)	25,039 (18.9)	1,725 (6.9)	1.11 (1.05, 1.18)
Post-term[2]	78,074	43,486 (55.7)	31 (0.1)	20,973 (26.9)	24 (0.1)	1.57 (0.93, 2.68)	13,615(17.4)	14 (0.1)	1.47 (0.78, 2.77)
**Delivery**									
Induction of labour	92,967	53,617 (57.7)	13,417 (25.0)	24,342 (26.2)	7,420 (30.5)	1.28 (1.23, 1.33)	15,008 (16.1)	5,712 (38.1)	1.69 (1.62, 1.76)
Caesarean section	90,183	51,798 (57.4)	11,598 (22.4)	23,827 (26.4)	6,905 (29.0)	1.34 (1.29, 1.39)	14,558 (16.1)	5.262 (36.2)	1.80 (1.73, 1.88)
Emergency caesarean section	80,938	45,715 (56.5)	5,515 (12.1)	21,397 (26.4)	4,475 (20.9)	1.82 (1.74, 1.91)	13,826 (17.1)	4,530 (32.8)	3.14 (3.00, 3.29)
Apgar score	129,773	70,012 (54.0)	12,583 (18.0)	35,307 (27.2)	6,125 (17.4)	0.95 (0.92, 0.99)	24,454 (18.8)	4,342 (17.8)	0.96 (0.93, 1.00)

OR = Odd Ratio; CI = Confidence Interval; NICU = Neonatal Intensive Care Unit

*Adjusted for maternal age, deprivation and smoking in pregnancy

[1]80 post-term births were excluded from these models as none of them experienced the outcome being estimated

[2]No participants in the study had placenta praevia, placental abruption or had small or large for gestational age babies and were post-term and therefore 45,957 participants were dropped from this analysis

For peer review only

Regarding conditions affecting delivery, the odds ratio of placenta praevia were not statistically significant different for both women who were overweight (OR 1.23, 95%CI: 0.90-1.68) and obese (OR 0.81, 95%CI: 0.54-1.22) compared with women with normal weight. The odds of experiencing placental abruption were also not significantly different among the different BMI categories.

In contrast with the normal BMI group, births to women who were overweight and obese were associated with decreased risk of small-for-gestational age, ORs 0.76 (95% CI: 0.72-0.80) and 0.69 (95% CI: 0.65-0.73) respectively. However, the prevalence of large-for-gestational age increased among women with overweight, OR of 1.30 (95%CI: 1.26-1.33) and women with obesity, OR 1.57 (95% CI: 1.52-1.62) compared with women of normal weight.

The chance of induction of labour and caesarean section, either elective or emergency increased with increasing BMI. Women who were overweight had odds of 1.34 (95% CI: 1.29-1.39) higher for having an elective Caesarean section and higher odds (OR 1.82, 95% CI: 1.74-1.91) for undergoing emergency Caesarean section, compared with women of normal weight. The corresponding ORs for women with obesity were 1.80 (95% CI: 1.73-1.88) and 3.14 (95% CI: 3.00-3.29). Being overweight or obese was associated with reduced risk of low Apgar score. This was barely significant for women who were overweight (OR 0.95 , 95% CI: 0.92-0.99) and not obese (OR 0.96, 95% CI: 0.93-1.00).

For peer review only - http://bmjopen.bmj.com/site/about/guidelines.xhtml

For peer review only

Discussion

In this large, retrospective cohort study, we found that overweight or obesity during pregnancy increased the risk of several adverse pregnancy and delivery complications. Some earlier studies have also found similar findings. Aside from obesity, we also examined overweight because in most populations, a greater number of women are overweight rather than obese, so it is important to also understand the impact of overweight on pregnancy and neonatal outcomes.

In terms of the association between high maternal BMI and conditions that occur during pregnancy, we found that the risk of all the conditions considered (gestational hypertension, gestational diabetes and pre-eclampsia) increased steadily with increasing BMI, which is in line with similar studies (2,3,4). A study compared women of normal weight to women who were morbidly obese (BMI greater than 40), and also found that there was an increased risk of pre-eclampsia (OR 4.82; 95% CI: 4.04-5.74) (3). Our study also found that, aside from heightened pre-eclampsia risk for women with obesity, being overweight was also significantly associated with this outcome, albeit to a lesser degree. A meta-analysis of the association between maternal BMI and the risk of pre-eclampsia showed that the risk doubled with each 5-7 kg/m^2 increase in pre-pregnancy BMI (18). It is evident that the risk of pre-eclampsia increases with the degree of weight gain; therefore preventative strategies should be focussed on getting women, especially those already overweight, to reduce weight prior to conception. Weight loss in pregnancy requires careful management in order to avoid unintended consequences (19). Nevertheless, women often engage with health professionals during pregnancy; therefore dietary and lifestyle interventions such as physical activity, which have been shown by reviews

For peer review only - http://bmjopen.bmj.com/site/about/guidelines.xhtml

and meta-analyses (19,20) to reduce gestational weight gain and improve outcomes for both mother and baby, could be provided to them.

Generally, rates of Caesarean delivery have increased significantly across many developed countries in recent years (21). Our study found that women with overweight and obesity showed an increased risk of Caesarean delivery (both elective and emergency) compared to normal-weight women, but we note that the overall frequency of Caesarean delivery across our obstetric population seems quite high, compared with a previous Swedish study (3). Possible reasons could be that Caesarean delivery is probably less risky now, due to advances in medical science, which facilitate accurate monitoring of the progress of labour and fetal intra-partum condition (22). It is also possible that health professionals in Scotland are probably intervening early with regards to problems in labour among women with overweight or obesity, in order to reduce fetal distress, and its worst outcomes. Nevertheless, this is quite concerning for Scotland, which has invested in programmes aimed at promoting natural birth, such as Keeping Childbirth Natural and Dynamic (KCND). The KCND is a maternity care programme introduced by the Scottish Government with the aim of maximising opportunities for women to have as natural a birth experience as possible, reduce unnecessary interventions in low-risk pregnancy and childbirth, and to provide women-centred care (23,24,25). The early intervention in pregnancy may also explain the reduced risk of low Apgar score for infants born to women with overweight and obesity. It is likely that these women are may receive increased monitoring, which means issues can be identified and managed earlier, to reduce any fetal distress in labour.

For peer review only - http://bmjopen.bmj.com/site/about/guidelines.xhtml

Adiposity increases risk of large-for-gestational age and macrosomia; in this study, we found that births to women with overweight and obesity were associated with an increased risk of large-for-gestational age infants compared with women of normal weight. Excess weight in pregnancy may shift the entire birth weight distribution upwards, perhaps through hormonal mechanisms that operate at lower levels than in full-blown cases of macrosomia in infants of diabetic mothers.

Strengths and limitations

This study used a large, retrospectively accessed but cohort-structured, national database covering several maternal and neonatal outcomes. The analysis used whole study population with adequate adjustment for confounders to estimate impact of high maternal-weight status on each outcome. We restricted the analysis to only single births and first pregnancies to ensure that the births in the sample are relatively independent. The dataset we used combined underweight and normal weight women as normal BMI group. Using this as reference group might have strengthened the association between high maternal BMI and the pregnancy and neonatal outcomes considered. However, only a very small number of women are underweight during pregnancy in Scotland in recent years. Also, some studies differentiate between different obesity categories, but in this study all women with BMI of 30 or more were considered as having obesity. It is likely that differentiating morbid obesity, or obesity class II and III from women with obesity, would have generated additional insight. The completeness of the recording of BMI increased during the study period (2008 to 2015) from 69% to 98%. Using data from the earlier years, when the BMI was missing more often, might have biased the study sample if BMI was not missing at random. The study included control for a limited set of

For peer review only - http://bmjopen.bmj.com/site/about/guidelines.xhtml
For peer review only

confounders and inclusion of other relevant confounders could have strengthened the analysis. For example, variables such as ethnicity, previous caesarean sections, time of birth were not available in dataset, which we accessed. Also, we could not use neonatal outcomes such as stillbirth, neonatal death and congenital anomaly because these outcomes are not completely ascertained in the dataset we used.

Conclusion

This study has shown that women who were overweight and obese in Scotland are at greater risk of several pregnancy and delivery complications including gestational hypertension, gestational diabetes, pre-eclampsia, labour induction and Caesarean delivery. The risk of these conditions increases steadily with increasing BMI. Health professionals should be empowered and trained to deliver promising dietary and lifestyle interventions to women at risk of overweight and obesity prior to conception, and control excessive weight gain in pregnancy.

References

1. Rooney BL, Schauberger CW. Excess pregnancy weight gain and long-term obesity: one decade later. *Obstet Gynecol* 2002; **100(2):** 245-252.
2. Kumari P, Gupta M, Kahlon P, Malviya S. Association between high maternal body mass index and fetomaternal Outcome. *J Obes Metab Res* 2014; **1:** 143-148.
3. Cedergren MI. Maternal morbid obesity and the risk of adverse pregnancy outcome. *Obstetricians and Gynecologists* 2004; **103:** 219-224.
4. Scott-Pillai R, Spence D, Cardwell C, Hunter A, Holmes V. The impact of body mass index on maternal and neonatal outcomes: a retrospective study in a UK obstetric population, 2004–2011. *BJOG* 2013; **120:** 932–939.

For peer review only

5. Rahman MM, Abe SK, Kanda M, Narita S, Rahman MS, Bilano V, Ota E, Gilmour S, Shibuya K. Maternal body mass index and the risk of birth and maternal health outcomes in low- and middle-income countries: a systematic review and meta-analysis. *Obesity Reviews* 2015; **16:** 758–770.
6. Aune D, Saugstad OD, Henriksen T, Tonstad S. Maternal body mass index and the risk of fetal death, stillbirth, and infant death: a systematic review and meta-analysis. *JAMA* 2014; **311**(15):1536-46.
7. Leddy MA, Power ML, Schulkin J. The impact of maternal obesity on maternal and fetal health. *Rev Obstet Gynecol*. 2008;**1**(4):170–178.
8. Heslehurt N, Lang R, Rankin J, Wilkinson J, Summerbell CD. Obesity in pregnancy: a study of the impact of maternal obesity on NHS maternity services. *BJOG* 2007; **114(3):** 334-342.
9. Furness PJ, McSeveny K, Arden MA, Garland C, Dearden AM, Soltani H. Maternal obesity support services: a qualitative study of the perspectives of women and midwives. *BMC Pregnancy and Childbirth* 2011; **11:** 69.
10. Lenoir-Wijnkoop I, van der Beek EM, J Garssen, Nuijten MJC, Uauy RD. Health economic modeling to assess short-term costs of maternal overweight, gestational diabetes, and related macrosomia – a pilot evaluation. *Front Pharmacol* 2015; **6:**103. doi: 10.3389/fphar.2015.00103.
11. Morgan KL, Rahman MA, Hill RA, et al. Obesity in pregnancy: infant health service utilisation and costs on the NHS. *BMJ Open* 2015;5:e008357. doi:10.1136/bmjopen-2015- 008357.
12. Doi L, Williams AJ, Frank J. How has child growth around adiposity rebound altered in Scotland since 1990 and what are the risk factors for weight gain

For peer review only - http://bmjopen.bmj.com/site/about/guidelines.xhtml

using the Growing Up in Scotland birth cohort 1? *BMC Public Health* 2016; **16:** 1081.

13. Denison FC, Norwood P, Bhattacharya S, Duffy A, Mahmood T, Morris C, Raja EA, Norman JE, Lee AJ, Scotland G. Association between maternal body mass index during pregnancy, short-term morbidity, and increased health service costs: a population-based study. *BJOG* 2014; **121**:72–82.
14. Information Service Division (2018). Scottish birth record. http://www.ndc.scot.nhs.uk/Data-Dictionary/SMR-Datasets/Scottish-Birth-Record/
15. Cole TJ, Freeman JV, Preece MA. Body mass index reference curves for the UK, 1990. *Arch Dis Child* 1995; **73(1):** 25–29.
16. Freeman JV, Cole TJ, Chinn S, Jones PRM, White EM, Preece MA. Cross-sectional stature and weight reference curves for the UK 1990. *Arch Dis Child* 1995; **73(1):**17–24.
17. StataCorp. Stata statistical software: release 14. College Station: StataCorp LP; 2015.
18. O'Brien TE, Ray JG, Chan WS. Maternal body mass index and the risk of preeclampsia: A systematic overview. *Epidemiology* 2003; **14:** 368-374.
19. Shieh C, Cullen DL, Pike C, Pressler SJ. Intervention strategies for preventing excessive gestational weight gain: systematic review and meta-analysis. *Obesity Reviews* 2018; doi: 10.1111/obr.12691.
20. Thangaratinam S, Rogozinska E, Jolly K, Glinkowski S, Roseboom T, Tomlinson JW, Kunz R, Mol BW, Coomarasamy A, Khan KS. Effects of interventions in pregnancy on maternal weight and obstetric outcomes: Meta-analysis of randomised evidence. *BMJ.* 2012; **344:** e2088.

For peer review only - http://bmjopen.bmj.com/site/about/guidelines.xhtml

21. Althabe F, Sosa C, Belizán JM, Gibbons L, Jacquerioz F, Bergel E. Cesarean section rates and maternal and neonatal mortality in low-, medium-, and high-income countries: an ecological study. *Birth* 2006; **33(4):** 270-277.
22. Rezaie M, Shahoe R, Shahghebi S. The effect of maternal body mass index on the delivery route in nulliparous women. *Journal of Public Health and Epidemiology* 2013; **5(8):** 346-350.
23. Health Improvement Scotland (2009). Pathways for maternity care. http://www.healthcareimprovementscotland.org/default.aspx?page=12536
24. Abhyankar P, Cheyne H, Maxwell M, McCourt C. A realist evaluation of a normal birth programme. *Evidence Based Midwifery* 2013; https://www.rcm.org.uk/learning-and-career/learning-and-research/ebm-articles/a-realist-evaluation-of-a-normal-birth.
25. Cheyne H, Abhyankar P, McCourt C. Empowering change: realist evaluation of a Scottish Government programme to support normal birth. *Midwifery* 2013; **29:** 1110–1121.

Funding

This work was funded by the SCPHRP core grant from the Medical Research Council (Grant Number MR/K023209/1) and the Chief Scientist Office of Scotland. AJW is supported by the European Centre for Environment and Human Health, University of Exeter. LM is supported by the Farr Institute @ Scotland, which is supported by a 10-funder consortium: Arthritis Research UK, the British Heart

For peer review only - http://bmjopen.bmj.com/site/about/guidelines.xhtml

Foundation, Cancer Research UK, the Economic and Social Research Council, the Engineering and Physical Sciences Research Council, the Medical Research Council, the National Institute of Health Research, the National Institute for Social Care and Health Research (Welsh Assembly Government), the Chief Scientist Office (Scottish Government Health Directorates), (MRC Grant No: MR/K007017/1). The funders played no role in the conceptualisation or realisation of the research and no role in the decision to submit it for publication.

For peer review only

Competing interest

The authors declare that they have no competing interests.

Data sharing

Data used was categorised as confidential data release.

Acknowledgements

The authors are grateful to the Electronic Data Research and Innovation Service of the Information Services Division, NHS National Services Scotland for providing the data used in this paper.

Authors' contributions

LD, AJW and JF conceived the original idea for the study and obtained the data. AJW led the statistical analysis with support from LD, LM and JF. LD wrote the first draft of the paper and all authors revised successive drafts and approved the final manuscript.

For peer review only - http://bmjopen.bmj.com/site/about/guidelines.xhtml

Web-appendix: Description of data fields used in the study

**Variable Name**	**Database Source**	**Variable Description/Values**	**Notes**
Mother_ID	N/A - study specific	Unique mother identifier	1516-0613/' followed by an anonymous identifier
Baby_ID	N/A - study specific	Unique baby identifier	Mother_ID followed by delivery sequence number followed by a baby sequence number. The baby sequence number for multiple babies from same delivery not necessarily in correct order due to missing CHI numbers.
Delivery_Seq_No	N/A - study specific	Delivery sequence number of mother	
Gest_Diabetes	SMR02	**1:** Yes, Gestational diabetes (diagnosed during this pregnancy)<br>**0:** Yes, Pre-existing diabetes (diagnosed before pregnancy) & No (no diabetes during this pregnancy)<br>**Missing:** Yes, Time of diagnosis unknown & Not Known	http://www.ndc.scot.nhs.uk/Dictionary-A-Z/Definitions/index.asp?Search=D&ID=214&Title=Diabetes
Gest_Hypertension	SMR02/SMR01	**1:** ICD 10 code O13.X<br>**0:** All other codes	Flagged codes cover gestational hypertension
Pre_Eclampsia	SMR02/SMR01	**1:** ICD 10 code O14._<br>**0:** All other codes	Flagged codes cover pre-eclampsia
Placental_Abruption	SMR02/SMR01	**1:** ICD 10 code O45._<br>**0:** All other codes	Flagged codes cover placental abruption
Placental_Praevia	SMR02/SMR01	**1:** ICD 10 code O44._<br>**0:** All other codes	Flagged codes cover placenta praevia

For peer review only

Caesarean_Delivery	SMR02	**1:** Elective (planned) caesarean section & Emergency and unspecified caesarean section<br>**0:** All other codes	http://www.ndc.scot.nhs.uk/Dictionary-A-Z/Definitions/index.asp?Search=M&ID=322&Title=Mode of Delivery - Babies 1 to 3
Labour_Induction	SMR02	**1:** 1-8 - Induction of labour codes<br>**0:** 0, None<br>**Missing:** 9, Not known	http://www.ndc.scot.nhs.uk/Dictionary-A-Z/Definitions/index.asp?Search=I&ID=295&Title=Induction of Labour
SGA	SMR02	**1:** Birthweight ≤10th percentile<br>**0:** Birthweight >10th percentile	Small for gestational age flag
LGA	SMR02	**1:** Birthweight ≥90th percentile<br>**0:** Birthweight <90th percentile	Large for gestational age flag
Preterm_Delivery	SMR02	**1:** Estimated gestation < 37 weeks<br>**0:** Estimated gestation ≥ 37 weeks and ≤ 42 weeks<br>**Missing:** otherwise	http://www.ndc.scot.nhs.uk/Dictionary-A-Z/Definitions/index.asp?Search=E&ID=242&Title=Estimated Gestation
Postterm_Delivery	SMR02	**1:** Estimated gestation > 42 weeks<br>**0:** Estimated gestation ≥ 37 weeks and ≤ 42 weeks<br>**Missing:** otherwise	http://www.ndc.scot.nhs.uk/Dictionary-A-Z/Definitions/index.asp?Search=E&ID=242&Title=Estimated Gestation
Apgar_Score	SMR02	**1:** Apgar score at 5 mins < 7<br>**0:** Apgar score at 5 mins ≥ 7	http://www.ndc.scot.nhs.uk/Dictionary-A-Z/Definitions/index.asp?Search=A&ID=88&Title=Apgar Score - Babies 1 to 3
Maternal_Obesity	SMR02	**1:** Obese status<br>**0:** Overweight, Healthy or underweight status	Adults with BMI ≥ 30 classed as obese. BMI of girls aged 2 - 19 years old standardized using UK1990 growth reference values and z-score ≥ 6/3 (98th centile) classed as obese.

For peer review only

Maternal_Overweight_Obesity	SMR02	**1:** Overweight or Obese status<br>**0:** Healthy or underweight status	Adults with BMI ≥ 25 classed as overweight or obese. BMI of girls aged 2 - 19 years old standardized using UK1990 growth reference values and z-score ≥ 4/3 (91st centile) classed as overweight or obese.
Age	SMR02	Age of mother at delivery (in years)	
Parity	SMR02	Total number of previous pregnancies	http://www.ndc.scot.nhs.uk/Dictionary-A-Z/Definitions/index.asp?Search=P&ID=409&Title=Previous Pregnancies
Deprivation	SMR02	Carstairs 2001 quintiles for Scotland	1=least deprived; 5=most deprived
Smoking_Status	SMR02	**1:** Yes<br>**0:** No<br>**Missing:** Not known	http://www.ndc.scot.nhs.uk/Dictionary-A-Z/Definitions/index.asp?Search=S&ID=456&Title=Smoker During Pregnancy
Multiple_births	SMR02	**1:** More than one birth this pregnancy<br>**0:** Single birth	http://www.ndc.scot.nhs.uk/Dictionary-A-Z/Definitions/index.asp?Search=N&ID=349&Title=Number of Births this Pregnancy
Multiple_births_in_NRS	NRS Births	**1:** Multiple babies found for this mother's delivery in NRS Births	Multiple babies recorded in NRS Births but only a single baby recorded in SMR02
Previous_Caesarean_section	SMR02	**1:** More than zero<br>**0:** Zero	http://www.ndc.scot.nhs.uk/Dictionary-A-Z/Definitions/index.asp?Search=P&ID=406&Title=Previous Caesarean Sections

For peer review only

STROBE (Strengthening The Reporting of OBservational Studies in Epidemiology) Checklist

A checklist of items that should be included in reports of observational studies. You must report the page number in your manuscript where you consider each of the items listed in this checklist. If you have not included this information, either revise your manuscript accordingly before submitting or note N/A.

Note: An Explanation and Elaboration article discusses each checklist item and gives methodological background and published examples of transparent reporting. The STROBE checklist is best used in conjunction with this article (freely available on the Web sites of PLoS Medicine at http://www.plosmedicine.org/, Annals of Internal Medicine at http://www.annals.org/, and Epidemiology at http://www.epidem.com/). Information on the STROBE Initiative is available at www.strobe-statement.org.

Section and Item	Item No.	Recommendation	Reported on Page No.
**Title and Abstract**	1	(*a*) Indicate the study's design with a commonly used term in the title or the abstract	1
		(*b*) Provide in the abstract an informative and balanced summary of what was done and what was found	2
**Introduction**			
Background/Rationale	2	Explain the scientific background and rationale for the investigation being reported	4,5
Objectives	3	State specific objectives, including any prespecified hypotheses	5
**Methods**			
Study Design	4	Present key elements of study design early in the paper	5
Setting	5	Describe the setting, locations, and relevant dates, including periods of recruitment, exposure, follow-up, and data collection	5,6,7
Participants	6	(*a*) *Cohort study*—Give the eligibility criteria, and the sources and methods of selection of participants. Describe methods of follow-up	5,6,7
		Case-control study—Give the eligibility criteria, and the sources and methods of case ascertainment and control selection. Give the rationale for the choice of cases and controls	N/A
		Cross-sectional study—Give the eligibility criteria, and the sources and methods of selection of participants	N/A
		(*b*) *Cohort study*—For matched studies, give matching criteria and number of exposed and unexposed	N/A
		Case-control study—For matched studies, give matching criteria and the number of controls per case	N/A
Variables	7	Clearly define all outcomes, exposures, predictors, potential confounders, and effect modifiers. Give diagnostic criteria, if applicable	6,7

For peer review only

Section and Item	Item No.	Recommendation	Reported on Page No.
Data Sources/ Measurement	8*	For each variable of interest, give sources of data and details of methods of assessment (measurement). Describe comparability of assessment methods if there is more than one group	5,6,7
Bias	9	Describe any efforts to address potential sources of bias	8,9
Study Size	10	Explain how the study size was arrived at	5
Quantitative Variables	11	Explain how quantitative variables were handled in the analyses. If applicable, describe which groupings were chosen and why	8,9
Statistical Methods	12	(*a*) Describe all statistical methods, including those used to control for confounding	8,9
		(*b*) Describe any methods used to examine subgroups and interactions	N/A
		(*c*) Explain how missing data were addressed	7,16
		(*d*) *Cohort study*—If applicable, explain how loss to follow-up was addressed	N/A
		Case-control study—If applicable, explain how matching of cases and controls was addressed	N/A
		Cross-sectional study—If applicable, describe analytical methods taking account of sampling strategy	N/A
		(*e*) Describe any sensitivity analyses	N/A
**Results**			
Participants	13*	(a) Report numbers of individuals at each stage of study—eg numbers potentially eligible, examined for eligibility, confirmed eligible, included in the study, completing follow-up, and analysed	11
		(b) Give reasons for non-participation at each stage	N/A
		(c) Consider use of a flow diagram	Figure 1
Descriptive Data	14*	(a) Give characteristics of study participants (eg demographic, clinical, social) and information on exposures and potential confounders	5,6,7,8
		(b) Indicate number of participants with missing data for each variable of interest	11
		(c) *Cohort study*—Summarise follow-up time (eg, average and total amount)	5,6
Outcome Data	15*	*Cohort study*—Report numbers of outcome events or summary measures over time	6,7
		Case-control study—Report numbers in each exposure category, or summary measures of exposure	N/A
		Cross-sectional study—Report numbers of outcome events or summary measures	N/A

For peer review only

Section and Item	Item No.	Recommendation	Reported on Page No.
Main Results	16	(*a*) Give unadjusted estimates and, if applicable, confounder-adjusted estimates and their precision (eg, 95% confidence interval). Make clear which confounders were adjusted for and why they were included	9,10,11,12,13
		(*b*) Report category boundaries when continuous variables were categorized	8
		(*c*) If relevant, consider translating estimates of relative risk into absolute risk for a meaningful time period	N/A
Other Analyses	17	Report other analyses done—eg analyses of subgroups and interactions, and sensitivity analyses	N/A
**Discussion**			
Key Results	18	Summarise key results with reference to study objectives	13
Limitations	19	Discuss limitations of the study, taking into account sources of potential bias or imprecision. Discuss both direction and magnitude of any potential bias	16
Interpretation	20	Give a cautious overall interpretation of results considering objectives, limitations, multiplicity of analyses, results from similar studies, and other relevant evidence	13,14,15
Generalisability	21	Discuss the generalisability (external validity) of the study results	13
**Other Information**			
Funding	22	Give the source of funding and the role of the funders for the present study and, if applicable, for the original study on which the present article is based	20

*Give information separately for cases and controls in case-control studies and, if applicable, for exposed and unexposed groups in cohort and cross-sectional studies.

Once you have completed this checklist, please save a copy and upload it as part of your submission. DO NOT include this checklist as part of the main manuscript document. It must be uploaded as a separate file.

For peer review only

BMJ Open

A cohort study of high maternal Body Mass Index and the risk of adverse pregnancy and delivery outcomes in Scotland

Journal:	*BMJ Open*
Manuscript ID	bmjopen-2018-026168.R3
Article Type:	Original research
Date Submitted by the Author:	07-Nov-2019
Complete List of Authors:	Doi, Lawrence; University of Edinburgh, Scottish Collaboration for Public Health Research and Policy, School of Health in Social Science<br>Williams, Andrew James; University of Exeter<br>Marryat, Louise; University of Edinburgh, SCPHRP<br>Frank, John; University of Edinburgh, Scottish Collaboration for Public Health Research and Policy
**Primary Subject Heading**:	Obstetrics and gynaecology
Secondary Subject Heading:	Epidemiology, Paediatrics, Public health, Reproductive medicine, Health policy
Keywords:	OBSTETRICS, EPIDEMIOLOGY, PUBLIC HEALTH

SCHOLARONE™
Manuscripts

BMJ

I, the Submitting Author has the right to grant and does grant on behalf of all authors of the Work (as defined in the below author licence), an exclusive licence and/or a non-exclusive licence for contributions from authors who are: i) UK Crown employees; ii) where BMJ has agreed a CC-BY licence shall apply, and/or iii) in accordance with the terms applicable for US Federal Government officers or employees acting as part of their official duties; on a worldwide, perpetual, irrevocable, royalty-free basis to BMJ Publishing Group Ltd ("BMJ") its licensees and where the relevant Journal is co-owned by BMJ to the co-owners of the Journal, to publish the Work in this journal and any other BMJ products and to exploit all rights, as set out in our licence.

The Submitting Author accepts and understands that any supply made under these terms is made by BMJ to the Submitting Author unless you are acting as an employee on behalf of your employer or a postgraduate student of an affiliated institution which is paying any applicable article publishing charge ("APC") for Open Access articles. Where the Submitting Author wishes to make the Work available on an Open Access basis (and intends to pay the relevant APC), the terms of reuse of such Open Access shall be governed by a Creative Commons licence – details of these licences and which Creative Commons licence will apply to this Work are set out in our licence referred to above.

Other than as permitted in any relevant BMJ Author's Self Archiving Policies, I confirm this Work has not been accepted for publication elsewhere, is not being considered for publication elsewhere and does not duplicate material already published. I confirm all authors consent to publication of this Work and authorise the granting of this licence.

For peer review only

A cohort study of high maternal Body Mass Index and the risk of adverse pregnancy and delivery outcomes in Scotland

Lawrence Doi[1]*, Andrew James Williams[2], Louise Marryat[3,4], John Frank[3,5]

*Correspondence: larry.doi@ed.ac.uk

[1]Scottish Collaboration for Public Health Research and Policy, School of Health in Social Science, University of Edinburgh, Doorway 6, Old Medical School, Teviot Place, EH8 9AG, UK.

[2]European Centre for Environment and Human Health, College of Medicine and Health, University of Exeter, Knowledge Spa, Royal Cornwall Hospital, Truro, Cornwall TR1 3HD, UK.

[3]Farr Institute at Scotland, University of Edinburgh, Nine, Edinburgh BioQuarter, 9 Little France Road, Edinburgh EH16 4UX, UK.

[4]Centre for Clinical Brain Sciences, University of Edinburgh, Royal Edinburgh Hospital, Kennedy Tower, Morningside Park, Edinburgh EH10 5HF, UK.

[5]Usher Institute of Population Health Sciences and Informatics, University of Edinburgh, Doorway 1, Old Medical School, Teviot Place, EH8 9AG, UK.

For peer review only

Abstract

Objective: To examine the association between high maternal weight status and complications during pregnancy and delivery.

Setting: Scotland

Participants: Data from 135,860 first time singleton deliveries in Scotland between 2008 and 2015 were used. Women with overweight and obesity were compared with women with normal weight. Associations between maternal body mass index and complications during pregnancy and delivery were evaluated.

Outcome measures: Gestational diabetes, gestational hypertension, pre-eclampsia, placenta praevia, placental abruption, induction of labour, elective and emergency caesarean sections, pre-term delivery, post-term delivery, low Apgar score, small for gestational age and large for gestational age.

Results: In the multivariable models controlling for potential confounders, we found that compared with women with normal weight, the odds of the following outcomes were significantly increased for women with overweight and obesity [overweight adjusted odds ratio; 95% confidence interval, followed by the same for women with obesity]: gestational hypertension [1.61; 1.49-1.74], [2.48; 2.30-2.68]; gestational diabetes [2.14; 1.86-2.46], [8.25; 7.33-9.30]; pre-eclampsia [1.46; 1.32-1.63] [2.07; 1.87-2.29]; labour induction [1.28; 1.23-1.33], [1.69; 1.62-1.76] and emergency caesarean section [1.82; 1.74-1.91], [3.14; 3.00-3.29].

Conclusions: Women with overweight and obesity in Scotland are at greater risk of adverse pregnancy and delivery outcomes. The risk of these conditions increases steadily with increasing body mass index. Health professionals should be

For peer review only

empowered and trained to deliver promising dietary and lifestyle interventions to women at risk of overweight and obesity prior to conception, and control excessive weight gain in pregnancy.

Strengths and limitations of this study

- This study used a large, retrospectively accessed but cohort-structured, national database covering some of the major maternal and neonatal outcomes in Scotland over eight recent years.
- Analysis used whole study population with adjustment for key potential confounders to estimate impact of high maternal-weight status on each outcome.
- All women with BMI of 30 kg/m^2 or more were considered as having obesity; it is likely that differentiating morbid obesity or obesity class II and III from obesity would have generated more precise risk estimates.
- The completeness of the recording of BMI increased during the study period (2008 to 2015) from 69% to 98%. Using data from the earlier years when the BMI was missing more often might have biased the study sample if it was the case that BMI was not missing at random.

For peer review only

Introduction

The increasing global prevalence of overweight and obesity makes it more likely that a growing number of women with high body mass index (BMI) are becoming pregnant. High maternal BMI during pregnancy has immediate implications for pregnancy complications as well as long-term health implications for both women and offspring (1,2). For instance, in terms of pregnancy complications, a systematic review and meta-analysis involving 11 cohort studies found that caesarean delivery risk increased by 50% in pregnant women who were overweight and was more than double for women who were obese compared with women with normal BMI (3). Among women, high BMI during pregnancy could lead to future chronic disease such as diabetes, heart disease and hypertension (4). Surviving offspring are also more prone to long-term obesity, hypertension, coronary heart disease, diabetes, stroke and asthma (4,5).

Both immediate and long-term health implications of high BMI during pregnancy have economic consequences. For example, a recent study examining infant health utilisation and costs on the NHS in the UK of infants born to women with overweight or obesity found that total mean additional resource cost for infants born to women who are overweight was £65.13, and £1138.11 for infants born to women who are obese (6).

Maternal weights are currently high in Scotland: a recent study reported that 31.5% of mothers were overweight and a further 23.6% were affected by obesity (7). A retrospective cohort study using Scottish obstetric data from 2003 to 2010 examined the impact of maternal BMI on clinical complications, inpatient admissions, and additional short-term costs to the NHS in Scotland revealed that maternal BMI

For peer review only - http://bmjopen.bmj.com/site/about/guidelines.xhtml

influences maternal and neonatal morbidity, the number and duration of maternal and neonatal admissions, and health service costs (8). The study also showed that in comparison with women of normal weight, women who were overweight, obese, or severely obese had an increased risk of essential hypertension [1.87 (1.18–2.96), 11.90 (7.18–19.72), and 36.10 (18.33–71.10)], pregnancy-induced hypertension [1.76 (1.60–1.95), 2.98 (2.65–3.36), and 4.48 (3.57–5.63)], gestational diabetes [3.39 (2.30–4.99), 11.90 (7.54–18.79), and 67.40 (37.84–120.03)], emergency caesarean section [1.94 (1.71–2.21), 3.40 (2.91–3.96), and 14.34 (9.38–21.94)], and elective caesarean section [2.06 (1.84–2.30), 4.61 (4.06–5.24), and 17.92 (13.20–24.34)] (8). Smith et al. (9) using data from 187,290 women in Scotland to examine the risk of maternal obesity in early pregnancy and the risk of pre-term delivery in a retrospective cohort study, found that among nulliparous women, the risk of an elective pre-term delivery increased with increasing BMI. The study also observed that 40% of morbidly obese nulliparous women who experienced an elective pre-term delivery had been diagnosed with pre-eclampsia, in contrast with only 2.6% of the remaining study population (9). In the current study, our aim was to use more recent data to examine the associations between high maternal BMI and complications during pregnancy and delivery in Scotland. Understanding of these associations can highlight areas where prevention strategies could be targeted.

Methods

Study population and data sources

This retrospective cohort study used data from 135,860 first time mothers who gave birth to only one child in Scotland between January 2008 and December 2015. The

For peer review only - http://bmjopen.bmj.com/site/about/guidelines.xhtml

women and infants were identified within three electronic medical record databases: the Scottish Morbidity Record (SMR) 01 and 02 and Scottish Birth Record (SBR). SMR01 is generated for patients receiving inpatient or day care in the General or Acute specialties, whilst SMR02 is generated for patients receiving inpatient or day care in the Obstetric Specialties. The SBR records all of a baby's neonatal care in Scotland. The outcome variables are recorded in these databases according the World Health Organization's International Classification of Diseases, Tenth Revision (ICD-10). Further description of the content of these databases is available (10) and in the web-appendix. Approval was obtained from the Public Benefit and Privacy Panel via the national Electronic Data Research and Innovation Service to use the anonymised data collected by these registries.

Patient and public involvement

Patients were not involved in the design, analysis and interpretation of this study.

Exposure variable

More than 80% of pregnant women in Scotland present themselves for antenatal care during the first trimester of their pregnancy. Height and weight are measured by the midwife at the first antenatal visit, typically before 12 weeks of pregnancy. Body mass index (BMI) was calculated using the formula weight (kg)/height (m^2). BMI categories were defined as normal (<25 kg/m^2), overweight (≥25 kg/m^2 to <30 kg/m^2) and obese (≥30 kg/m^2). BMI completeness was 69% in 2008 but this increased gradually to 87% in 2011 when recording of weight and height became mandatory. By 2015 BMI completeness was 98%.

For peer review only - http://bmjopen.bmj.com/site/about/guidelines.xhtml

Outcomes

Outcome measures included were maternal or pregnancy complications such as gestational diabetes, gestational hypertension and pre-eclampsia (high blood pressure and protein in urine). Conditions affecting delivery or delivery complications studied were placenta praevia (when a baby's placenta partially or totally covers the mother's cervix), placental abruption (when the placenta separates early from the uterus before childbirth), induction of labour, caesarean delivery (includes elective and emergency caesarean sections), pre-term delivery (defined as less than 37 weeks of gestation), post-term delivery (more than 42 weeks of gestation), low Apgar score (less than “7” at 5 minutes), small for gestational age (SGA), and large for gestational age (LGA). SGA were infants with birthweight of ≤10th percentile for gestational age according to UK1990 growth reference curve (11,12), and those with LGA were infants with birth weight ≥90th percentile.

Covariates

Maternal age at delivery, smoking during pregnancy and Carstairs 2001 quintiles for socio-economic status in Scotland, based on the postcode of the mother’s residence at birth, were considered as potentially confounding variables and were included as covariates in the adjusted analyses. Table 1 describes the covariates used in this study by maternal weight status among singleton (a pregnancy with one fetus, as opposed to twins or multiples) first-time pregnancies. The data in Table 1 show the numbers of women who had data on age, deprivation and maternal smoking. This means that the total number of women shown is slightly larger than the number of women analysed as there were women missing some outcome data.

For peer review only - http://bmjopen.bmj.com/site/about/guidelines.xhtml

Table 1. Maternal characteristics among normal weight, overweight and obese women⁺ (singleton, first pregnancies)

	Normal* N = 73,130		Overweight* N = 36,992		Obese* N = 25,738	
	**N**	**%**	**N**	**%**	**N**	**%**
**Maternal age (y)**						
20-24	19,874	27.2	9,368	25.3	7,016	27.3
25-29	24,443	33.4	12,174	32.9	8,475	32.9
30-34	20,880	28.6	10,522	28.4	6,922	26.9
35-39	7,933	10.9	4,928	13.3	3,325	12.9
**Carstairs 2001 quintiles for Scotland**						
Q1 (Least deprived)	14,695	20.1	6,785	18.3	3,870	15.0
Q2	13,820	18.9	6,851	18.5	4,639	18.0
Q3	14,581	19.9	7,549	20.4	5,117	19.9
Q4	15,338	21.0	7,994	21.6	6,079	23.6
Q5 (Most deprived)	14,696	20.1	7,813	21.1	6,033	23.4
**Maternal smoking in pregnancy**						
No	62,439	85.4	31,806	86.0	21,604	83.9
Yes	10,691	14.6	5,186	14.0	4,134	16.1

*Maternal weight status at first antenatal visit
⁺Figures show women who had complete data on age, deprivation and maternal smoking

For peer review only

Data analysis

Using Stata 14 (13), logistic regression was used to calculate odds ratios (ORs). BMI groups with overweight and obesity were compared with the normal BMI group (the reference population). A confidence interval (CI) of 95% was produced for all ORs. The analysis of the outcomes proceeded in a systematic approach.

For the first group of outcomes (gestational diabetes, hypertension and pre-eclampsia) the models were adjusted for confounders, and for the two other conditions that were not the dependent variable, as these conditions can co-occur. This approach was taken throughout the analysis, adjusting for conditions,

For peer review only - http://bmjopen.bmj.com/site/about/guidelines.xhtml

which precede or occur contemporaneously with the outcome being examined as the dependent variable.   This was not done when the outcomes could not co-occur such as pre-term and post-term, SGA and LGA, or method of delivery (induction, elective or emergency C-section). Please see the supplemental file 1 for full details of the variables adjusted for in each model.

Results

Within our study population 53.8 % of pregnant women were categorised as normal weight, 27.3% as overweight and 18.9% as obese. The socio-demographic characteristics of the women in the three BMI categories are presented in Table 1. Maternal smoking prevalence was slightly higher among women with obesity than in women with normal weight or overweight. Among the women who were overweight, 21.1% were from the most deprived and 18.3% were from the least deprived group. However, the difference in social deprivation was more marked within women with obesity. Among this group, 23.4% were from the most deprived group whilst 15.0% were from the least deprived group.

Table 2 shows odds ratios (OR) for pregnancy and delivery complications, among women who were overweight or obese. The risk of gestational diabetes, pre-eclampsia and hypertension increased steadily with increasing BMI. Compared to the normal BMI group, the odds ratio of gestational diabetes was 2.14 (95% CI: 1.86-2.46) but among women who were obese the OR increased to 8.25 (95% CI: 7.33 – 9.30). Relative to women who were of normal weight, the adjusted OR of pre-eclampsia for women who were overweight was 1.46 (95% CI: 1.32-1.62), and 2.07

For peer review only

For peer review only - http://bmjopen.bmj.com/site/about/guidelines.xhtml

(95% CI: 1.87-2.29) for women who were obese. The OR of gestational hypertension, compared with women with normal weight, was 1.61 (95% CI: 1.49-1.74) for women with overweight, and 2.48 (95% CI: 2.30-2.68) for women with obesity.

For peer review only

For peer review only - http://bmjopen.bmj.com/site/about/guidelines.xhtml

Table 2. Pregnancy and delivery complications among normal, overweight and obese singleton women

	Total sample	Normal N (%)	Normal Cases (%)	Overweight N (%)	Overweight Cases (%)	Overweight Adjusted OR* (95% CI)	Obese N (%)	Obese Cases (%)	Obese Adjusted OR* (95% CI)
**Conditions occurring during pregnancy**									
Gestational hypertension	132,899	71,538 (53.8)	1,550 (2.2)	36,188 (27.2)	1,239 (3.4)	1.61 (1.49, 1.74)	25,173 (18.9)	1,275 (5.1)	2.48 (2.30, 2.68)
Gestational diabetes	132,899	71,538 (53.8)	377 (0.5)	36,188 (27.2)	418 (1.2)	2.14 (1.86, 2.46)	25,173 (18.9)	1,082 (4.3)	8.25 (7.33, 9.30)
Pre-eclampsia	132,899	71,538 (53.8)	906 (1.3)	36,188 (27.2)	664 (1.8)	1.46 (1.32, 1.62)	25,173 (18.9)	640 (2.5)	2.07 (1.87, 2.29)
**Conditions affecting delivery**									
Placenta praevia[1]	132,212	71,172 (53.8)	102 (0.1)	36,001 (27.2)	66 (0.2)	1.23 (0.90, 1.68)	25,039 (18.9)	30 (0.1)	0.81 (0.54, 1.22)
Placental abruption[1]	132,212	71,172 (53.8)	133 (0.2)	36,001 (27.2)	55 (0.2)	0.81 (0.59, 1.11)	25,039 (18.9)	38 (0.2)	0.76 (0.53, 1.10)
Small for gestational age[1]	94,906	53,320 (56.2)	6,664 (12.5)	25,122 (26.5)	2,645 (10.5)	0.81 (0.78, 0.85)	16,464 (17.4)	1,726 (10.5)	0.79 (0.74, 0.83)
Large for gestational age[1]	121,177	64,508 (53.2)	17,852 (27.7)	33,356 (27.5)	10,879 (32.6)	1.27 (1.23, 1.30)	23,313 (19.2)	8,575 (36.8)	1.53 (1.48, 1.58)
Pre-term	132,212	71,172 (53.8)	4,295 (6.0)	36,001 (27.2)	2,231 (6.2)	1.02 (0.96, 1.07)	25,039 (18.9)	1,725 (6.9)	1.11 (1.05, 1.18)
Post-term[2]	78,074	43,486 (55.7)	31 (0.1)	20,973 (26.9)	24 (0.1)	1.57 (0.93, 2.68)	13,615(17.4)	14 (0.1)	1.47 (0.78, 2.77)
**Delivery**									
Induction of labour	92,967	53,617 (57.7)	13,417 (25.0)	24,342 (26.2)	7,420 (30.5)	1.28 (1.23, 1.33)	15,008 (16.1)	5,712 (38.1)	1.69 (1.62, 1.76)
Caesarean section	90,183	51,798 (57.4)	11,598 (22.4)	23,827 (26.4)	6,905 (29.0)	1.34 (1.29, 1.39)	14,558 (16.1)	5.262 (36.2)	1.80 (1.73, 1.88)
Emergency caesarean section	80,938	45,715 (56.5)	5,515 (12.1)	21,397 (26.4)	4,475 (20.9)	1.82 (1.74, 1.91)	13,826 (17.1)	4,530 (32.8)	3.14 (3.00, 3.29)
Apgar score	129,773	70,012 (54.0)	12,583 (18.0)	35,307 (27.2)	6,125 (17.4)	0.95 (0.92, 0.99)	24,454 (18.8)	4,342 (17.8)	0.96 (0.93, 1.00)

OR = Odd Ratio; CI = Confidence Interval
*Adjusted for maternal age, deprivation, smoking in pregnancy and the pre- or co-existing conditions (see supplemental file 2)
[1]70 post-term births were excluded from these models as none of them experienced the outcome being estimated
[2]No participants in the study delivering post-term had placenta praevia, placental abruption or had small or large for gestational age babies; therefore 45,957 participants were dropped from this analysis

For peer review only - http://bmjopen.bmj.com/site/about/guidelines.xhtml

Regarding conditions affecting delivery, the odds ratio of placenta praevia was not statistically significant different for both women who were overweight (OR 1.23, 95%CI: 0.90-1.68) or obese (OR 0.81, 95%CI: 0.54-1.22), compared with women with normal weight. The odds ratio of experiencing placental abruption was also not statistically significantly different across the different BMI categories.

In contrast with the normal BMI group, births to women who were overweight and obese were associated with decreased risk of small-for-gestational age ORs 0.81 (95% CI: 0.78-0.85) and 0.79 (95% CI: 0.74-0.83) respectively. However, the risk of large-for-gestational age newborns increased among women with overweight, OR of 1.27 (95%CI: 1.23-1.30) 1.30 (95%CI: 1.26-1.33) and women with obesity, OR 1.53 (95%CI: 1.48-1.58), compared with women of normal weight. Compared to the normal BMI group, the adjusted odds ratio of pre-term delivery was 1.02 (95% CI: 0.96-1.07), however among women who were obese the risk was 1.11 (95% CI: 1.05-1.18). Relative to women who were of normal weight, the adjusted OR of post-term for women who were overweight was 1.57 (95% CI: 0.93-2.68) and 1.47 (95% CI: 0.78-2.77) for women who were obese.

The odds of induction of labour and caesarean section, either elective or emergency, increased with increasing BMI. Regarding induction of labour, the odds ratios were statistically significant for women with overweight (OR 1.28, 95%CI: 1.23-1.33) and those with obesity (OR 1.69, 95%CI: 1.62-1.76) compared with women with normal weight. Women who were overweight had odds ratios of 1.34 (95% CI: 1.29-1.39) for having an elective Caesarean section and higher ORs (1.82, 95% CI: 1.74-1.91) for undergoing emergency Caesarean section, compared with women of normal weight.

For peer review only - http://bmjopen.bmj.com/site/about/guidelines.xhtml

For peer review only

The corresponding ORs for women with obesity were 1.80 (95% CI: 1.73-1.88) and 3.14 (95% CI: 3.00-3.29). Being overweight or obese was associated with reduced risk of low Apgar score. This was barely statistically significant for women who were overweight (OR 0.95, 95% CI: 0.92-0.99) or obese (OR 0.96, 95% CI: 0.93-1.00).

Discussion

In this large, retrospective cohort study, we found that overweight or obesity during pregnancy increased the risk of several adverse pregnancy and delivery complications. Some earlier studies have also found similar findings. Aside from obesity, we also examined overweight because in most populations, a greater number of women are overweight rather than obese, so it is important to also understand the impact of overweight on pregnancy and neonatal outcomes.

In terms of the association between high maternal BMI and conditions that occur *during* pregnancy, we found that the risk of all the conditions considered (gestational hypertension, gestational diabetes and pre-eclampsia) increased steadily with increasing BMI, which is in line with similar studies (2,14,15). A study compared women of normal weight to women who were morbidly obese (BMI greater than 40), and also found that there was an increased risk of pre-eclampsia (OR 4.82; 95% CI: 4.04-5.74) (14). Our study also found that, aside from heightened pre-eclampsia risk for women with obesity, being overweight was also significantly associated with this outcome, albeit to a lesser degree. A meta-analysis of the association between maternal BMI and the risk of pre-eclampsia showed that the risk doubled with each

For peer review only

5-7 kg/m^2 increase in pre-pregnancy BMI (16). It is evident that the risk of pre-eclampsia increases with the degree of weight gain; therefore preventative strategies should be focussed on getting women, especially those already overweight, to reduce weight prior to conception. Weight loss in pregnancy requires careful management in order to avoid unintended consequences (17). Nevertheless, women often engage with health professionals during pregnancy; therefore dietary and lifestyle interventions such as physical activity, which have been shown by reviews and meta-analyses (17,18) to reduce gestational weight gain and improve outcomes for both mother and baby, could be provided to them.

Generally, rates of Caesarean delivery have increased significantly across many developed countries in recent years (19). Our study found that women with overweight and obesity showed an increased risk of Caesarean delivery (both elective and emergency) compared to normal-weight women, but we note that the overall frequency of Caesarean delivery across our obstetric population seems quite high, compared with a previous Swedish study (14). We also found that women with overweight and obesity are at increased risk of labour induction. A very recent systematic review found that women with obesity are more likely than women with a normal weight to end labour induction with Caesarean delivery (20). Possible reasons could be that Caesarean delivery is probably less risky now, due to advances in medical science, which facilitate accurate monitoring of the progress of labour and the detection of fetal intra-partum conditions (21). It is also possible that health professionals in Scotland are probably intervening early with regards to problems in labour among women with overweight or obesity, in order to reduce fetal distress, and its worst outcomes. Nevertheless, this pattern of very high Caesarean

For peer review only - http://bmjopen.bmj.com/site/about/guidelines.xhtml

rates is quite concerning for Scotland, which has invested in programmes aimed at promoting natural birth, such as Keeping Childbirth Natural and Dynamic (KCND). The KCND is a maternity care programme introduced by the Scottish Government with the aim of maximising opportunities for women to have as natural a birth experience as possible, reduce unnecessary interventions in low-risk pregnancy and childbirth, and to provide women-centred care (22,23,24). The early intervention in pregnancy may also explain the reduced risk of low Apgar score for infants born to women with overweight and obesity. It is likely that these women are may receive increased monitoring, which means issues can be identified and managed earlier, to reduce any fetal distress in labour.

Adiposity increases risk of large-for-gestational age and macrosomia (25). In this study, we found that births to women with overweight and obesity were associated with an increased risk of large-for-gestational age infants compared with women of normal weight. Excess weight in pregnancy may shift the entire birth weight distribution upwards, perhaps through hormonal mechanisms that operate at lower levels than in full-blown cases of macrosomia in infants of diabetic mothers. It is therefore unsurprising that high maternal BMI significantly decreased the risk of small-for-gestational age among our study population.

We found that pregnant women with obesity were at significantly increased risk of pre-term delivery, however the risk was high but not statistically significant for women with overweight. A systematic review examining the effect of maternal overweight and obesity on pre-term delivery showed that both women with overweight and obesity were at significantly higher risk of pre-term delivery (26). It

For peer review only - http://bmjopen.bmj.com/site/about/guidelines.xhtml

has been shown that pre-eclampsia leads to pre-term delivery, especially in elective pre-term delivery (27). It is not clear why the relative risk of pre-term delivery for women with overweight in our study population was not statistically significant. However, it is likely that the higher risk of pre-eclampsia in women with obesity, compared with women with overweight, could explain this finding. Regarding post-term delivery, there were no statistically significant relative risks among women with both overweight and obesity. As discussed previously, it is likely that early intervention in pregnancy among our study population reduced the risk of the occurrence of post-term delivery.

We examined the association between high maternal BMI and placental abruption and placenta praevia, but found no statistically significant association between each of these two outcomes and overweight or obesity. This finding is congruent with a previous study (14). It appears that the relationships between maternal overweight and obesity, and both placental abruption and placenta praevia, may require further attention in future research.

Strengths and limitations

This study used a large, retrospectively accessed but cohort-structured, national database covering several maternal and neonatal outcomes. The analysis used whole study population with some adjustment for confounders to estimate impact of high maternal-weight status on each outcome. We restricted the analysis to only single births and first pregnancies to ensure that the births in the sample are relatively independent. The dataset we were provided with combined underweight and normal weight women as normal BMI group. Using this as the reference group

For peer review only - http://bmjopen.bmj.com/site/about/guidelines.xhtml

might have strengthened the association between high maternal BMI and the pregnancy and neonatal outcomes considered. However, only a very small number of women are underweight during pregnancy in Scotland in recent years. Also, some studies differentiate between different obesity categories, but in this study the dataset we accessed did not differentiate these categories, and it was not possible to do this retrospectively; therefore all women with BMI of 30 or more were considered as having obesity. It is likely that differentiating morbid obesity, or obesity class II and III from women with obesity, would have generated additional insight, in the form of a full “dose response relationship”. The completeness of the recording of BMI increased during the study period (2008 to 2015) from 69% to 98%. Using data from the earlier years, when the BMI was missing more often, might have biased the study sample if BMI was not missing at random. The study included control for a limited set of confounders, due to data availability; inclusion of other relevant confounders could have strengthened the analysis. For example, variables such as ethnicity, previous caesarean sections, and time of birth were not available in the dataset which we accessed. Also, we could not analyse neonatal outcomes such as stillbirth, neonatal death and congenital anomaly because these outcomes are not completely ascertained in the dataset we used.

Conclusion

This study has shown that women who were overweight, and especially those who are obese in Scotland are at greater risk of several pregnancy and delivery complications including gestational hypertension, gestational diabetes, pre-eclampsia, labour induction and Caesarean delivery. The risk of these conditions increases steadily with increasing BMI. Health professionals should be empowered

For peer review only - http://bmjopen.bmj.com/site/about/guidelines.xhtml

and trained to deliver promising dietary and lifestyle interventions to women at risk of overweight and obesity prior to conception, and control excessive weight gain in pregnancy.

References

1. Rooney BL, Schauberger CW. Excess pregnancy weight gain and long-term obesity: one decade later. *Obstet Gynecol* 2002; **100(2):** 245-252.
2. Kumari P, Gupta M, Kahlon P, Malviya S. Association between high maternal body mass index and fetomaternal Outcome. *J Obes Metab Res* 2014; **1:** 143-148.
3. Poobalan AS, Aucott LS, Gurung T, Smith WC, Bhattacharya S. Obesity as an independent risk factor for elective and emergency caesarean delivery in nulliparous women – systematic review and meta-analysis of cohort studies. *Obesity Reviews* 2009 10: 28-35. doi:10.1111/j.1467-789X.2008.00537.x
4. Rahman MM, Abe SK, Kanda M, Narita S, Rahman MS, Bilano V, Ota E, Gilmour S, Shibuya K. Maternal body mass index and the risk of birth and maternal health outcomes in low- and middle-income countries: a systematic review and meta-analysis. *Obesity Reviews* 2015; **16:** 758–770.
5. Godfrey KM, Reynolds RM, Prescott SL, Nyirenda M, Jaddoe VWV, Eriksson JG, Broekmann BFP. Influence of maternal obesity on the long-term health of offspring. *Lancet Diabetes Endocrinol* 2017; **5**: 53–64.
6. Morgan KL, Rahman MA, Hill RA, et al. Obesity in pregnancy: infant health service utilisation and costs on the NHS. *BMJ Open* 2015;5:e008357. doi:10.1136/bmjopen-2015- 008357.
7. Doi L, Williams AJ, Frank J. How has child growth around adiposity rebound altered in Scotland since 1990 and what are the risk factors for weight gain

For peer review only

using the Growing Up in Scotland birth cohort 1? *BMC Public Health* 2016; **16:** 1081.

8. Denison FC, Norwood P, Bhattacharya S, Duffy A, Mahmood T, Morris C, Raja EA, Norman JE, Lee AJ, Scotland G. Association between maternal body mass index during pregnancy, short-term morbidity, and increased health service costs: a population-based study. *BJOG* 2014; **121**:72–82.
9. Smith GCS, Shah I, Pell JP, Crossley JA, Dobbie R. Maternal Obesity in Early Pregnancy and Risk of Spontaneous and Elective Preterm Deliveries: A Retrospective Cohort Study, *American Journal of Public Health* 2007; **97**(1): 157-162.
10. Information Service Division (2018). Scottish birth record. http://www.ndc.scot.nhs.uk/Data-Dictionary/SMR-Datasets/Scottish-Birth-Record/
11. Cole TJ, Freeman JV, Preece MA. Body mass index reference curves for the UK, 1990. *Arch Dis Child* 1995; **73(1):** 25–29.
12. Freeman JV, Cole TJ, Chinn S, Jones PRM, White EM, Preece MA. Cross-sectional stature and weight reference curves for the UK 1990. *Arch Dis Child* 1995; **73(1):**17–24.
13. StataCorp. Stata statistical software: release 14. College Station: StataCorp LP; 2015.
14. Cedergren MI. Maternal morbid obesity and the risk of adverse pregnancy outcome. *Obstetricians and Gynecologists* 2004; **103:** 219-224.
15. Scott-Pillai R, Spence D, Cardwell C, Hunter A, Holmes V. The impact of body mass index on maternal and neonatal outcomes: a retrospective study in a UK obstetric population, 2004–2011. *BJOG* 2013; **120:** 932–939.

For peer review only - http://bmjopen.bmj.com/site/about/guidelines.xhtml

16. O'Brien TE, Ray JG, Chan WS. Maternal body mass index and the risk of preeclampsia: A systematic overview. *Epidemiology* 2003; **14:** 368-374.

17. Shieh C, Cullen DL, Pike C, Pressler SJ. Intervention strategies for preventing excessive gestational weight gain: systematic review and meta-analysis. *Obesity Reviews* 2018; doi: 10.1111/obr.12691.

18. Thangaratinam S, Rogozinska E, Jolly K, Glinkowski S, Roseboom T, Tomlinson JW, Kunz R, Mol BW, Coomarasamy A, Khan KS. Effects of interventions in pregnancy on maternal weight and obstetric outcomes: Meta-analysis of randomised evidence. *BMJ.* 2012; **344:** e2088.

19. Althabe F, Sosa C, Belizán JM, Gibbons L, Jacquerioz F, Bergel E. Cesarean section rates and maternal and neonatal mortality in low-, medium-, and high-income countries: an ecological study. *Birth* 2006; **33(4):** 270-277.

20. Ellis JA, Brown CM, Barger B. Carlson NS. (2019), Influence of Maternal Obesity on Labor Induction: A Systematic Review and Meta-Analysis. *Journal of Midwifery & Women's Health*, 2019; **64:** 55-67. doi:10.1111/jmwh.12935

21. Rezaie M, Shahoe R, Shahghebi S. The effect of maternal body mass index on the delivery route in nulliparous women. *Journal of Public Health and Epidemiology* 2013; **5(8):** 346-350.

22. Health Improvement Scotland (2009). Pathways for maternity care. http://www.healthcareimprovementscotland.org/default.aspx?page=12536

23. Abhyankar P, Cheyne H, Maxwell M, McCourt C. A realist evaluation of a normal birth programme. *Evidence Based Midwifery* 2013; https://www.rcm.org.uk/learning-and-career/learning-and-research/ebm-articles/a-realist-evaluation-of-a-normal-birth.

For peer review only - http://bmjopen.bmj.com/site/about/guidelines.xhtml

24. Cheyne H, Abhyankar P, McCourt C. Empowering change: realist evaluation of a Scottish Government programme to support normal birth. *Midwifery* 2013; **29:** 1110–1121.

25. Surkan PJ, Hsieh CC, Johansson AL , Dickman PW, Cnattingius S. Reasons for increasing trends in large for gestational age births. *Obstet Gynecol* 2004; **104**:720–6. doi:10.1097/01.AOG.0000141442.59573.cd

26. McDonald SD, Han Z, Mulla S, Beyene J. Overweight and obesity in mothers and risk of preterm birth and low birth weight infants: systematic review and meta-analyses *BMJ* 2010; 341 :c3428.

27. Hendler I, Goldenberg RL, Mercer BM, et al. The Preterm Prediction Study: association between maternal body mass index and spontaneous and indicated pre-term birth. *Am J Obstet Gynecol*. 2005; **192**:882–886.

w only

Funding

This work was funded by the SCPHRP core grant from the Medical Research Council (Grant Number MR/K023209/1) and the Chief Scientist Office of Scotland. AJW is supported by the European Centre for Environment and Human Health, University of Exeter. LM is supported by the Farr Institute @ Scotland, which is supported by a 10-funder consortium: Arthritis Research UK, the British Heart Foundation, Cancer Research UK, the Economic and Social Research Council, the Engineering and Physical Sciences Research Council, the Medical Research Council, the National Institute of Health Research, the National Institute for Social

For peer review only - http://bmjopen.bmj.com/site/about/guidelines.xhtml

Care and Health Research (Welsh Assembly Government), the Chief Scientist Office (Scottish Government Health Directorates), (MRC Grant No: MR/K007017/1). The funders played no role in the conceptualisation or realisation of the research and no role in the decision to submit it for publication.

Competing interest

The authors declare that they have no competing interests.

Data sharing

Data used was categorised as confidential data release by the Electronic Data Research and Innovation Service of the Information Services Division, NHS National Services Scotland.

Acknowledgements

The authors are grateful to the Electronic Data Research and Innovation Service of the Information Services Division, NHS National Services Scotland for providing the data used in this paper.

Authors' contributions

LD, AJW and JF conceived the original idea for the study and obtained the data. AJW led the statistical analysis with support from LD, LM and JF. LD wrote the first draft of the paper and all authors revised successive drafts and approved the final manuscript.

For peer review only - http://bmjopen.bmj.com/site/about/guidelines.xhtml

Supplementary file 1: Description of data fields used in the study

**Variable Name**	**Database Source**	**Variable Description/Values**	**Notes**
Mother_ID	N/A - study specific	Unique mother identifier	1516-0613/' followed by an anonymous identifier
Baby_ID	N/A - study specific	Unique baby identifier	Mother_ID followed by delivery sequence number followed by a baby sequence number. The baby sequence number for multiple babies from same delivery not necessarily in correct order due to missing CHI numbers.
Delivery_Seq_No	N/A - study specific	Delivery sequence number of mother	
Gest_Diabetes	SMR02	**1:** Yes, Gestational diabetes (diagnosed during this pregnancy)<br>**0:** Yes, Pre-existing diabetes (diagnosed before pregnancy) & No (no diabetes during this pregnancy)<br>**Missing:** Yes, Time of diagnosis unknown & Not Known	http://www.ndc.scot.nhs.uk/Dictionary-A-Z/Definitions/index.asp?Search=D&ID=214&Title=Diabetes
Gest_Hypertension	SMR02/SMR01	**1:** ICD 10 code O13.X<br>**0:** All other codes	Flagged codes cover gestational hypertension
Pre_Eclampsia	SMR02/SMR01	**1:** ICD 10 code O14._<br>**0:** All other codes	Flagged codes cover pre-eclampsia
Placental_Abruption	SMR02/SMR01	**1:** ICD 10 code O45._<br>**0:** All other codes	Flagged codes cover placental abruption
Placental_Praevia	SMR02/SMR01	**1:** ICD 10 code O44._<br>**0:** All other codes	Flagged codes cover placenta praevia

For peer review only

Postpartum_Haemorrhage	SMR02/SMR01	**1:** ICD 10 code O72._<br>**0:** All other codes	Flagged codes cover postpartum haemorrhage
Caesarean_Delivery	SMR02	**1:** Elective (planned) caesarean section & Emergency and unspecified caesarean section<br>**0:** All other codes	http://www.ndc.scot.nhs.uk/Dictionary-A-Z/Definitions/index.asp?Search=M&ID=322&Title=Mode of Delivery - Babies 1 to 3
Labour_Induction	SMR02	**1:** 1-8 - Induction of labour codes<br>**0:** 0, None<br>**Missing:** 9, Not known	http://www.ndc.scot.nhs.uk/Dictionary-A-Z/Definitions/index.asp?Search=I&ID=295&Title=Induction of Labour
SGA	SMR02	**1:** Birthweight ≤10th percentile<br>**0:** Birthweight >10th percentile	Small for gestational age flag
LGA	SMR02	**1:** Birthweight ≥90th percentile<br>**0:** Birthweight <90th percentile	Large for gestational age flag
Preterm_Delivery	SMR02	**1:** Estimated gestation < 37 weeks<br>**0:** Estimated gestation ≥ 37 weeks and ≤ 42 weeks<br>**Missing:** otherwise	http://www.ndc.scot.nhs.uk/Dictionary-A-Z/Definitions/index.asp?Search=E&ID=242&Title=Estimated Gestation
Postterm_Delivery	SMR02	**1:** Estimated gestation > 42 weeks<br>**0:** Estimated gestation ≥ 37 weeks and ≤ 42 weeks<br>**Missing:** otherwise	http://www.ndc.scot.nhs.uk/Dictionary-A-Z/Definitions/index.asp?Search=E&ID=242&Title=Estimated Gestation
Apgar_Score	SMR02	**1:** Apgar score at 5 mins < 7<br>**0:** Apgar score at 5 mins ≥ 7	http://www.ndc.scot.nhs.uk/Dictionary-A-Z/Definitions/index.asp?Search=A&ID=88&Title=Apgar Score - Babies 1 to 3
Maternal_Obesity	SMR02	**1:** Obese status<br>**0:** Overweight, Healthy or underweight status	Adults with BMI ≥ 30 classed as obese. BMI of girls aged 2 - 19 years old standardized using UK1990 growth reference values and z-score ≥ 6/3 (98th centile) classed as obese.

For peer review only

Maternal_Overweight_Obesity	SMR02	**1:** Overweight or Obese status<br>**0:** Healthy or underweight status	Adults with BMI ≥ 25 classed as overweight or obese. BMI of girls aged 2 - 19 years old standardized using UK1990 growth reference values and z-score ≥ 4/3 (91st centile) classed as overweight or obese.
Age	SMR02	Age of mother at delivery (in years)	
Parity	SMR02	Total number of previous pregnancies	http://www.ndc.scot.nhs.uk/Dictionary-A-Z/Definitions/index.asp?Search=P&ID=409&Title=Previous Pregnancies
Deprivation	SMR02	Carstairs 2001 quintiles for Scotland	1=least deprived; 5=most deprived
Smoking_Status	SMR02	**1:** Yes<br>**0:** No<br>**Missing:** Not known	http://www.ndc.scot.nhs.uk/Dictionary-A-Z/Definitions/index.asp?Search=S&ID=456&Title=Smoker During Pregnancy
Multiple_births	SMR02	**1:** More than one birth this pregnancy<br>**0:** Single birth	http://www.ndc.scot.nhs.uk/Dictionary-A-Z/Definitions/index.asp?Search=N&ID=349&Title=Number of Births this Pregnancy
Multiple_births_in_NRS	NRS Births	**1:** Multiple babies found for this mother's delivery in NRS Births	Multiple babies recorded in NRS Births but only a single baby recorded in SMR02
Previous_Caesarean_section	SMR02	**1:** More than zero<br>**0:** Zero	http://www.ndc.scot.nhs.uk/Dictionary-A-Z/Definitions/index.asp?Search=P&ID=406&Title=Previous Caesarean Sections

For peer review only

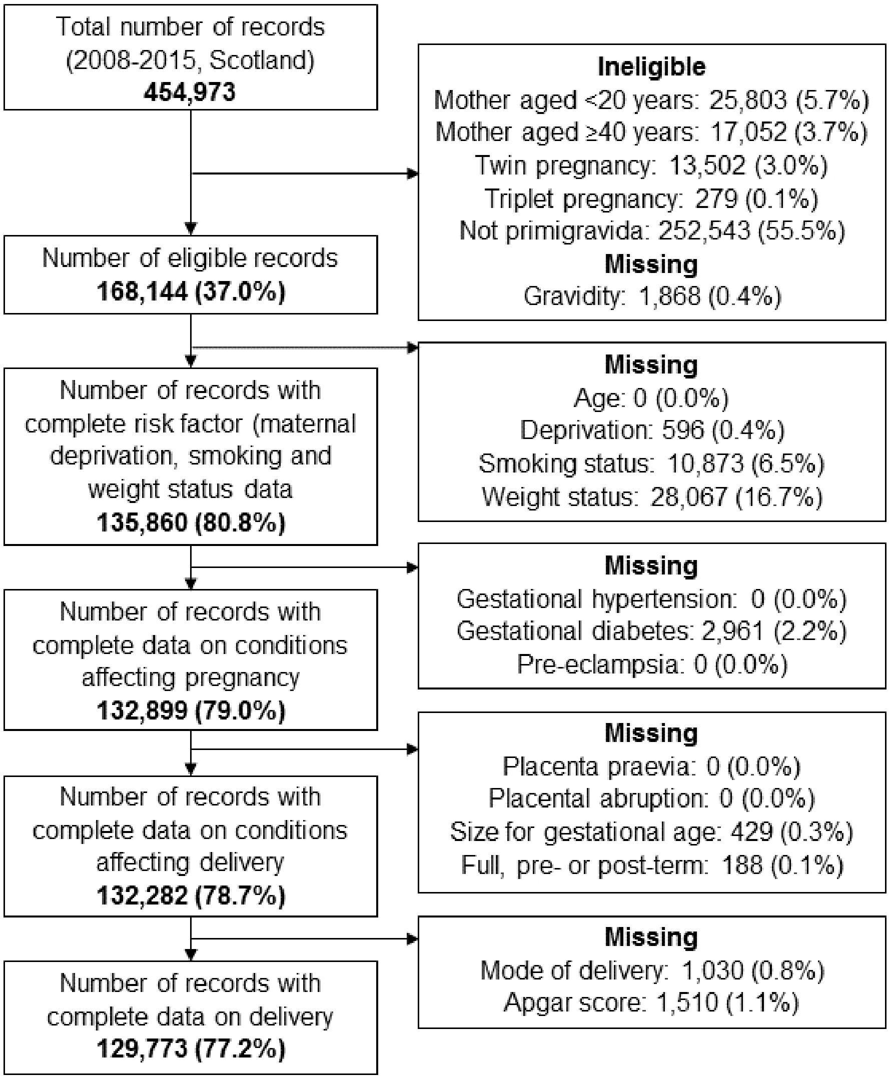
Total number of records
(2008-2015, Scotland)
454,973
Ineligible
Mother aged <20 years: 25,803 (5.7%)
Mother aged ≥40 years: 17,052 (3.7%)
Twin pregnancy: 13,502 (3.0%)
Triplet pregnancy: 279 (0.1%)
Not primigravida: 252,543 (55.5%)
Missing
Gravidity: 1,868 (0.4%)
Number of eligible records
168,144 (37.0%)
Missing
Age: 0 (0.0%)
Deprivation: 596 (0.4%)
Smoking status: 10,873 (6.5%)
Weight status: 28,067 (16.7%)
Number of records with complete risk factor (maternal deprivation, smoking and weight status data
135,860 (80.8%)
Missing
Gestational hypertension: 0 (0.0%)
Gestational diabetes: 2,961 (2.2%)
Pre-eclampsia: 0 (0.0%)
Number of records with complete data on conditions affecting pregnancy
132,899 (79.0%)
Missing
Placenta praevia: 0 (0.0%)
Placental abruption: 0 (0.0%)
Size for gestational age: 429 (0.3%)
Full, pre- or post-term: 188 (0.1%)
Number of records with complete data on conditions affecting delivery
132,282 (78.7%)
Missing
Mode of delivery: 1,030 (0.8%)
Apgar score: 1,510 (1.1%)
Number of records with complete data on delivery
129,773 (77.2%)

STROBE (Strengthening The Reporting of OBservational Studies in Epidemiology) Checklist

A checklist of items that should be included in reports of observational studies. You must report the page number in your manuscript where you consider each of the items listed in this checklist. If you have not included this information, either revise your manuscript accordingly before submitting or note N/A.

Note: An Explanation and Elaboration article discusses each checklist item and gives methodological background and published examples of transparent reporting. The STROBE checklist is best used in conjunction with this article (freely available on the Web sites of PLoS Medicine at http://www.plosmedicine.org/, Annals of Internal Medicine at http://www.annals.org/, and Epidemiology at http://www.epidem.com/). Information on the STROBE Initiative is available at www.strobe-statement.org.

Section and Item	Item No.	Recommendation	Reported on Page No.
**Title and Abstract**	1	(*a*) Indicate the study's design with a commonly used term in the title or the abstract	1
		(*b*) Provide in the abstract an informative and balanced summary of what was done and what was found	2
**Introduction**			
Background/Rationale	2	Explain the scientific background and rationale for the investigation being reported	4,5
Objectives	3	State specific objectives, including any prespecified hypotheses	5
**Methods**			
Study Design	4	Present key elements of study design early in the paper	5
Setting	5	Describe the setting, locations, and relevant dates, including periods of recruitment, exposure, follow-up, and data collection	5,6,7
Participants	6	(*a*) *Cohort study*—Give the eligibility criteria, and the sources and methods of selection of participants. Describe methods of follow-up	5,6,7
		Case-control study—Give the eligibility criteria, and the sources and methods of case ascertainment and control selection. Give the rationale for the choice of cases and controls	N/A
		Cross-sectional study—Give the eligibility criteria, and the sources and methods of selection of participants	N/A
		(*b*) *Cohort study*—For matched studies, give matching criteria and number of exposed and unexposed	N/A
		Case-control study—For matched studies, give matching criteria and the number of controls per case	N/A
Variables	7	Clearly define all outcomes, exposures, predictors, potential confounders, and effect modifiers. Give diagnostic criteria, if applicable	6,7

For peer review only

Section and Item	Item No.	Recommendation	Reported on Page No.
Data Sources/ Measurement	8*	For each variable of interest, give sources of data and details of methods of assessment (measurement). Describe comparability of assessment methods if there is more than one group	5,6,7
Bias	9	Describe any efforts to address potential sources of bias	8,9
Study Size	10	Explain how the study size was arrived at	5
Quantitative Variables	11	Explain how quantitative variables were handled in the analyses. If applicable, describe which groupings were chosen and why	8,9
Statistical Methods	12	(*a*) Describe all statistical methods, including those used to control for confounding	8,9
		(*b*) Describe any methods used to examine subgroups and interactions	N/A
		(*c*) Explain how missing data were addressed	7,16
		(*d*) *Cohort study*—If applicable, explain how loss to follow-up was addressed	N/A
		Case-control study—If applicable, explain how matching of cases and controls was addressed	N/A
		Cross-sectional study—If applicable, describe analytical methods taking account of sampling strategy	N/A
		(*e*) Describe any sensitivity analyses	N/A
**Results**			
Participants	13*	(a) Report numbers of individuals at each stage of study—eg numbers potentially eligible, examined for eligibility, confirmed eligible, included in the study, completing follow-up, and analysed	11
		(b) Give reasons for non-participation at each stage	N/A
		(c) Consider use of a flow diagram	Figure 1
Descriptive Data	14*	(a) Give characteristics of study participants (eg demographic, clinical, social) and information on exposures and potential confounders	5,6,7,8
		(b) Indicate number of participants with missing data for each variable of interest	11
		(c) *Cohort study*—Summarise follow-up time (eg, average and total amount)	5,6
Outcome Data	15*	*Cohort study*—Report numbers of outcome events or summary measures over time	6,7
		Case-control study—Report numbers in each exposure category, or summary measures of exposure	N/A
		Cross-sectional study—Report numbers of outcome events or summary measures	N/A

For peer review only

Section and Item	Item No.	Recommendation	Reported on Page No.
Main Results	16	(*a*) Give unadjusted estimates and, if applicable, confounder-adjusted estimates and their precision (eg, 95% confidence interval). Make clear which confounders were adjusted for and why they were included	9,10,11,12,13
		(*b*) Report category boundaries when continuous variables were categorized	8
		(*c*) If relevant, consider translating estimates of relative risk into absolute risk for a meaningful time period	N/A
Other Analyses	17	Report other analyses done—eg analyses of subgroups and interactions, and sensitivity analyses	N/A
**Discussion**			
Key Results	18	Summarise key results with reference to study objectives	13
Limitations	19	Discuss limitations of the study, taking into account sources of potential bias or imprecision. Discuss both direction and magnitude of any potential bias	16
Interpretation	20	Give a cautious overall interpretation of results considering objectives, limitations, multiplicity of analyses, results from similar studies, and other relevant evidence	13,14,15
Generalisability	21	Discuss the generalisability (external validity) of the study results	13
**Other Information**			
Funding	22	Give the source of funding and the role of the funders for the present study and, if applicable, for the original study on which the present article is based	20

*Give information separately for cases and controls in case-control studies and, if applicable, for exposed and unexposed groups in cohort and cross-sectional studies.

Once you have completed this checklist, please save a copy and upload it as part of your submission. DO NOT include this checklist as part of the main manuscript document. It must be uploaded as a separate file.

For peer review only

BMJ Open

A cohort study of high maternal Body Mass Index and the risk of adverse pregnancy and delivery outcomes in Scotland

Journal:	*BMJ Open*
Manuscript ID	bmjopen-2018-026168.R4
Article Type:	Original research
Date Submitted by the Author:	22-Dec-2019
Complete List of Authors:	Doi, Lawrence; University of Edinburgh, Scottish Collaboration for Public Health Research and Policy, School of Health in Social Science<br>Williams, Andrew James; University of Exeter<br>Marryat, Louise; University of Edinburgh, SCPHRP<br>Frank, John; University of Edinburgh, Scottish Collaboration for Public Health Research and Policy
**Primary Subject Heading**:	Obstetrics and gynaecology
Secondary Subject Heading:	Epidemiology, Paediatrics, Public health, Reproductive medicine, Health policy
Keywords:	OBSTETRICS, EPIDEMIOLOGY, PUBLIC HEALTH

SCHOLARONE™
Manuscripts

I, the Submitting Author has the right to grant and does grant on behalf of all authors of the Work (as defined in the below author licence), an exclusive licence and/or a non-exclusive licence for contributions from authors who are: i) UK Crown employees; ii) where BMJ has agreed a CC-BY licence shall apply, and/or iii) in accordance with the terms applicable for US Federal Government officers or employees acting as part of their official duties; on a worldwide, perpetual, irrevocable, royalty-free basis to BMJ Publishing Group Ltd ("BMJ") its licensees and where the relevant Journal is co-owned by BMJ to the co-owners of the Journal, to publish the Work in this journal and any other BMJ products and to exploit all rights, as set out in our licence.

The Submitting Author accepts and understands that any supply made under these terms is made by BMJ to the Submitting Author unless you are acting as an employee on behalf of your employer or a postgraduate student of an affiliated institution which is paying any applicable article publishing charge ("APC") for Open Access articles. Where the Submitting Author wishes to make the Work available on an Open Access basis (and intends to pay the relevant APC), the terms of reuse of such Open Access shall be governed by a Creative Commons licence – details of these licences and which Creative Commons licence will apply to this Work are set out in our licence referred to above.

Other than as permitted in any relevant BMJ Author's Self Archiving Policies, I confirm this Work has not been accepted for publication elsewhere, is not being considered for publication elsewhere and does not duplicate material already published. I confirm all authors consent to publication of this Work and authorise the granting of this licence.

For peer review only

A cohort study of high maternal Body Mass Index and the risk of adverse pregnancy and delivery outcomes in Scotland

Lawrence Doi[1]*, Andrew James Williams[2], Louise Marryat[3,4], John Frank[3,5]

*Correspondence: larry.doi@ed.ac.uk

[1]Scottish Collaboration for Public Health Research and Policy, School of Health in Social Science, University of Edinburgh, Doorway 6, Old Medical School, Teviot Place, EH8 9AG, UK.

[2]European Centre for Environment and Human Health, University of Exeter Medical School, Knowledge Spa, Royal Cornwall Hospital, Truro, Cornwall TR1 3HD, UK.

[3]Farr Institute at Scotland, University of Edinburgh, Nine, Edinburgh BioQuarter, 9 Little France Road, Edinburgh EH16 4UX, UK.

[4]Centre for Clinical Brain Sciences, University of Edinburgh, Royal Edinburgh Hospital, Kennedy Tower, Morningside Park, Edinburgh EH10 5HF, UK.

[5]Usher Institute of Population Health Sciences and Informatics, University of Edinburgh, Doorway 1, Old Medical School, Teviot Place, EH8 9AG, UK.

For peer review only

For peer review only - http://bmjopen.bmj.com/site/about/guidelines.xhtml

Abstract

Objective: To examine the association between high maternal weight status and complications during pregnancy and delivery.

Setting: Scotland

Participants: Data from 132,899 first time singleton deliveries in Scotland between 2008 and 2015 were used. Women with overweight and obesity were compared with women with normal weight. Associations between maternal body mass index and complications during pregnancy and delivery were evaluated.

Outcome measures: Gestational diabetes, gestational hypertension, pre-eclampsia, placenta praevia, placental abruption, induction of labour, elective and emergency caesarean sections, pre-term delivery, post-term delivery, low Apgar score, small for gestational age and large for gestational age.

Results: In the multivariable models controlling for potential confounders, we found that, compared with women with normal weight, the odds of the following outcomes were significantly increased for women with overweight and obesity [overweight adjusted odds ratio; 95% confidence interval, followed by the same for women with obesity]: gestational hypertension [1.61; 1.49-1.74], [2.48; 2.30-2.68]; gestational diabetes [2.14; 1.86-2.46], [8.25; 7.33-9.30]; pre-eclampsia [1.46; 1.32-1.63] [2.07; 1.87-2.29]; labour induction [1.28; 1.23-1.33], [1.69; 1.62-1.76] and emergency caesarean section [1.82; 1.74-1.91], [3.14; 3.00-3.29].

Conclusions: Women with overweight and obesity in Scotland are at greater odds of adverse pregnancy and delivery outcomes. The odds of these conditions increases with increasing body mass index. Health professionals should be

For peer review only - http://bmjopen.bmj.com/site/about/guidelines.xhtml

empowered and trained to deliver promising dietary and lifestyle interventions to women at risk of overweight and obesity prior to conception, and control excessive weight gain in pregnancy.

Strengths and limitations of this study

- This study used a large, retrospectively accessed but cohort-structured, national database covering some of the major maternal and neonatal outcomes in Scotland over eight recent years.
- Analyses were adjusted for some of the key potential confounders to estimate impact of high maternal-weight status on each outcome.
- All women with BMI of 30 kg/m^2 or more were considered as having obesity; it is likely that differentiating morbid obesity or obesity class II and III from obesity would have generated more precise estimates.
- The completeness of the recording of BMI increased during the study period (2008 to 2015) from 69% to 98%. Using data from the earlier years when the BMI was missing more often might have biased the study sample if it was the case that BMI was not missing at random.

For peer review only

Introduction

The increasing global prevalence of overweight and obesity makes it more likely that a growing number of women with high body mass index (BMI) are becoming pregnant. High maternal BMI during pregnancy has immediate implications for pregnancy complications as well as long-term health implications for both women and offspring (1,2). For instance, in terms of pregnancy complications, a systematic review and meta-analysis involving 11 cohort studies found that caesarean delivery risk increased by 50% in pregnant women who were overweight and was more than double for women who were obese compared with women with normal BMI (3). High BMI during pregnancy could lead to future chronic disease such as diabetes, heart disease and hypertension (4). Surviving offspring are also more prone to long-term obesity, hypertension, coronary heart disease, diabetes, stroke and asthma (4,5). Both immediate and long-term health implications of high BMI during pregnancy have economic consequences. For example, a recent study examining infant health utilisation and costs on the NHS in the UK of infants born to women with overweight or obesity found that total mean additional resource cost for infants born to women who are overweight was £65.13, and £1138.11 for infants born to women who are obese (6).

Maternal weights are currently high in Scotland: a recent study reported that 31.5% of mothers were overweight and a further 23.6% were affected by obesity (7). A retrospective cohort study using Scottish obstetric data from 2003 to 2010 examined the impact of maternal BMI on clinical complications, inpatient admissions, and additional short-term costs to the NHS in Scotland revealed that maternal BMI influences maternal and neonatal morbidity, the number and duration of maternal

For peer review only - http://bmjopen.bmj.com/site/about/guidelines.xhtml

and neonatal admissions, and health service costs (8). The study also showed that in comparison with women of normal weight, women who were overweight, obese, or severely obese had an increased risk of essential hypertension [1.87 (1.18–2.96), 11.90 (7.18–19.72), and 36.10 (18.33–71.10)], pregnancy-induced hypertension [1.76 (1.60–1.95), 2.98 (2.65–3.36), and 4.48 (3.57–5.63)], gestational diabetes [3.39 (2.30–4.99), 11.90 (7.54–18.79), and 67.40 (37.84–120.03)], emergency caesarean section [1.94 (1.71–2.21), 3.40 (2.91–3.96), and 14.34 (9.38–21.94)], and elective caesarean section [2.06 (1.84–2.30), 4.61 (4.06–5.24), and 17.92 (13.20–24.34)] (8). Smith et al. (9), using data from a retrospective cohort study of 187,290 women in Scotland to examine the risk of maternal obesity in early pregnancy and the risk of pre-term delivery, found that among nulliparous women, the risk of an elective pre-term delivery increased with increasing BMI. The study also observed that 40% of morbidly obese nulliparous women who experienced an elective pre-term delivery had been diagnosed with pre-eclampsia, in contrast with only 2.6% of the remaining study population (9). Maternal obesity has also been linked to low Apgar score and pre-term and post-term delivery as well as the risk of intrapartum complications, such as placenta praevia and placental abruption (2,10). It is likely that any risk of intrapartum complications may necessitate labour induction or more frequent caesarean delivery.

In the current study, we hypothesised, based on previous studies elsewhere, that women with obesity and their babies experience higher rates of virtually all perinatal complications, which are routinely collected in Scotland, except perhaps low birth weight (due to the macrosomia effect of overt or covert gestational diabetes), and that women with overweight and their babies experience an excess risk of these

For peer review only - http://bmjopen.bmj.com/site/about/guidelines.xhtml

same outcomes, but one not as high as women with obesity and their offspring. Therefore, our aim was to use more recent data to examine the associations between high maternal BMI and complications during pregnancy and delivery in Scotland. Understanding of these associations can highlight areas where prevention strategies could be targeted.

Methods

Study population and data sources

This retrospective cohort study used data from 132,899 first time mothers who gave birth to only one child in Scotland between January 2008 and December 2015. The women and infants were identified within three electronic medical record databases: the Scottish Morbidity Record (SMR) 01 and 02 and Scottish Birth Record (SBR). SMR01 is generated for patients receiving inpatient or day care in the General or Acute specialties, whilst SMR02 is generated for patients receiving inpatient or day care in the Obstetric Specialties. The SBR records all of a baby's neonatal care in Scotland. Relevant outcome variables are recorded in these databases according to the World Health Organization's International Classification of Diseases, Tenth Revision (ICD-10) or NHS Scotland classifications (11). Further description of the content of these databases is available (12) and in the supplemental file 1.. The study was designed as a clinical audit so did not require approval from a Research Ethics Committee. However, approval was obtained from the Public Benefit and Privacy Panel via the national Electronic Data Research and Innovation Service to use the anonymised data collected by these registries. As a clinical audit making secondary use of anonymised electronic patient records, it was necessary to account

For peer review only - http://bmjopen.bmj.com/site/about/guidelines.xhtml

for missing data which was relevant to this research study (see the supplemental file 2 for the flow diagram illustrating how the final sample size was reached. The large number of variables involved in this study and a low likelihood that missingness was at random meant that imputation methods would have been complicated and a complete case analysis was more suitable for this population-wide study.

Patient and public involvement

Patients were not involved in the design, analyses and interpretation of this study.

Exposure variable

More than 80% of pregnant women in Scotland present themselves for antenatal care during the first trimester of their pregnancy (13). Height and weight are usually measured by the midwife at the first antenatal visit, typically before 12 weeks of pregnancy. Body mass index (BMI) was calculated using the formula weight (kg)/height (m^2). BMI categories were defined as normal (<25 kg/m^2), overweight (≥25 kg/m^2 to <30 kg/m^2) and obese (≥30 kg/m^2). BMI completeness was 69% in 2008 but this increased gradually to 87% in 2011 when recording of weight and height became mandatory. By 2015 BMI completeness was 98%.

Outcomes

Outcome measures included were maternal or pregnancy complications organised into three groups related to when they occur during the pregnancy;

- Conditions affecting pregnancy: gestational diabetes, gestational hypertension and pre-eclampsia (high blood pressure and protein in urine).

For peer review only - http://bmjopen.bmj.com/site/about/guidelines.xhtml

- Conditions affecting delivery: placenta praevia (when a baby's placenta partially or totally covers the mother's cervix), placental abruption (when the placenta separates early from the uterus before childbirth), pre-term delivery (defined as less than 37 weeks of gestation), post-term delivery (more than 42 weeks of gestation), small for gestational age (SGA), and large for gestational age (LGA). SGA were infants with birthweight of ≤10th percentile for gestational age according to UK1990 growth reference curve (14,15), and those with LGA were infants with birth weight ≥90th percentile.
- Delivery: induction of labour, caesarean delivery (includes elective and emergency caesarean sections) and low Apgar score (less than “7” at 5 minutes).

Covariates

Maternal age at delivery, smoking during pregnancy and Carstairs 2001 quintiles for socio-economic status in Scotland, based on the postcode of the mother’s residence at birth, were considered as potentially confounding variables and were included as covariates in the adjusted analyses. Table 1 describes the covariates used in this study by maternal weight status among singleton (a pregnancy with one fetus, as opposed to twins or multiples) first-time pregnancies. The data in Table 1 show the numbers of women who had data on age, deprivation, maternal smoking and the three conditions being studied that occur during pregnancy.

Table 1. Maternal characteristics among normal weight, overweight and obese women[+] (singleton, first pregnancies)

	**Normal*<br>N = 71,538**		**Overweight*<br>N = 36,188**		**Obese*<br>N = 25,173**	
	**N**	**%**	**N**	**%**	**N**	**%**
**Maternal age (y)**						
20-24	19,372	27.1	9,152	25.3	6,851	27.2
25-29	23,871	33.4	11,895	32.9	8,280	32.9

For peer review only - http://bmjopen.bmj.com/site/about/guidelines.xhtml

30-34	20,488	28.6	10,304	28.5	6,777	26.9
35-39	7,807	10.9	4,837	13.4	3,265	13.0
**Carstairs 2001 quintiles for Scotland**						
Q1 (Least deprived)	14,546	20.3	6,715	18.6	3,833	15.2
Q2	13,574	19.0	6,733	18.6	4,578	18.2
Q3	14,245	19.9	7,382	20.4	5,005	19.9
Q4	14,930	20.9	7,777	21.5	5,909	23.5
Q5 (Most deprived)	14,243	19.9	7,581	21.0	5,848	23.2
**Maternal smoking in pregnancy**						
No	61,116	85.4	31,119	86.0	21,130	83.9
Yes	10,422	14.6	5,069	14.0	4,043	16.1

*Maternal weight status at first antenatal visit

+Figures show women who had complete data on age, deprivation, maternal smoking and the conditions occurring during pregnancy

Data analyses

Using Stata 14 (16), logistic regression models were fitted to calculate odds ratios (ORs). BMI groups with overweight and obesity were compared with the normal BMI group (the reference population). A confidence interval (CI) of 95% was produced for all ORs. The analyses of the outcomes proceeded in a systematic approach.

The outcomes were analysed in the three groups described above. As some of the outcomes were mutually exclusive (e.g. a baby cannot be both small and large for gestational age) those with the opposing outcome were excluded from the outcome being analysed. Each model was also adjusted for any of the outcomes that occurred earlier in the pregnancy. Table 2 provides information on the covariates adjusted for in each model.

For peer review only

Table 2. Full list of variables adjusted for in each of the models in Table 3

	Risk factors			
	**Maternal circumstances**	**Conditions affecting pregnancy**	**Conditions affecting delivery**	**Delivery**
**Conditions affecting pregnancy**				
Gestational hypertension	• Age<br>• Deprivation<br>• Smoking status<br>• Weight status	• Gestational diabetes<br>• Pre-eclampsia	-	-
Gestational diabetes	• Age<br>• Deprivation<br>• Smoking status<br>• Weight status	• Gestational hypertension<br>• Pre-eclampsia	-	-
Pre-eclampsia	• Age<br>• Deprivation<br>• Smoking status<br>• Weight status	• Gestational hypertension<br>• Gestational diabetes	-	-
**Conditions affecting delivery**				
Placenta praevia	• Age<br>• Deprivation<br>• Smoking status<br>• Weight status	• Gestational hypertension<br>• Gestational diabetes<br>• Pre-eclampsia	• Placental abruption<br>• Size for gestational age<br>• Full, pre- or post-term	-
Placental abruption	• Age<br>• Deprivation<br>• Smoking status<br>• Weight status	• Gestational hypertension<br>• Gestational diabetes<br>• Pre-eclampsia	• Placenta praevia<br>• Size for gestational age<br>• Full, pre- or post-term	-
Small for gestational age	• Age<br>• Deprivation<br>• Smoking status<br>• Weight status	• Gestational hypertension<br>• Gestational diabetes<br>• Pre-eclampsia	• Placental abruption<br>• Placenta praevia<br>• Full, pre- or post-term	-
Large for gestational age	• Age<br>• Deprivation<br>• Smoking status<br>• Weight status	• Gestational hypertension<br>• Gestational diabetes<br>• Pre-eclampsia	• Placental abruption<br>• Placenta praevia<br>• Full, pre- or post-term	-
Pre-term	• Age<br>• Deprivation<br>• Smoking status	• Gestational hypertension<br>• Gestational diabetes<br>• Pre-eclampsia	• Placental abruption<br>• Placenta praevia<br>• Size for gestational age	-

For peer review only

For peer review only - http://bmjopen.bmj.com/site/about/guidelines.xhtml

	• Weight status			
Post-term	• Age<br>• Deprivation<br>• Smoking status<br>• Weight status	• Gestational hypertension<br>• Gestational diabetes<br>• Pre-eclampsia	• Placental abruption<br>• Placenta praevia<br>• Size for gestational age	-
**Delivery**				
Induction of labour	• Age<br>• Deprivation<br>• Smoking status<br>• Weight status	• Gestational hypertension<br>• Gestational diabetes<br>• Pre-eclampsia	• Placental abruption<br>• Placenta praevia<br>• Size for gestational age<br>• Full, pre- or post-term	-
Caesarean section	• Age<br>• Deprivation<br>• Smoking status<br>• Weight status	• Gestational hypertension<br>• Gestational diabetes<br>• Pre-eclampsia	• Placental abruption<br>• Placenta praevia<br>• Size for gestational age<br>• Full, pre- or post-term	-
Emergency caesarean section	• Age<br>• Deprivation<br>• Smoking status<br>• Weight status	• Gestational hypertension<br>• Gestational diabetes<br>• Pre-eclampsia	• Placental abruption<br>• Placenta praevia<br>• Size for gestational age<br>• Full, pre- or post-term	-
Apgar score	• Age<br>• Deprivation<br>• Smoking status<br>• Weight status	• Gestational hypertension<br>• Gestational diabetes<br>• Pre-eclampsia	• Placental abruption<br>• Placenta praevia<br>• Size for gestational age<br>• Full, pre- or post-term	• Mode of delivery

For peer review only - http://bmjopen.bmj.com/site/about/guidelines.xhtml

Results

Within our study population 53.8% of pregnant women were categorised as normal weight, 27.2% as overweight and 18.9% as obese. The socio-demographic characteristics of the women in the three BMI categories are presented in Table 1. Maternal smoking prevalence was slightly higher among women with obesity than in women with normal weight or overweight. Among the women who were overweight, 21.0% were from the most deprived and 18.6% were from the least deprived group. However, the difference in social deprivation was more marked within women with obesity. Among this group, 23.2% were from the most deprived group whilst 15.2% were from the least deprived group.

Table 3 shows odds ratios (OR) for pregnancy and delivery complications, among women who were overweight or obese. The odds of gestational diabetes, pre-eclampsia and hypertension increased steadily with increasing BMI. Compared to the normal BMI group, the odds ratio of gestational diabetes was 2.14 (95% CI: 1.86-2.46) but among women who were obese the OR increased to 8.25 (95% CI: 7.33 – 9.30). Relative to women who were of normal weight, the adjusted OR of pre-eclampsia for women who were overweight was 1.46 (95% CI: 1.32-1.62), and 2.07 (95% CI: 1.87-2.29) for women who were obese. The OR of gestational hypertension, compared with women with normal weight, was 1.61 (95% CI: 1.49-1.74) for women with overweight, and 2.48 (95% CI: 2.30-2.68) for women with obesity.

For peer review only - http://bmjopen.bmj.com/site/about/guidelines.xhtml

Table 3. Pregnancy and delivery complications among normal, overweight and obese singleton women

	Total	Normal		Overweight			Obese		
	sample	N (%)	Cases (%)	N (%)	Cases (%)	Adjusted OR* (95% CI)	N (%)	Cases (%)	Adjusted OR* (95% CI)
**Conditions affecting pregnancy**									
Gestational hypertension	132,899	71,538 (53.8)	1,550 (2.2)	36,188 (27.2)	1,239 (3.4)	1.61 (1.49, 1.74)	25,173 (18.9)	1,275 (5.1)	2.48 (2.30, 2.68)
Gestational diabetes	132,899	71,538 (53.8)	377 (0.5)	36,188 (27.2)	418 (1.2)	2.14 (1.86, 2.46)	25,173 (18.9)	1,082 (4.3)	8.25 (7.33, 9.30)
Pre-eclampsia	132,899	71,538 (53.8)	906 (1.3)	36,188 (27.2)	664 (1.8)	1.46 (1.32, 1.62)	25,173 (18.9)	640 (2.5)	2.07 (1.87, 2.29)
**Conditions affecting delivery**									
Placenta praevia[1]	132,212	71,172 (53.8)	102 (0.1)	36,001 (27.2)	66 (0.2)	1.23 (0.90, 1.68)	25,039 (18.9)	30 (0.1)	0.81 (0.54, 1.22)
Placental abruption[1]	132,212	71,172 (53.8)	133 (0.2)	36,001 (27.2)	55 (0.2)	0.81 (0.59, 1.11)	25,039 (18.9)	38 (0.2)	0.76 (0.53, 1.10)
Small for gestational age[1,2]	94,906	53,320 (56.2)	6,664 (12.5)	25,122 (26.5)	2,645 (10.5)	0.81 (0.78, 0.85)	16,464 (17.4)	1,726 (10.5)	0.79 (0.74, 0.83)
Large for gestational age[1,2]	121,177	64,508 (53.2)	17,852 (27.7)	33,356 (27.5)	10,879 (32.6)	1.27 (1.23, 1.30)	23,313 (19.2)	8,575 (36.8)	1.53 (1.48, 1.58)
Pre-term[1,2]	132,212	71,172 (53.8)	4,295 (6.0)	36,001 (27.2)	2,231 (6.2)	1.02 (0.96, 1.07)	25,039 (18.9)	1,725 (6.9)	1.11 (1.05, 1.18)
Post-term[1,2,3]	78,074	43,486 (55.7)	31 (0.1)	20,973 (26.9)	24 (0.1)	1.57 (0.93, 2.68)	13,615(17.4)	14 (0.1)	1.47 (0.78, 2.77)
**Delivery**									
Induction of labour[2]	92,967	53,617 (57.7)	13,417 (25.0)	24,342 (26.2)	7,420 (30.5)	1.28 (1.23, 1.33)	15,008 (16.1)	5,712 (38.1)	1.69 (1.62, 1.76)
Caesarean section[2]	90,183	51,798 (57.4)	11,598 (22.4)	23,827 (26.4)	6,905 (29.0)	1.34 (1.29, 1.39)	14,558 (16.1)	5.262 (36.2)	1.80 (1.73, 1.88)
Emergency caesarean section[2]	80,938	45,715 (56.5)	5,515 (12.1)	21,397 (26.4)	4,475 (20.9)	1.82 (1.74, 1.91)	13,826 (17.1)	4,530 (32.8)	3.14 (3.00, 3.29)
Apgar score	129,773	70,012 (54.0)	12,583 (18.0)	35,307 (27.2)	6,125 (17.4)	0.95 (0.92, 0.99)	24,454 (18.8)	4,342 (17.8)	0.96 (0.93, 1.00)

OR = Odds Ratio; CI = Confidence Interval
*Adjusted for maternal age, deprivation, smoking in pregnancy and the pre- or co-existing conditions (see supplemental file 2)
[1]70 post-term births were excluded from these models as none of them experienced the outcome being estimated
[2]These total sample sizes differ as the outcome being estimated is mutually exclusive from one or more of the other outcomes within that group. For example, any baby being delivered pre-term cannot also have been delivered post-term and therefore these two models include the same 'controls' those delivered at term but difference 'cases' pre-term or post-term.
[3]No participants in the study delivering post-term had placenta praevia, placental abruption or had small or large for gestational age babies; therefore 45,957 participants were dropped from this analyses

For peer review only - http://bmjopen.bmj.com/site/about/guidelines.xhtml

Regarding conditions affecting delivery, the odds ratio of placenta praevia was not statistically significant different for both women who were overweight (OR 1.23, 95%CI: 0.90-1.68) or obese (OR 0.81, 95%CI: 0.54-1.22), compared with women with normal weight. The odds ratio of experiencing placental abruption was also not statistically significantly different across the different BMI categories.

In contrast with the normal BMI group, births to women who were overweight and obese were associated with decreased odds of small-for-gestational age ORs 0.81 (95% CI: 0.78-0.85) and 0.79 (95% CI: 0.74-0.83) respectively. However, the odds of large-for-gestational age newborns increased among women with overweight, OR of 1.27 (95%CI: 1.23-1.30) and women with obesity, OR 1.53 (95%CI: 1.48-1.58), compared with women of normal weight. Regarding the odds ratios for the pre-term and post-term outcomes, only the pre-term outcome for the obese group was statistically significant and the others were not significant: compared to the normal BMI group, the adjusted odds ratio of pre-term delivery was 1.02 (95% CI: 0.96-1.07), however among women who were obese the odds ratio was 1.11 (95% CI: 1.05-1.18). Relative to women who were of normal weight, the adjusted OR of post-term for women who were overweight was 1.57 (95% CI: 0.93-2.68) and 1.47 (95% CI: 0.78-2.77) for women who were obese.

The odds of induction of labour and caesarean section, either elective or emergency, increased with increasing BMI. Regarding induction of labour, the odds ratios were statistically significant for women with overweight (OR 1.28, 95%CI: 1.23-1.33) and those with obesity (OR 1.69, 95%CI: 1.62-1.76) compared with women with normal weight. Women who were overweight had odds ratios of 1.34 (95% CI: 1.29-1.39) for

For peer review only - http://bmjopen.bmj.com/site/about/guidelines.xhtml

having an elective Caesarean section and higher odds ratios (1.82, 95% CI: 1.74-1.91) for undergoing emergency Caesarean section, compared with women of normal weight. The corresponding odds ratios for women with obesity were 1.80 (95% CI: 1.73-1.88) and 3.14 (95% CI: 3.00-3.29). Being overweight or obese was associated with reduced odds of low Apgar score. This was barely statistically significant for women who were overweight (OR 0.95, 95% CI: 0.92-0.99) or obese (OR 0.96, 95% CI: 0.93-1.00).

Discussion

In this large, retrospective cohort study, we found that overweight or obesity during pregnancy was associated with increased odds of several adverse pregnancy and delivery complications. Aside from obesity, we also examined overweight because in most populations, a greater number of women are overweight rather than obese, so it is important to also understand the impact of overweight on pregnancy and neonatal outcomes.

In terms of the associations between high maternal BMI and conditions that occur *during* pregnancy, we found that the odds of all the conditions considered (gestational hypertension, gestational diabetes and pre-eclampsia) increased steadily with increasing BMI, which is in line with similar studies (2,10,17). A study compared women of normal weight to women who were morbidly obese (BMI greater than 40), and also found that there was an increased odds of pre-eclampsia (OR 4.82; 95% CI: 4.04-5.74) (10). Our study also found that, aside from heightened

For peer review only - http://bmjopen.bmj.com/site/about/guidelines.xhtml

pre-eclampsia odds for women with obesity, being overweight was also significantly associated with this outcome, albeit to a lesser degree. A meta-analysis of the association between maternal BMI and the risk of pre-eclampsia showed that the risk doubled with each 5-7 kg/m^2 increase in pre-pregnancy BMI (18). It is evident that the risk of pre-eclampsia increases with the degree of weight gain; therefore preventative strategies should be focussed on getting women, especially those already overweight, to reduce weight prior to conception. Weight loss in pregnancy requires careful management in order to avoid unintended consequences (19). Nevertheless, women often engage with health professionals during pregnancy; therefore dietary and lifestyle interventions such as physical activity, which have been shown by reviews and meta-analyses (19,20) to reduce gestational weight gain and improve outcomes for both mother and baby, could be provided to them.

Generally, rates of Caesarean delivery have increased significantly across many developed countries in recent years (21). Our study found that women with overweight and obesity showed increased odds of Caesarean delivery (both elective and emergency) compared to women with normal weight, but we note that the overall frequency of Caesarean delivery across our obstetric population seems quite high, compared with a previous Swedish study (10). We also found that women with overweight and obesity are at increased odds of labour induction. A very recent systematic review found that women with obesity are more likely than women with a normal weight to end labour induction with Caesarean delivery (22). Possible reasons could be that Caesarean delivery is probably less risky now, due to advances in medical science, which facilitate accurate monitoring of the progress of labour and the detection of fetal intra-partum conditions (23). It is also possible that

For peer review only

health professionals in Scotland are intervening earlier with regards to problems in labour among women with overweight or obesity, in order to reduce fetal distress, and its worst outcomes. Nevertheless, this pattern of very high Caesarean rates is concerning for Scotland, which has invested in programmes aimed at promoting natural birth, such as Keeping Childbirth Natural and Dynamic (KCND). The KCND is a maternity care programme introduced by the Scottish Government with the aim of maximising opportunities for women to have as natural a birth experience as possible, reduce unnecessary interventions in low-risk pregnancy and childbirth, and to provide women-centred care (24,25,26). The early intervention in pregnancy may also explain the reduced odds of low Apgar score for infants born to women with overweight and obesity. It is likely that these women may receive increased monitoring, which means issues can be identified and managed earlier, to reduce any fetal distress in labour.

Adiposity has also been found to increase odds of large-for-gestational age and macrosomia (27). In this study, we found that births to women with overweight and obesity were associated with increased odds of large-for-gestational age infants, compared with women of normal weight. Excess weight in pregnancy may shift the entire birth weight distribution upwards, perhaps through hormonal mechanisms that operate at lower levels, rather than in full-blown cases of macrosomia in infants of diabetic mothers. It is therefore unsurprising that high maternal BMI significantly decreased the odds of small-for-gestational age among our study population.

We found that pregnant women with obesity were at significantly increased odds of pre-term delivery, however the odds ratio was high but not statistically significant for

For peer review only

women with overweight. A systematic review examining the effect of maternal overweight and obesity on pre-term delivery showed that both women with overweight and obesity were at significantly higher risk of pre-term delivery (28). It has been shown that pre-eclampsia leads to pre-term delivery, especially in elective pre-term delivery (29). It is not clear why the odds ratio of pre-term delivery for women with overweight in our study population was not statistically significant. However, it is likely that the higher odds of pre-eclampsia in women with obesity, compared with women with overweight, could explain this finding. Regarding post-term delivery, there were no statistically significant odds ratios among women with both overweight and obesity. As discussed previously, it is likely that early intervention in pregnancy among our study population reduced the odds of the occurrence of post-term delivery.

We examined the association between high maternal BMI and placental abruption and placenta praevia, but found no statistically significant association between each of these two outcomes and overweight or obesity. This finding is congruent with a previous study (10). It appears that the relationships between maternal overweight and obesity, and both placental abruption and placenta praevia, may require further attention in future research.

Strengths and limitations

This study comprised a large, retrospectively accessed but cohort-structured, national database, covering several maternal and neonatal outcomes. The analyses used a population-wide data with adjustment for some confounders to estimate impact of high maternal-weight status on each outcome. We restricted the analyses

For peer review only - http://bmjopen.bmj.com/site/about/guidelines.xhtml

to only single births and first pregnancies to ensure that the births in the sample were relatively independent. The dataset we were provided with combined underweight and normal weight women as normal BMI group. Using this as the reference group might have strengthened the association between high maternal BMI and the pregnancy and neonatal outcomes considered. However, only a very small number of women are underweight during pregnancy in Scotland in recent years (2.8% in 2018/19) (30). Also, some studies differentiate between different obesity categories, but in this study the dataset we accessed did not differentiate these categories, and it was not possible to do this retrospectively; therefore all women with BMI of 30 or more were considered as having obesity. It is likely that differentiating morbid obesity, or obesity class II and III from women with obesity, would have generated additional insight, in the form of a full “dose response relationship”. The completeness of the recording of BMI increased during the study period (2008 to 2015) from 69% to 98%. Using data from the earlier years, when the BMI was missing more often, might have biased the study sample if BMI was not missing at random. In addition, mothers aged below 20 years and over 40 years were excluded from analyses due to the low numbers of cases in these age groups with obesity and experiencing adverse outcomes. The study controlled for a limited set of confounders, due to data availability; inclusion of other relevant confounders could have strengthened the analyses. For example, variables such as ethnicity, previous caesarean sections, and time of birth were not available in the dataset, which we accessed. Also, we could not analyse neonatal outcomes such as stillbirth, neonatal death and congenital anomaly because these outcomes are not completely ascertained in the dataset we used.

For peer review only - http://bmjopen.bmj.com/site/about/guidelines.xhtml

Conclusion

This study has shown that women who are overweight, and especially those who are obese in Scotland are at greater odds of several pregnancy and delivery complications including gestational hypertension, gestational diabetes, pre-eclampsia, labour induction and Caesarean delivery. The odds ratios of these conditions increased with increasing BMI. Health professionals should be empowered and trained to deliver promising dietary and lifestyle interventions to women at risk of overweight and obesity prior to conception, and control excessive weight gain in pregnancy.

References

1. Rooney BL, Schauberger CW. Excess pregnancy weight gain and long-term obesity: one decade later. *Obstet Gynecol* 2002; **100(2):** 245-252.
2. Kumari P, Gupta M, Kahlon P, Malviya S. Association between high maternal body mass index and fetomaternal Outcome. *J Obes Metab Res* 2014; **1:** 143-148.
3. Poobalan AS, Aucott LS, Gurung T, Smith WC, Bhattacharya S. Obesity as an independent risk factor for elective and emergency caesarean delivery in nulliparous women – systematic review and meta-analysis of cohort studies. *Obesity Reviews* 2009 10: 28-35. doi:10.1111/j.1467-789X.2008.00537.x
4. Rahman MM, Abe SK, Kanda M, Narita S, Rahman MS, Bilano V, Ota E, Gilmour S, Shibuya K. Maternal body mass index and the risk of birth and maternal health outcomes in low- and middle-income countries: a systematic review and meta-analysis. *Obesity Reviews* 2015; **16:** 758–770.

For peer review only - http://bmjopen.bmj.com/site/about/guidelines.xhtml

5. Godfrey KM, Reynolds RM, Prescott SL, Nyirenda M, Jaddoe VWV, Eriksson JG, Broekmann BFP. Influence of maternal obesity on the long-term health of offspring. *Lancet Diabetes Endocrinol* 2017; **5**: 53–64.
6. Morgan KL, Rahman MA, Hill RA, et al. Obesity in pregnancy: infant health service utilisation and costs on the NHS. *BMJ Open* 2015;5:e008357. doi:10.1136/bmjopen-2015- 008357.
7. Doi L, Williams AJ, Frank J. How has child growth around adiposity rebound altered in Scotland since 1990 and what are the risk factors for weight gain using the Growing Up in Scotland birth cohort 1? *BMC Public Health* 2016; **16:** 1081.
8. Denison FC, Norwood P, Bhattacharya S, Duffy A, Mahmood T, Morris C, Raja EA, Norman JE, Lee AJ, Scotland G. Association between maternal body mass index during pregnancy, short-term morbidity, and increased health service costs: a population-based study. *BJOG* 2014; **121**:72–82.
9. Smith GCS, Shah I, Pell JP, Crossley JA, Dobbie R. Maternal Obesity in Early Pregnancy and Risk of Spontaneous and Elective Preterm Deliveries: A Retrospective Cohort Study, *American Journal of Public Health* 2007; **97**(1): 157-162.
10. Cedergren MI. Maternal morbid obesity and the risk of adverse pregnancy outcome. *Obstetricians and Gynecologists* 2004; **103:** 219-224.
11. Information Service Division (2019). Data Dictionary. https://www.ndc.scot.nhs.uk/Dictionary-A-Z/
12. Information Service Division (2018). Scottish birth record. http://www.ndc.scot.nhs.uk/Data-Dictionary/SMR-Datasets/Scottish-Birth-Record/

For peer review only - http://bmjopen.bmj.com/site/about/guidelines.xhtml

13. Scottish Government (2019) NHS Scotland performance against LDP standard. https://www.gov.scot/publications/nhsscotland-performance-against-ldp-standards/pages/early-access-to-antenatal-services/

14. Cole TJ, Freeman JV, Preece MA. Body mass index reference curves for the UK, 1990. *Arch Dis Child* 1995; **73(1):** 25–29.

15. Freeman JV, Cole TJ, Chinn S, Jones PRM, White EM, Preece MA. Cross-sectional stature and weight reference curves for the UK 1990. *Arch Dis Child* 1995; **73(1):**17–24.

16. StataCorp. Stata statistical software: release 14. College Station: StataCorp LP; 2015.

17. Scott-Pillai R, Spence D, Cardwell C, Hunter A, Holmes V. The impact of body mass index on maternal and neonatal outcomes: a retrospective study in a UK obstetric population, 2004–2011. *BJOG* 2013; **120:** 932–939.

18. O'Brien TE, Ray JG, Chan WS. Maternal body mass index and the risk of preeclampsia: A systematic overview. *Epidemiology* 2003; **14:** 368-374.

19. Shieh C, Cullen DL, Pike C, Pressler SJ. Intervention strategies for preventing excessive gestational weight gain: systematic review and meta-analysis. *Obesity Reviews* 2018; doi: 10.1111/obr.12691.

20. Thangaratinam S, Rogozinska E, Jolly K, Glinkowski S, Roseboom T, Tomlinson JW, Kunz R, Mol BW, Coomarasamy A, Khan KS. Effects of interventions in pregnancy on maternal weight and obstetric outcomes: Meta-analysis of randomised evidence. *BMJ.* 2012; **344:** e2088.

21. Althabe F, Sosa C, Belizán JM, Gibbons L, Jacquerioz F, Bergel E. Cesarean section rates and maternal and neonatal mortality in low-, medium-, and high-income countries: an ecological study. *Birth* 2006; **33(4):** 270-277.

For peer review only - http://bmjopen.bmj.com/site/about/guidelines.xhtml

22. Ellis JA, Brown CM, Barger B. Carlson NS. (2019), Influence of Maternal Obesity on Labor Induction: A Systematic Review and Meta-Analysis. *Journal of Midwifery & Women's Health*, 2019; **64:** 55-67. doi:10.1111/jmwh.12935

23. Rezaie M, Shahoe R, Shahghebi S. The effect of maternal body mass index on the delivery route in nulliparous women. *Journal of Public Health and Epidemiology* 2013; **5(8):** 346-350.

24. Health Improvement Scotland (2009). Pathways for maternity care. http://www.healthcareimprovementscotland.org/default.aspx?page=12536

25. Abhyankar P, Cheyne H, Maxwell M, McCourt C. A realist evaluation of a normal birth programme. *Evidence Based Midwifery* 2013; https://www.rcm.org.uk/learning-and-career/learning-and-research/ebm-articles/a-realist-evaluation-of-a-normal-birth.

26. Cheyne H, Abhyankar P, McCourt C. Empowering change: realist evaluation of a Scottish Government programme to support normal birth. *Midwifery* 2013; **29:** 1110–1121.

27. Surkan PJ, Hsieh CC, Johansson AL , Dickman PW, Cnattingius S. Reasons for increasing trends in large for gestational age births. *Obstet Gynecol* 2004; **104**:720–6. doi:10.1097/01.AOG.0000141442.59573.cd

28. McDonald SD, Han Z, Mulla S, Beyene J. Overweight and obesity in mothers and risk of preterm birth and low birth weight infants: systematic review and meta-analyses *BMJ* 2010; 341 :c3428.

29. Hendler I, Goldenberg RL, Mercer BM, et al. The Preterm Prediction Study: association between maternal body mass index and spontaneous and indicated pre-term birth. *Am J Obstet Gynecol*. 2005; **192**:882–886.

For peer review only - http://bmjopen.bmj.com/site/about/guidelines.xhtml

30. ISD Scotland, Births In Scottish Hospitals - Year ending 31st March 2019 https://www.isdscotland.org/Health-Topics/Maternity-and-Births/Publications/data-tables2017.asp?id=2553#2553 Published 26th Nov 2019.

Funding

This work was funded by the SCPHRP core grant from the Medical Research Council (Grant Number MR/K023209/1) and the Chief Scientist Office of Scotland. AJW is supported by the European Centre for Environment and Human Health, University of Exeter. LM is supported by the Farr Institute @ Scotland, which is supported by a 10-funder consortium: Arthritis Research UK, the British Heart Foundation, Cancer Research UK, the Economic and Social Research Council, the Engineering and Physical Sciences Research Council, the Medical Research Council, the National Institute of Health Research, the National Institute for Social Care and Health Research (Welsh Assembly Government), the Chief Scientist Office (Scottish Government Health Directorates), (MRC Grant No: MR/K007017/1). The funders played no role in the conceptualisation or realisation of the research and no role in the decision to submit it for publication.

Competing interest

The authors declare that they have no competing interests.

Data sharing

For peer review only

Data used was categorised as confidential data release by the Electronic Data Research and Innovation Service of the Information Services Division, NHS National Services Scotland.

Acknowledgements

The authors are grateful to the Electronic Data Research and Innovation Service of the Information Services Division, NHS National Services Scotland for providing the data used in this paper.

Authors' contributions

LD, AJW and JF conceived the original idea for the study and obtained the data. AJW led the statistical analyses with support from LD, LM and JF. LD wrote the first draft of the paper and all authors revised successive drafts and approved the final manuscript.

For peer review only

For peer review only - http://bmjopen.bmj.com/site/about/guidelines.xhtml

Supplementary file 1: Description of data fields used in the study

Variable Name	Database Source	Variable Description/Values	Notes
Mother_ID	N/A - study specific	Unique mother identifier	1516-0613/' followed by an anonymous identifier
Baby_ID	N/A - study specific	Unique baby identifier	Mother_ID followed by delivery sequence number followed by a baby sequence number. The baby sequence number for multiple babies from same delivery not necessarily in correct order due to missing CHI numbers.
Delivery_Seq_No	N/A - study specific	Delivery sequence number of mother	
Gest_Diabetes	SMR02	**1:** Yes, Gestational diabetes (diagnosed during this pregnancy)<br>**0:** Yes, Pre-existing diabetes (diagnosed before pregnancy) & No (no diabetes during this pregnancy)<br>**Missing:** Yes, Time of diagnosis unknown & Not Known	http://www.ndc.scot.nhs.uk/Dictionary-A-Z/Definitions/index.asp?Search=D&ID=214&Title=Diabetes
Gest_Hypertension	SMR02/SMR01	**1:** ICD 10 code O13.X<br>**0:** All other codes	Flagged codes cover gestational hypertension
Pre_Eclampsia	SMR02/SMR01	**1:** ICD 10 code O14._<br>**0:** All other codes	Flagged codes cover pre-eclampsia
Placental_Abruption	SMR02/SMR01	**1:** ICD 10 code O45._<br>**0:** All other codes	Flagged codes cover placental abruption
Placental_Praevia	SMR02/SMR01	**1:** ICD 10 code O44._<br>**0:** All other codes	Flagged codes cover placenta praevia

For peer review only

Postpartum_Haemorrhage	SMR02/SMR01	**1:** ICD 10 code O72._<br>**0:** All other codes	Flagged codes cover postpartum haemorrhage
Caesarean_Delivery	SMR02	**1:** Elective (planned) caesarean section & Emergency and unspecified caesarean section<br>**0:** All other codes	http://www.ndc.scot.nhs.uk/Dictionary-A-Z/Definitions/index.asp?Search=M&ID=322&Title=Mode of Delivery - Babies 1 to 3
Labour_Induction	SMR02	**1:** 1-8 - Induction of labour codes<br>**0:** 0, None<br>**Missing:** 9, Not known	http://www.ndc.scot.nhs.uk/Dictionary-A-Z/Definitions/index.asp?Search=I&ID=295&Title=Induction of Labour
SGA	SMR02	**1:** Birthweight ≤10th percentile<br>**0:** Birthweight >10th percentile	Small for gestational age flag
LGA	SMR02	**1:** Birthweight ≥90th percentile<br>**0:** Birthweight <90th percentile	Large for gestational age flag
Preterm_Delivery	SMR02	**1:** Estimated gestation < 37 weeks<br>**0:** Estimated gestation ≥ 37 weeks and ≤ 42 weeks<br>**Missing:** otherwise	http://www.ndc.scot.nhs.uk/Dictionary-A-Z/Definitions/index.asp?Search=E&ID=242&Title=Estimated Gestation
Postterm_Delivery	SMR02	**1:** Estimated gestation > 42 weeks<br>**0:** Estimated gestation ≥ 37 weeks and ≤ 42 weeks<br>**Missing:** otherwise	http://www.ndc.scot.nhs.uk/Dictionary-A-Z/Definitions/index.asp?Search=E&ID=242&Title=Estimated Gestation
Apgar_Score	SMR02	**1:** Apgar score at 5 mins < 7<br>**0:** Apgar score at 5 mins ≥ 7	http://www.ndc.scot.nhs.uk/Dictionary-A-Z/Definitions/index.asp?Search=A&ID=88&Title=Apgar Score - Babies 1 to 3
Maternal_Obesity	SMR02	**1:** Obese status<br>**0:** Overweight, Healthy or underweight status	Adults with BMI ≥ 30 classed as obese. BMI of girls aged 2 - 19 years old standardized using UK1990 growth reference values and z-score ≥ 6/3 (98th centile) classed as obese.

For peer review only

Maternal_Overweight_Obesity	SMR02	**1:** Overweight or Obese status<br>**0:** Healthy or underweight status	Adults with BMI ≥ 25 classed as overweight or obese. BMI of girls aged 2 - 19 years old standardized using UK1990 growth reference values and z-score ≥ 4/3 (91st centile) classed as overweight or obese.
Age	SMR02	Age of mother at delivery (in years)	
Parity	SMR02	Total number of previous pregnancies	http://www.ndc.scot.nhs.uk/Dictionary-A-Z/Definitions/index.asp?Search=P&ID=409&Title=Previous Pregnancies
Deprivation	SMR02	Carstairs 2001 quintiles for Scotland	1=least deprived; 5=most deprived
Smoking_Status	SMR02	**1:** Yes<br>**0:** No<br>**Missing:** Not known	http://www.ndc.scot.nhs.uk/Dictionary-A-Z/Definitions/index.asp?Search=S&ID=456&Title=Smoker During Pregnancy
Multiple_births	SMR02	**1:** More than one birth this pregnancy<br>**0:** Single birth	http://www.ndc.scot.nhs.uk/Dictionary-A-Z/Definitions/index.asp?Search=N&ID=349&Title=Number of Births this Pregnancy
Multiple_births_in_NRS	NRS Births	**1:** Multiple babies found for this mother's delivery in NRS Births	Multiple babies recorded in NRS Births but only a single baby recorded in SMR02
Previous_Caesarean_section	SMR02	**1:** More than zero<br>**0:** Zero	http://www.ndc.scot.nhs.uk/Dictionary-A-Z/Definitions/index.asp?Search=P&ID=406&Title=Previous Caesarean Sections

For peer review only

Supplementary file 2. Study flow diagram

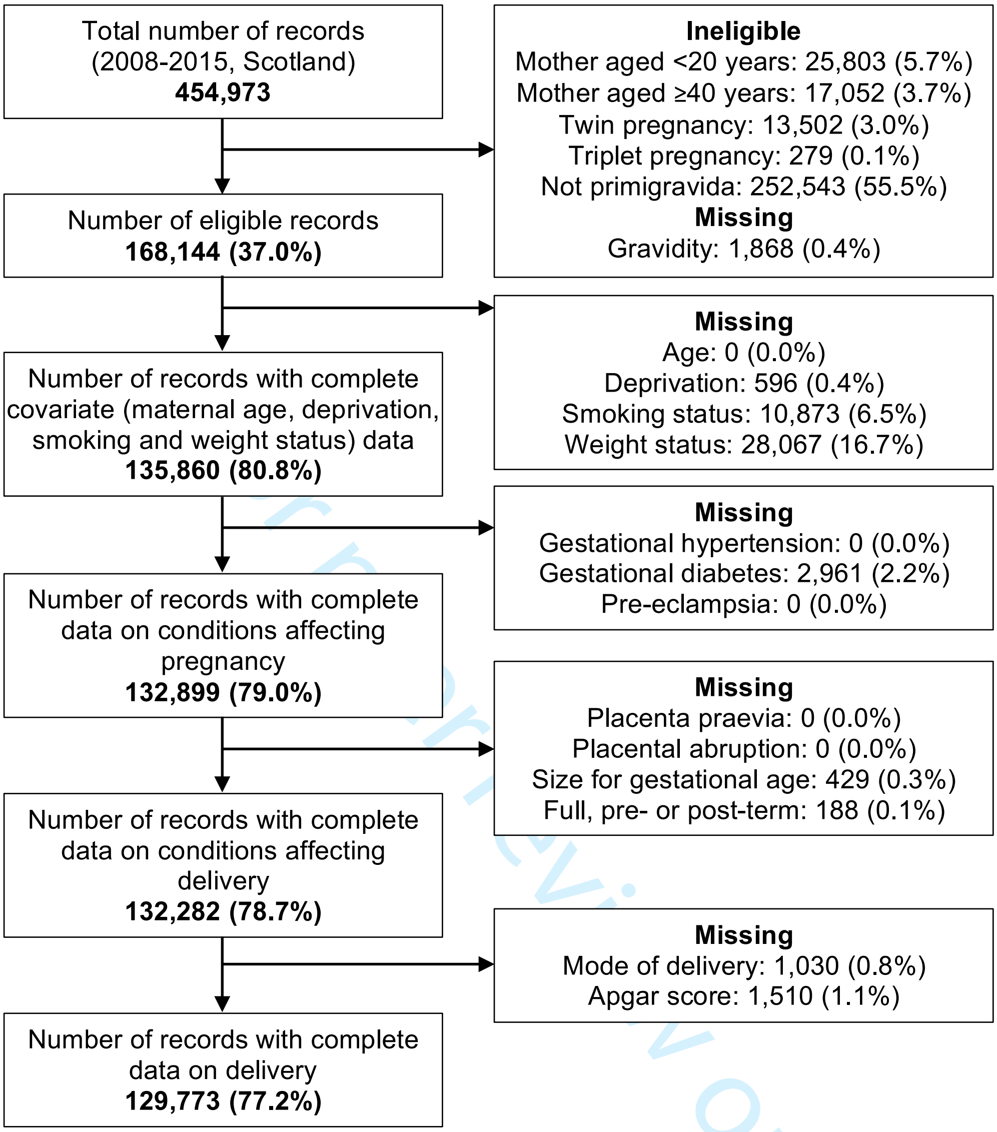

For peer review only

STROBE (Strengthening The Reporting of OBservational Studies in Epidemiology) Checklist

A checklist of items that should be included in reports of observational studies. You must report the page number in your manuscript where you consider each of the items listed in this checklist. If you have not included this information, either revise your manuscript accordingly before submitting or note N/A.

Note: An Explanation and Elaboration article discusses each checklist item and gives methodological background and published examples of transparent reporting. The STROBE checklist is best used in conjunction with this article (freely available on the Web sites of PLoS Medicine at http://www.plosmedicine.org/, Annals of Internal Medicine at http://www.annals.org/, and Epidemiology at http://www.epidem.com/). Information on the STROBE Initiative is available at www.strobe-statement.org.

Section and Item	Item No.	Recommendation	Reported on Page No.
**Title and Abstract**	1	(*a*) Indicate the study's design with a commonly used term in the title or the abstract	1
		(*b*) Provide in the abstract an informative and balanced summary of what was done and what was found	2
**Introduction**			
Background/Rationale	2	Explain the scientific background and rationale for the investigation being reported	4,5
Objectives	3	State specific objectives, including any prespecified hypotheses	5
**Methods**			
Study Design	4	Present key elements of study design early in the paper	5
Setting	5	Describe the setting, locations, and relevant dates, including periods of recruitment, exposure, follow-up, and data collection	5,6,7
Participants	6	(*a*) *Cohort study*—Give the eligibility criteria, and the sources and methods of selection of participants. Describe methods of follow-up	5,6,7
		Case-control study—Give the eligibility criteria, and the sources and methods of case ascertainment and control selection. Give the rationale for the choice of cases and controls	N/A
		Cross-sectional study—Give the eligibility criteria, and the sources and methods of selection of participants	N/A
		(*b*) *Cohort study*—For matched studies, give matching criteria and number of exposed and unexposed	N/A
		Case-control study—For matched studies, give matching criteria and the number of controls per case	N/A
Variables	7	Clearly define all outcomes, exposures, predictors, potential confounders, and effect modifiers. Give diagnostic criteria, if applicable	6,7

For peer review only

Section and Item	Item No.	Recommendation	Reported on Page No.
Data Sources/ Measurement	8*	For each variable of interest, give sources of data and details of methods of assessment (measurement). Describe comparability of assessment methods if there is more than one group	5,6,7
Bias	9	Describe any efforts to address potential sources of bias	8,9
Study Size	10	Explain how the study size was arrived at	5
Quantitative Variables	11	Explain how quantitative variables were handled in the analyses. If applicable, describe which groupings were chosen and why	8,9
Statistical Methods	12	(*a*) Describe all statistical methods, including those used to control for confounding	8,9
		(*b*) Describe any methods used to examine subgroups and interactions	N/A
		(*c*) Explain how missing data were addressed	7,16
		(*d*) *Cohort study*—If applicable, explain how loss to follow-up was addressed	N/A
		Case-control study—If applicable, explain how matching of cases and controls was addressed	N/A
		Cross-sectional study—If applicable, describe analytical methods taking account of sampling strategy	N/A
		(*e*) Describe any sensitivity analyses	N/A
**Results**			
Participants	13*	(a) Report numbers of individuals at each stage of study—eg numbers potentially eligible, examined for eligibility, confirmed eligible, included in the study, completing follow-up, and analysed	11
		(b) Give reasons for non-participation at each stage	N/A
		(c) Consider use of a flow diagram	Figure 1
Descriptive Data	14*	(a) Give characteristics of study participants (eg demographic, clinical, social) and information on exposures and potential confounders	5,6,7,8
		(b) Indicate number of participants with missing data for each variable of interest	11
		(c) *Cohort study*—Summarise follow-up time (eg, average and total amount)	5,6
Outcome Data	15*	*Cohort study*—Report numbers of outcome events or summary measures over time	6,7
		Case-control study—Report numbers in each exposure category, or summary measures of exposure	N/A
		Cross-sectional study—Report numbers of outcome events or summary measures	N/A

For peer review only

Section and Item	Item No.	Recommendation	Reported on Page No.
Main Results	16	(*a*) Give unadjusted estimates and, if applicable, confounder-adjusted estimates and their precision (eg, 95% confidence interval). Make clear which confounders were adjusted for and why they were included	9,10,11,12,13
		(*b*) Report category boundaries when continuous variables were categorized	8
		(*c*) If relevant, consider translating estimates of relative risk into absolute risk for a meaningful time period	N/A
Other Analyses	17	Report other analyses done—eg analyses of subgroups and interactions, and sensitivity analyses	N/A
**Discussion**			
Key Results	18	Summarise key results with reference to study objectives	13
Limitations	19	Discuss limitations of the study, taking into account sources of potential bias or imprecision. Discuss both direction and magnitude of any potential bias	16
Interpretation	20	Give a cautious overall interpretation of results considering objectives, limitations, multiplicity of analyses, results from similar studies, and other relevant evidence	13,14,15
Generalisability	21	Discuss the generalisability (external validity) of the study results	13
**Other Information**			
Funding	22	Give the source of funding and the role of the funders for the present study and, if applicable, for the original study on which the present article is based	20

*Give information separately for cases and controls in case-control studies and, if applicable, for exposed and unexposed groups in cohort and cross-sectional studies.

Once you have completed this checklist, please save a copy and upload it as part of your submission. DO NOT include this checklist as part of the main manuscript document. It must be uploaded as a separate file.

For peer review only
